# New Aspects of Uptake and Metabolism of Non-organic and Organic Iodine Compounds—The Role of Vanadium and Plant-Derived Thyroid Hormone Analogs in Lettuce

**DOI:** 10.3389/fpls.2021.653168

**Published:** 2021-04-16

**Authors:** Sylwester Smoleń, Małgorzata Czernicka, Iwona Kowalska, Kinga Kȩska, Maria Halka, Dariusz Grzebelus, Marlena Grzanka, Łukasz Skoczylas, Joanna Pitala, Aneta Koronowicz, Peter Kováčik

**Affiliations:** ^1^Department of Plant Biology and Biotechnology, Faculty of Biotechnology and Horticulture, University of Agriculture in Krakow, Kraków, Poland; ^2^Department of Plant Product Technology and Nutrition Hygiene, Faculty of Food Technology, University of Agriculture in Krakow, Kraków, Poland; ^3^Laboratory of Mass Spectrometry, Faculty of Biotechnology and Horticulture, University of Agriculture in Krakow, Kraków, Poland; ^4^Department of Human Nutrition and Dietetics, Faculty of Food Technology, University of Agriculture in Krakow, Kraków, Poland; ^5^Department of Agrochemistry and Plant Nutrition, Slovak University of Agriculture in Nitra, Nitra, Slovakia

**Keywords:** 5-iodosalicylic acid, 3, 5-diiodosalicylic acid, vHPO, CBL-interacting serine/threonine-protein kinase 6, plant-derived thyroid hormone analogs

## Abstract

The process of uptake and translocation of non-organic iodine (I) ions, I^–^ and IO_3_^–^, has been relatively well-described in literature. The situation is different for low-molecular-weight organic aromatic I compounds, as data on their uptake or metabolic pathway is only fragmentary. The aim of this study was to determine the process of uptake, transport, and metabolism of I applied to lettuce plants by fertigation as KIO_3_, KIO_3_ + salicylic acid (KIO_3_+SA), and iodosalicylates, 5-iodosalicylic acid (5-ISA) and 3,5-diiodosalicylic acid (3,5-diISA), depending on whether additional fertilization with vanadium (V) was used. Each I compound was applied at a dose of 10 μM, SA at a dose of 10 μM, and V at a dose of 0.1 μM. Three independent 2-year-long experiments were carried out with lettuce; two with pot systems using a peat substrate and mineral soil and one with hydroponic lettuce. The effectiveness of I uptake and translocation from the roots to leaves was as follows: 5-ISA > 3,5-diISA > KIO_3_. Iodosalicylates, 5-ISA and 3,5-diISA, were naturally synthesized in plants, similarly to other organic iodine metabolites, i.e., iodotyrosine, as well as plant-derived thyroid hormone analogs (PDTHA), triiodothyronine (T3) and thyroxine (T4). T3 and T4 were synthesized in roots with the participation of endogenous and exogenous 5-ISA and 3,5-diISA and then transported to leaves. The level of plant enrichment in I was safe for consumers. Several genes were shown to perform physiological functions, i.e., *per64-like*, *samdmt*, *msams5*, and *cipk6.*

## Introduction

### Iodine in Plants

Iodine (I) is a beneficial element for plants and studies have determine the effectiveness of iodide (I^–^) or iodate (IO_3_^–^) uptake by plants through roots or leaves (upon foliar application) and their potential translocation (Medrano-Macías et al., 2016; Antonyak et al., 2018). The effectiveness of I ion accumulation has been determined for a number of species of plants. The majority of the studies have been performed in the context of I biofortification of plants. They were conducted with the aim to prepare an I deficiency prevention program, other than through the consumption of kitchen salt based on plant enrichment with I (White and Broadley, 2009). Among the papers published within the last 15 years, there has been research on species such as kohlrabi (Golob et al., 2020), strawberry (Budke et al., 2020), lettuce (Blasco et al., 2008; Dávila-Rangel et al., 2020), basil (Incrocci et al., 2019; Kiferle et al., 2019), green bean, lettuce (Dobosy et al., 2020), cabbage, cowpea (Ojok et al., 2019), broccoli raab, curly kale, mizuna, and red mustard (Gonnella et al., 2019). There was also research devoted to the I uptake mechanism (Kato et al., 2013; Humphrey et al., 2019).

Another subject of research was the impact of I on oxyreduction, e.g., in lettuce (Blasco et al., 2008; Dávila-Rangel et al., 2020), the process of photosynthesis in kohlrabi (Golob et al., 2020) and basil (Kiferle et al., 2019), nitrate (V) content in four *Brassica* genotypes (Gonnella et al., 2019), and changes in the content of macro- and microelements in green bean and lettuce (Dobosy et al., 2020). Furthermore, there was also research devoted to the efficacy of I^–^ and/or IO_3_^–^ uptake by plants, depending on the additional application of other elements, such as selenium in kohlrabi (Golob et al., 2020), zinc, selenium, and iron in wheat (Zou et al., 2019), and zinc and selenium in wheat and rice (Cakmak et al., 2020). The impact of I on the induction of plant resistance to diseases was also analyzed (Ajiwe et al., 2019). There are also works focused on the process of methylation, i.e., volatilization to the atmosphere, of volatile I forms (Leblanc et al., 2006; Itoh et al., 2009).

There were also studies that tackled a unique subject of the plants’ ability to take up I applied in the form of organic compounds, e.g., iodoacetate anion (Weng et al., 2008) or organic I complexes. For instance, Dávila-Rangel et al. (2020) showed that the application of the chitosan-I complex enhanced I uptake by lettuce. The ability of tomato plants to take up organic I compounds was also determined, in which I was bound with the aromatic ring (Halka et al., 2019). The conclusion for lettuce was that the effectiveness of I biofortification with 5-ISA was higher than upon application with KIO_3_ (Smoleń et al., 2017). Smoleń et al. (2020) showed the enrichment of lettuce with I using KIO_3_, 5-ISA, and 3,5-diISA; however, they did not study the molecular mechanisms associated with the process of uptake and metabolism of these three I compounds.

Previous literature on physiology and/or biochemistry of plants has not considered the issue of activity and function of plant-derived thyroid hormone analogs (PDTHA), which are compounds that contain I; hence, the scarcity of scientific data on the subject. Fowden (1959) demonstrated that radioactive I binds to I organic compounds, e.g., T3 in bean, barley, aster, and Salicornia plants. Lima et al. (2012) presented general information on the potential presence of PDTHA compounds in plants. Based on their model research, Pessoa et al. (2010) concluded that in *Arabidopsis thaliana* exogenous T4 may be bound by the transthyretin-like protein. Plants were observed to produce transthyretins, i.e., proteins that may potentially act as T3 and/or T4 transporters (Eneqvist et al., 2003). However, in the available literature, it is unclear whether T3/T4 receptors might be present in plants, and descriptions of the potential genes that might encode such proteins is lacking. Obtaining information on the issue seems crucial for the determination of functionality and mechanism of PDTHA activity in plants.

### Vanadium vs. Iodine

Vanadium is classified as a beneficial element for plants (Welch and Huffman, 1973; Mengel and Kirkby, 1996) and is also beneficial for animals and humans (Anke et al., 2002). In the human body, V regulates the activity of a number of enzymes (WHO, 2001; Anke et al., 2002; Gruzewska et al., 2014). It also improves thyroid function (Afkhami et al., 2009). There is no recommended dietary allowance (RDA) established for humans (Trumbo et al., 2001). The official information on the effect of V on humans was issued by WHO (World Health Organization) several decades ago and only contains rough suggestions concerning V doses for humans (10 μg V⋅24 h^–1^).

A positive impact of V on the growth and development of plants was observed at low doses < 0.04 mg V⋅dm^–3^ in the nutrient solution (Kaplan et al., 1990; Pilbeam and Drihem, 2007). Such doses have been observed to have a synergistic effect on the uptake of selected macroelements by plants or to increase the foliar content of photosynthetically active pigments (Kaplan et al., 1990; Pilbeam and Drihem, 2007). Higher photosynthetic activity following V application results in the accumulation of sugars in sweetcorn (Sentíes-Herrera et al., 2018), leading to larger biomass growth in the aboveground parts of the plants (Basiouny, 1984).

In hydroponic systems, the availability of V for roots is higher than that in the soil. Therefore, the V tolerance/harmfulness for plants (Gil et al., 1995; Chongkid et al., 2007; Vachirapatama et al., 2011) is much lower than that in the soil (Zhang et al., 2012; Akoumianaki-Ioannidou et al., 2016; Imtiaz et al., 2018). Next to the dose, the plants’ response to V also depends on I oxidation and is a generic property of plants (Kaplan et al., 1990; Gil et al., 1995; Vachirapatama et al., 2011). The low root absorbability of V is due to its poor mobility in the soil (Cappuyns and Swennen, 2014). The process of V sorption is related to the fact that VO_2_^+^ very easily reacts with humic acids in soil organic matter (SOM), making V not easily available to plants (Pilbeam and Drihem, 2007). Vanadium has not been commonly included in the process of preparing nutrient solutions for hydroponic systems (Jones, 2016). The conducted research made it possible to determine the plant’s response to the interaction between the simultaneous fertilization of plants with I and V.

In marine algae, V functions in I uptake into cells. This functionality of V is attributed to its presence at the active site of iodoperoxidase and other haloperoxidases (vHPO), i.e., V-dependent bromoperoxidase or chloroperoxidase (Leblanc et al., 2006). The structure, regulatory activity, and functionality of vHPO was described, owing to research on a number of marine alga species (Almeida et al., 2000; Colin et al., 2003; Kongkiattikajorn and Pongdam, 2006; Leblanc et al., 2006). Haloperoxidases (HPOs) are responsible for the oxidation of halogens that was conducted in the presence of H_2_O_2_ (Colin et al., 2005).

The process of cell I uptake by marine algae, catalyzed by vHPO, consisted of the oxidation of I^–^ to HIO, which was further transformed to molecular I_2_. HIO and I_2_ are produced within the cell wall. Being more lipophilic than I^–^, they easily penetrate through the wall to the cytosol (Leblanc et al., 2006). Medrano-Macías et al. (2016) reported that there is likely a relationship between I and V in terrestrial plants. However, the function of vHPO in domesticated plants is not known. Smoleń et al. (2020) identified vHPO-like enzyme activity in lettuce and a relationship between its activity and I root uptake in the setting of trace I content in the rhizosphere. Thus far, no gene in the genome of lettuce has been assigned the function of vHPO.

The response of plants to I and V application on physiological and biochemical properties has not been diagnosed. The issue has been, to a limited extent, described in preliminary research by Smoleń et al. (2020). This situation is different for marine algae, as these plants actively take up I, accumulate it in their tissues, and carry out methylation (Leblanc et al., 2006; Keng et al., 2020). The methylation process (I volatilization) has also been described for selected terrestrial plant species (Attieh et al., 2000; Nagatoshi and Nakamura, 2007; Itoh et al., 2009). Among the studies conducted were biotechnological studies on deactivation of the process in *A. thaliana* (Landini et al., 2012). Iodovolatilization is carried out with the participation of vHPO. In contrast, the methylation of iodic hydrocarbons (CH_*x*_I_*x*_) is carried out with the participation of S-adenosyl-l-methionine (SAM)-dependent halide methyltransferase (HMT) or SAM-dependent halide/thiol methyltransferase (HTMT). These enzymes use iodide as a substrate (Medrano-Macías et al., 2016; Gonzali et al., 2017). No gene encoding HMT or HTMT has been identified in the genome of lettuce.

### Salicylic Acid (SA) and SA-Derivatives vs. Iodine

A volatile ester of methyl salicylic acid (MeSA) can be volatilized in roots and leaves. MeSA is produced in the process of esterification of salicylic acid, during which CH_3_ is joined to the SA carboxylic group (Taiz and Zeiger, 2010; Zhang et al., 2013). In tomatoes, MeSA is synthesized by an enzyme named salicylic acid carboxyl methyltransferase (SAMT) (Tieman et al., 2010). This volatile ester participates in SAR (systemic acquired resistance) in plants (Gao et al., 2014). SA is classified as a plant phytohormone (Gust and Nürnberger, 2012) or as a phytohormone-like compound (Hayat et al., 2010). Literature lacks information on whether endogenous and exogenous iodosalicylates, such as 5-ISA and 3,5-diISA, in plants may undergo further catabolic reactions. No enzymes related to the catabolism of 5-ISA and 3,5-diISA have been identified to date in plants. Additionally, there is no data indicating whether SAMT can participate in the methylation of 5-ISA, 3,5-diISA, or the SA produced as a result of the potential degradation of these iodosalicylates. Moreover, genes encoding proteins with a SAMT-like function in lettuce have not been found.

The aim of this study was to determine the process of uptake and metabolism of I applied to plants as KIO_3_ and iodosalicylates. Additionally, the study aimed to determine the effect of V on these processes. Another objective was to document the selected molecular processes in the metabolism of non-organic and organic I compounds in roots and leaves of lettuce, considering aspects related to the synthesis of PDTHA.

A novelty in this study, when compared with previously published ones, was research on the plants’ ability to take up non-organic and organic I compounds (iodosalicylates) through the roots, as well as whether and to what extent these compounds can be metabolized and transported within the plants’ roots-leaves system. Additionally, selected genes were examined and assigned a potential putative role for encoding enzyme proteins demonstrating functions typical of vHPO, SAMT, and HMT/HTMT.

## Materials and Methods

### Plant Material and Treatments

*Lactuca sativa* L. var. *capitata* cv. “Melodion” was planted in two pot studies and one hydroponic study. This research was performed within the camp of the University of Agriculture in Kraków (50°05′04.1″N 19°57′02.1″E).

A nutrient film technique (NFT) was used for the hydroponic system in a greenhouse setting. The hydroponic experiment was named Experiment 1 (Table 1). The pot studies were in turn conducted in a foil tunnel with the plants being farmed in 2 types of substrate, a peat substrate as organic soil (Experiment 2) and loam soil as an example of a heavy mineral soil (Experiment 3). Each of the 3 experiments was repeated twice in the spring season during 2 consecutive years of the study in 2018 and 2019.

**TABLE 1:** Design and method of conducting experiments with lettuce cultivation in the hydroponics NFT Experiment No. 1 as well as in pot experiments: Experiment Nos. 2 and 3.

Treatments	Hydroponics NFT Experiment No. 1					
	Dose of I compounds and dose of I	Dose of V as ammonium metavanadate	Dose of SA	I, V, and/or SA application from the rosette phase	Amount of I applied for one plant (μ mol I⋅plant^–1^)	Amount of V applied for one plant (μ mol V⋅plant^–1^)
Control	–*	–*	–	–	13.2	0.09
SA	–*	–*	10 μM	Permanent	13.2	0.09
KIO_3_	10 μM (10 μM I)	–	–	Permanent	100	0.09
KIO_3_+SA	10 μM (10 μM I)	–	10 μM	Permanent	100	0.09
5-ISA	10 μM (10 μM I)	–	–	Permanent	100	0.09
3,5-diISA	10 μM (20 μM I)	–	–	Permanent	200	0.09
KIO_3_+V	10 μM (10 μM I)	0.1 μM V	–	Permanent	100	0.98
KIO_3_+SA+V	10 μM (10 μM I)	0.1 μM V	10 μM	Permanent	100	0.98
5-ISA+V	10 μM (10 μM I)	0.1 μM V	–	Permanent	100	0.98
3,5-diISA+V	10 μM (20 μM I)	0.1 μM V	–	Permanent	200	0.98

	**Pot experiments: Peat substrate Experiment No. 2 and mineral soil Experiment No. 3**					

Control	–**	–**	–	–	–****	–****
SA	–**	–**	10 μM	8 times***	–****	–****
KIO_3_	10 μM (10 μM I)	–	–	8 times***	2.67	–****
KIO_3_+SA	10 μM (10 μM I)	–	10 μM	8 times***	2.67	–****
5-ISA	10 μM (10 μM I)	–	–	8 times***	2.67	–****
3,5-diISA	10 μM (20 μM I)	–	–	8 times***	5.34	–****
KIO_3_+V	10 μM (10 μM I)	0.1 μM V	–	8 times***	2.67	0.027
KIO_3_+SA+V	10 μM (10 μM I)	0.1 μM V	10 μM	8 times***	2.67	0.027
5-ISA+V	10 μM (10 μM I)	0.1 μM V	–	8 times***	2.67	0.027
3,5-diISA+V	10 μM (20 μM I)	0.1 μM V	–	8 times***	5.34	0.027

*Trace concentration of I and V in nutrient solution: **0.0204 μM I** and **0.009 μM V** of nutrient solution (I and V from tap water and fertilizers).**Without I and V fertilization—the natural content of I and V in peat substrate and mineral soil prior to the Experiments No 2 and 3 was presented in Table 2.***8 times every 7 days.****The determined I and V content (in TMAH and metricconverterProductID1 M1 M HCl extracts, respectively see Table 2) do not allow estimating the amount of available I and V for plants in the soil solution of peat substrate (organic soil) and mineral soil. It is also not possible to accurately estimate the changes in the content of I and V in the soil solution during the whole period of lettuce cultivation using other methods of soil extraction.

The subject of this study was plant fertilization with I (Table 1), that is, with potassium iodate (KIO_3_), 5-iodosalicylic acid (5-ISA), and 3,5-diiodosalicylic acid (3,5-diISA), as well as with ammonium metavanadate (V). An additional application of salicylic acid (SA) was also used to compare the effects of both iodosalicylic acids. The same configuration of the combinations tested was used in all 3 experiments: (1) Control; (2) SA; (3) KIO_3_; (4) KIO_3_+SA; (5) 5-ISA; (6) 3,5-diISA; (7) KIO_3_ + V; (8) KIO_3_ + SA + V; (9) 5-ISA + V; and (10) 3,5-diISA + V. In each experiment, application started at the rosette stage (5–6 true leaves). The following concentrations were used: 10 μM for all I compounds (molar mass equivalents), 10 μM for SA, and 0.1 μM for V. The experiments differed in the frequency of application. In hydroponic systems, the nutrient solution (containing the compounds tested) was applied continually (fixed concentration). A different application strategy was used in the pot systems. The I and V compounds and SA were applied to the soil once a week through manual fertigation (manual watering with solutions of the compounds studied, at a dose of 100 mL⋅pot^–1^ (one plant^–1^). In total, in Experiments 2 and 3, plants were fertilized with I, V, and SA 8 times. This research strategy was planned purposefully. Our aim was to avoid the risk of accumulation of I concentrations that would be toxic to plants in either the peat substrate or mineral soil. When designing the study, we had no information on whether or to what extent iodosalicylates applied to the soil would be taken up by the plants. Additionally, the aim was to measure the effectiveness of biofortification of lettuce using different I compounds with or without V, depending on the method of cultivation and substrate.

In a hydroponic system, it was possible to obtain the entire root system for chemical analyses without damaging it, among other things. This allowed model research on the uptake and transport of different forms of I in the roots-leaves system. In the pot system, it was impossible to isolate roots from the soil because the root system outgrew the volume of soil in the pots. Therefore, in Experiments 2 and 3, roots were not subjected to chemical analyses.

In each year of the study, seeds were sown in early March (13 March 2018 and 4 March 2019). They were sown in multi-pallets filled with substrate, that is, with peat substrate and sand 1:1 (V/V). The multi-pallets had 112 cells (14 rows × 8 cells), each sized 3.2 × 3.2 × 4 cm. Young plants in the phase of 4–5 true leaves were replanted to the NFT hydroponic system (in Experiment 1) or to pots (in Experiments 2 and 3; 10 April 2018 and 2 April 2019). The plants were potted together with the entire root ball in either peat substrate or heavy mineral soil. The volume of substrate in pots was 1.5 dm^3^. The chemical properties of the peat substrate and heavy mineral soil before cultivation are presented in Table 2; a detailed description of methods used for chemical analysis of soil is reported in the Supplementary Material. No fertilization was performed before or during lettuce cultivation in either of the two pot experiments. This was because the nutrient content, pH, and EC (electrical conductivity) were optimal for growing this species in the peat substrate or heavy mineral soil I (Sady, 2000; Table 2).

**TABLE 2:** Selected chemical properties of the soil prior to the Experiments Nos. 2 and 3.

Physicochemical soil characteristic	Peat substrate—Experiment 2	Mineral soil—Experiment 3
pH_H_2_O_	5.50	6.70
pH_(KCl)_	5.33	6.28
EC (mS⋅cm^–1^)	1.18	1.46
Eh(mV)	270.1	221.6
Macroelements:		
N-NH_4_ (mg⋅dm^–3^)	158.7	68.2
N-NO_3_ (mg⋅dm^–3^)	103.1	205.9
N-NH_4_+ N-NO_3_ (mg⋅dm^–3^)	261.8	274.1
P (mg⋅dm^–3^)	125.1	94.5
K (mg⋅dm^–3^)	316.8	122.2
Mg (mg⋅dm^–3^)	180.0	175.4
Ca (mg⋅dm^–3^)	2 046.2	1 686.8
S (mg⋅dm^–3^)	227.8	287.4
Na (mg⋅dm^–3^)	20.6	55.9
Iodine (mg I⋅kg^–1^)	6.25	5.56
Vanadium (mg V⋅kg^–1^)	0.84	6.57
Al-hydroxides (mg⋅kg^–1^)	145.8	400.53
Fe-hydroxides (mg⋅kg^–1^)	624.3	5 118.0
Mn-hydroxides (mg⋅kg^–1^)	15.4	433.7
BeA (mg⋅kg^–1^)	1.768	0.864
SA (mg⋅kg^–1^)	0.156	0.034
5-ISA (mg⋅kg^–1^)	0.0125	0.0036
3,5-diISA (mg⋅kg^–1^)	0.008	0.008
2-IBeA (mg⋅kg^–1^)	0.028	0.013
4-IBeA (mg⋅kg^–1^)	0.001	0.002
2,3,5-triIBeA (mg⋅kg^–1^)	0.013	0.026
Exchange hydrolytic acidity (me⋅100 g^–1^)	0.99	22.13
Cation exchange capacity (me⋅100 g^–1^)	21.03	47.62
Total soil sorption capacity (me⋅100 g^–1^)	22.02	69.74
Soil organic matter (%)	100	1.92
Soil texture (according to the ISSS classification).	Peat soil (organic soil)	Loam soil 35 sand 28 % silt 37% clay

*Where: benzoic acid (BeA), salicylic acid (SA), 5-iodosalicylic acid (5-ISA), 3,5-diiodosalicylic acid (3,5-diISA), 2-iodobenzoic acid (2-IBeA), 4-iodobenzoic acid (4-IBeA), 2,3,5-triiodobenzoic acid (2,3,5-triIBeA).*

In the NFT hydroponic system, target replanting was preceded by thorough rinsing of the substrate between the seedlings’ roots with tap water. Seedlings were placed in the openings (spaced 25 cm apart) in Styrofoam slabs filling the NFT beds. No substrate was used in slabs. Once planted, seedlings in the NFT system were watered during the day. Each experiment consisted of four replicates in a randomized block design. In hydroponic Experiment 1, there were 15 plants per replicate and 60 plants per combination (600 plants per experiment). In pot Experiments 2 and 3, there were 7 plants per replicate and 28 plants per combination (a total of 560 plants in both pot experiments).

The type of nutrient solution, its preparation (content of macro- and microelements), regulation of pH and EC, and type of fertilizers in the NFT were the same as in our previous studies with lettuce in the same hydroponic system (Smoleń et al., 2019b). The I used in the base nutrient solutions (control) was iodide I^–^ (25.52 μg I⋅dm^–3^) and iodate IO_3_^–^ (0.29 μg I⋅dm^–3^). The content of I was natural (from water and dissolved fertilizers).

Once planted in spacers in the NFT systems, the plants were watered during the day between 5 am and 7 pm and at night between 1 and 2 am, for 1 min at 5-min intervals. In pot experiments, the plants were watered with tap water using drip irrigation. A single dose of water was about 100 mL⋅plant^–1^ (pot^–1^). The frequency of watering was adjusted to weather conditions and sizes of the plants; factors that determined the rate of substrate drying involved controlling watering by the irrigation computer with the option to sum the amount of solar radiation to start irrigation. The adopted watering strategy made it possible to eliminate water leaching from pots. On the days when the compound solutions were applied to the substrate in pot Experiments 2 and 3, the plants were not watered through the drip system.

Plants were harvested at the phase of head development, that is on 15 May 2018 and 7 May 2019 in hydroponic Experiment 1, and on 9 May 2018 and 16 May 2019 in pot Experiments 2 and 3. Then, the heads (lettuce leaves) were weighed. Hydroponic Experiment 1 was the only experiment where lettuce leaf harvesting was immediately followed by pipette collection of a secretion produced as a result of root pressure [white secretion (RootSec) on the surface of the root neck, visible after cutting the heads at collar level (lettuce leaves), as shown in Supplementary Figure 1]. The secretion was collected for the determination of the chemical forms of I, transported from roots to the aboveground parts of plants. A total of two samples were collected for each combination, each containing 5 mL of root secretion. Immediately after collection, the secretion was 1:1 mixed with buffer (20 mM Tris HCl buffer, pH 8.5). Then, the samples were frozen at −20°C and stored until analyzed using two mass spectrometry techniques. Iodides (I^–^) and iodates (IO_3_^–^) were analyzed using high-performance liquid chromatography with inductively coupled plasma mass spectrometry (HPLC-ICP-MS)/MS, while organic I compounds were analyzed using liquid chromatography-mass spectrometry (LC-MS)/MS. Methodologic details are presented in the following subsections.

In Experiment 1, the collection of root secretions was followed by the measurement of lettuce root biomass, while in pot Experiments 2 and 3 these measurements were abandoned, as the root biomass could not be separated from the soil without damaging the roots. Chemical analyses were performed on roots of all plants from the hydroponic system, and on four randomly selected heads from each biological replicate in all three experiments.

### Activity of Vanadium-Dependent Haloperoxidases

Fresh leaf samples collected in all three experiments and root samples from Experiment 1 were used to measure the total activity of vHPO enzymes. The analysis was performed with a method adapted for lettuce by Smoleń et al. (2020) based on that for marine algae. The activity of vHPO was calculated based on the increase in absorbance within 20 min (wavelength: 620 nm) and converted into U⋅mg^–1^⋅min^–1^ protein. Protein content in enzyme extracts was measured using the Lowry method (Waterborg, 2002). Bovine serum albumin was used as a standard.

### Freeze-Drying of Samples

Fresh root and leaf samples (lettuce) were washed in tap and distilled water. Samples of lettuce heads were vacuum-dried. Each head was halved (leaves were peeled in each growth phase) and mixed thoroughly as part of each replicate. Root and leaf samples were frozen at −20°C. The freeze-drying of frozen samples was performed with the Christ Alpha 1–4 unit (Martin Christ Gefriertrocknungsanlagen GmbH, Germany). Vacuum-dried samples of roots and leaves were ground in a lab mill (FRITSCH Pulverisette 14; FRITSCH GmbH, Weimar, Germany) and stored in sealed polyethylene bags until further chemical analyses (described in the next three sections).

### Analysis of Total Iodine and Vanadium in Dry Samples of Roots and Leaves

The analysis of I content in samples of lettuce leaves and roots was performed by inductively coupled plasma mass spectrometry (ICP-MS/MS) with a triple quadruple spectrometer (iCAP TQ ICP-MS Thermo Fisher Scientific, Bremen, Germany), preceded by alkaline extraction of 0.2 g samples by tetramethylammonium hydroxide (TMAH; Smoleń et al., 2019a, c; based on Pn-En 15111, 2008).

Vanadium content in leaf and root samples was measured using inductively coupled plasma optical emission spectrometry (ICP-OES) (Prodigy Spectrometer, Leeman Labs, New Hampshire, MA, United States). The mineralization and measurement procedures were consistent with the method described by Smoleń et al. (2020).

The results of I and V content in the plant samples and biomass measurements were used to calculate I uptake (I-uptake) and V uptake (V-uptake) by plants.

### Analysis of Iodides (I^–^) and Iodates (IO_3_^–^) in Roots and Leaves by HPLC-ICP-MS/MS

The content of iodides (I*^–^*) and iodates (IO_3_*^–^*) was only determined in root and leaf samples of hydroponic lettuce (Experiment 1); in pot experiments, there was no possibility to collect root samples for analysis. The content of these I ions was measured using a modified extraction procedure described by Smoleń et al. (2016). Briefly, a 0.05 g analytic portion of air-dried, ground plant samples was extracted (in 7 mL polypropylene tubes) using a solution containing 4 mL 25% TMAH (Sigma-Aldrich, St. Louis, MO, United States) and 10 mL 0.1 M NaOH (Chempur, Piekary Śla̧skie, Poland) dissolved to a final volume of 1 L with demineralized water. Once mixed, the samples were incubated for 1 h at 50°C in an ultrasonic bath, then cooled to approximately 20°C, mixed thoroughly, and centrifuged for 15 min at 4,500 rpm. The supernatants were filtered through a 0.22 μm syringe filter. The content of I ions in filtered samples was analyzed using HPLC-ICP-MS/MS. For I^–^ and IO_3_*^–^* speciation forms, HPLC (Thermo Scientific Ultimate 3000; Thermo Fisher Scientific, Bremen, Germany) was coupled to ICP-MS/MS (iCAP TQ). This method employed a strong anion exchange column [Thermo Fisher Scientific; Dionex IonPac AS11 (4 × 250 mm)] and a precolumn [Thermo Fisher Scientific; Dionex IonPac AG11 (4 × 50 mm)]. The column temperature was set to 30°C. Demineralized water, 50 mM NaOH, and 0.5% TMAH were used as eluents. To separate both I ions, a mobile phase, containing 2.5 mM NaOH and 0.125% TMAH at an isocratic flow, was used. The flow-rate was 1.5 mL/min, with an injection volume of 10 μL and total analysis time of 7 min (Supplementary Figures 2, 3). The HPLC-column effluent was introduced directly into ICP-MS/MS (iCAP TQ ICP-MS). Iodine was determined at 127I.16O isotope, using S-TQ-O2 mode. Standards were prepared through dissolution of KI and KIO_3_ (Sigma-Aldrich, St. Louis, MO, United States) in demineralized water.

To ensure correct iodide and I measurements, a “standard addition method” was used; it was applied independently for each leaf and root sample from each of the 10 combinations (Supplementary Figures 4–6). The standard addition method has been applied because that the alternative and easier “standard series method,” which is described in numerous publications, would not provide correct analytical results. The difficulty to obtain reliable assaying results with the standard series method was due to the different matrix effects of root and leaf extracts obtained from each of the 10 combinations tested in Experiment 1. The effect could be eliminated through the use of the standard addition method, which allowed us to obtain correct analytic results.

### Determination of Salicylic Acid, Benzoic Acid, Iodosalicylates, Iodobenzoates, and Plant-Derived Thyroid Hormone Analogs

Roots of plants from the hydroponic system (Experiment 1) and lettuce leaves in all three experiments were tested using LC-MS/MS to measure the content of SA, BeA (benzoic acid), 5-ISA, 3,5-diISA, 2-iodobenzoic acid (2-IBeA), 4-iodobenzoic acid (4-IBeA), 2,3,5-triiodobenzoic acid (2,3,5-triIBeA), iodotyrosine (I-Tyr), sodium salt triiodothyronine (T3-Na), triiodothyronine (T3), and thyroxine (T4) (Supplementary Figure 7). The root and leaf content of these compounds was analyzed in extracts prepared with 75% ethanol containing 50 ng⋅mL^–1^ of deuterated salicylic acid (SA-d4, Sigma-Aldrich). Sample extraction and filtration procedures were the same as described in our previous research (Smoleń et al., 2020).

The compounds were also measured in **RootSec** after lettuce harvesting. The **RootSec** were mixed with 20 mM Tris HCl buffer (pH 8.5). The collection and storage of **RootSec** is described in section “Plant Material and Treatments.”

Root secretions stored in the TRIS-HCl buffer were mixed in vortex prior to analysis and centrifuged at 5°C for 15 min at 4,500 rpm. Then, the supernatant was filtered with a 0.22 μm nylon syringe filters (FilterBio NY Syringe Filter, Phenomenex, Torrance, CA, United States) and analyzed using LC-MS/MS according to Smoleń et al. (2020).

### Biofortification Target and the Safety of Iodine-Enriched Lettuce Consumers

The results of I measurements in lettuce leaves were used in each of the experiments to calculate the following coefficients: (1) recommended daily allowance of iodine (% RDA-I) and (2) hazard quotient for iodine (HQ-iodine). They were calculated for 100 g of fresh lettuce leaves, considering the daily I requirement of adults of 150 μg. The % RDA-I and HQ-iodine coefficients were calculated using mathematical formulas described in detail by Smoleń et al. (2019a).

### Gene Expression Analysis

Plants cultivated in the hydroponic NFT system (Experiment 1) were used as material for gene expression analysis. Leaves and roots for RNA extraction were collected directly before harvest. The leaf and root samples were collected from 8 plants (2 plants from each of the 4 replications), separately for each of the 10 treatments. The third youngest leaf and root samples (portions of 5–10 cm, with tips) were collected for each plant. The samples were immediately frozen in liquid nitrogen and stored at −80°C until isolation of RNA. Total RNA extraction was carried out with a Direct-zol^TM^ RNA MiniPrep Plus RNA isolation kit (Zymo Research, Irvine, CA, United States), according to the manufacturer’s instructions. RNA samples were treated with 1 U μl^–1^ RNase-free Dnase I (Ambion Inc., Austin, TX, United States) and 40 U μl^–1^ RiboLock RNase Inhibitor (Thermo Fisher Scientific, Wilmington, DE, United States) to avoid contamination by DNA and RNA degradation. The quality and integrity of RNA samples were verified by electrophoresis in 1% agarose gel in denaturing conditions. The concentration and quality of RNA were evaluated spectrophotometrically using NanoDrop 2000c (Thermo Fisher Scientific, Wilmington, DE, United States) at 230, 260, and 280 nm. cDNA synthesis was conducted in four biological replicates, each comprising two plants. One microgram of RNA from each sample was transcribed into cDNA using the iScript cDNA synthesis kit (BioRad laboratories, Hemel Hempstead, United Kingdom), according to the manufacturer’s instructions. The cDNA was frozen at −20°C until it was used as a template in real-time qPCR using the StepOnePlus^TM^ Real-Time PCR System (Applied Biosystems, Foster City, CA, United States), according to the following steps: denaturation at 95°C for 10 min; 40 cycles at 95°C for 15 s, 60°C for 30 s, and 72°C for 30 s. The melting curves were obtained by melting the amplicons from 60 to 95°C for 15 s; the temperature was increased by 0.3°C per cycle.

For expression analysis, five genes possibly related to selected metabolic pathways for I and/or iodosalicylates in lettuce, i.e., *per12-like*, *per-64-like*, *samdmt*, *cipk6*, and *msams5*, were chosen (Supplementary Table 1). Moreover, differential expression patterns of those genes were shown by RNA-seq in leaves and roots of *L. sativa* ‘‘Melodion’’ of control plants and supplemented with SA, KIO_3_ and KIO_3_+V (data unpublished). Gene-specific primers for real-time qPCR were designed using Primer3Plus^1^ based on *L. sativa* var. *capitata* ‘‘Melodion’’ transcript sequences *de novo* assembled from RNAseq, deposited in the NCBI GeneBank (Acc. No MT649253, MT649254, MT663549-MT663551) and Lettuce Genome Resource^2^ (Supplementary Table 1).

The absence of primer-dimer and hairpin structures was determined using IDT-OligoAnalyzer 3.1^3^. The utility of the designed primers was validated in a reverse-transcriptase polymerase chain reaction (RT-PCR) and confirmed by electrophoresis in 1% agarose gel (Supplementary Figure 8). Primer specificity was verified by observing single peaks in all melting curves. The total volume of the reaction mixture was 25 μL. The mixture included 12.5 μL Maxima SYBR Green/ROX qPCR Master Mix (2X) (Thermo Fisher Scientific), 0.7 μM of (5 μM) each primer (forward and reverse), 2 μL of a 5-fold diluted template cDNA, and a total volume of 25 μL made up with nuclease-free DEPC-treated water (diethylpyrocarbonate; Thermo Fisher Scientific, Wilmington, DE, United States). qPCR reactions were conducted in four biological and three technical replicates. No template controls were included. Amplification efficiencies for all primer pairs were evaluated using serial 10-fold dilutions of pooled cDNA. The efficiency of each primer pair was calculated from the slope of the standard curve using the formula E = 10^– 1/slope^ and converted into percentage values according to the following formula: %E = (E − 1) × 100%.

Actin (*act*) (Smoleń et al., 2016) and protein phosphatase 2A regulatory subunit A3 (*pp2aa3*) (Sgamma et al., 2016) were used as endogenous reference genes. As *Pp2aa3* expression was more stable than that of *act* (Supplementary Figure 9), relative quantification of gene expression was calculated using the 2^–Δ^ ^Δ^ ^C(T)^ method (Livak and Schmittgen, 2001) with Ct value normalization to *pp2aa3*. In the case of *per64-like*, *per12-like*, *cipk6*, and *msams5*, the relative gene expression was compared with control samples from roots, whereas in *samdmt*, it was compared to control samples from leaves, since expression of *samdmt* in root control samples was not detectable (Supplementary Figure 8).

### Statistical Analyses

All data were statistically verified using one-way analysis of variance (ANOVA) in the Statistica 12.0 PL (StatSoft Inc., Tulsa, OK 74104, United States)^4^ program at a significance level of *p* < 0.05. In the case of significant effects, homogenous groups were distinguished on the basis of a *post-hoc* Tukey HSD test. The results obtained were verified statistically by one-way ANOVA and *post-hoc* Tukey HSD test, separately for each of the three experiments and separately for leaves and roots of lettuce in Experiment 1.

## Results

### Plant Biomass

Hydroponic Experiment 1 was the only experiment where the application of 5-ISA and 3,5-diISA (without or with V) caused a reduction in the weight of leaves and whole plants (roots + leaves) compared with the control (Table 3). No negative impact of 3,5-diISA and 3,5-diISA+V on the weight of roots was observed. A comparable increase in root weight was observed for the application of 5-ISA and 5-ISA+V, compared with the control. In this experiment, combined fertilization with V and KIO_3_, KIO_3_+SA, 5-ISA, and 3,5-diISA had no effect on the weight of roots and heads (leaves) of lettuce, compared with the application of these compounds without V.

**TABLE 3:** Fresh weight of roots, leaves /lettuce head/ and whole plants /roots+leaves/ in hydroponic NFT Experiment No. 1 as well as in lettuce leaves /head/ in pot Experiment Nos. 2 and 3.

Treatments	Hydroponics NFT Experiment No. 1			Peat substrate Experiment No. 2	Mineral soil Experiment No. 3
		
	FW of leaves/lettuce head/(g)	FW of roots from one plant (g)	FW of whole plants /roots+leaves/(g)	FW of leaves/lettuce head/(g)	FW of leaves/lettuce head/(g)
Control	291.4 ± 35.2^b^	31.7 ± 4.5^a^	323.1 ± 39.6^b^	174.1 ± 12.2^a^	186.6 ± 22.8^a^
SA	291.3 ± 29.5^b^	33.1 ± 3.7^ab^	324.4 ± 33.2^b^	169.3 ± 14.4^a^	168.5 ± 16.1^a^
KIO_3_	296.8 ± 34.1^b^	34.6 ± 3.8^ab^	331.4 ± 37.7^b^	169.8 ± 12.2^a^	180.5 ± 23.1^a^
KIO_3_+SA	276.5 ± 42.2^b^	32.6 ± 3.8^ab^	309.1 ± 45.9^b^	161.3 ± 14.3^a^	176.9 ± 23.6^a^
5-ISA	171.1 ± 35.7^a^	39.4 ± 6.1^bc^	210.5 ± 41.8^a^	167.7 ± 10.5^a^	194.7 ± 18.5^a^
3,5-diISA	147.7 ± 37.8^a^	30.1 ± 5.2^a^	177.8 ± 42.9^a^	171.9 ± 13.9^a^	185.5 ± 20.3^a^
KIO_3_+V	305.9 ± 33.0^b^	35.2 ± 3.2^ab^	341.1 ± 36.1^b^	167.9 ± 10.7^a^	186.5 ± 19.4^a^
KIO_3_+SA+V	305.6 ± 28.0^b^	33.1 ± 4.1^ab^	338.7 ± 32.0^b^	163.7 ± 13.3^a^	171.7 ± 21.9^a^
5-ISA+V	160.7 ± 35.8^a^	42.9 ± 5.8^c^	203.6 ± 41.4^a^	173.7 ± 9.8^a^	172.5 ± 18.8^a^
3,5-diISA+V	152.0 ± 41.1^a^	33.1 ± 6.3^ab^	185.1 ± 47.3^a^	168.3 ± 10.5^a^	180.2 ± 21.6^a^

*Means in the column followed by different letters separately for each experiments differ significantly at P < 0.05 (n = 8).*

None of the combinations of SA, V, and I compounds had a significant impact on the weight of lettuce heads in either of the two pot experiments (Experiments 2 and 3).

### Gene Expression in Roots and Leaves of Plants Cultivated in a Hydroponic System (Experiment 1)

**FIGURE 1:**
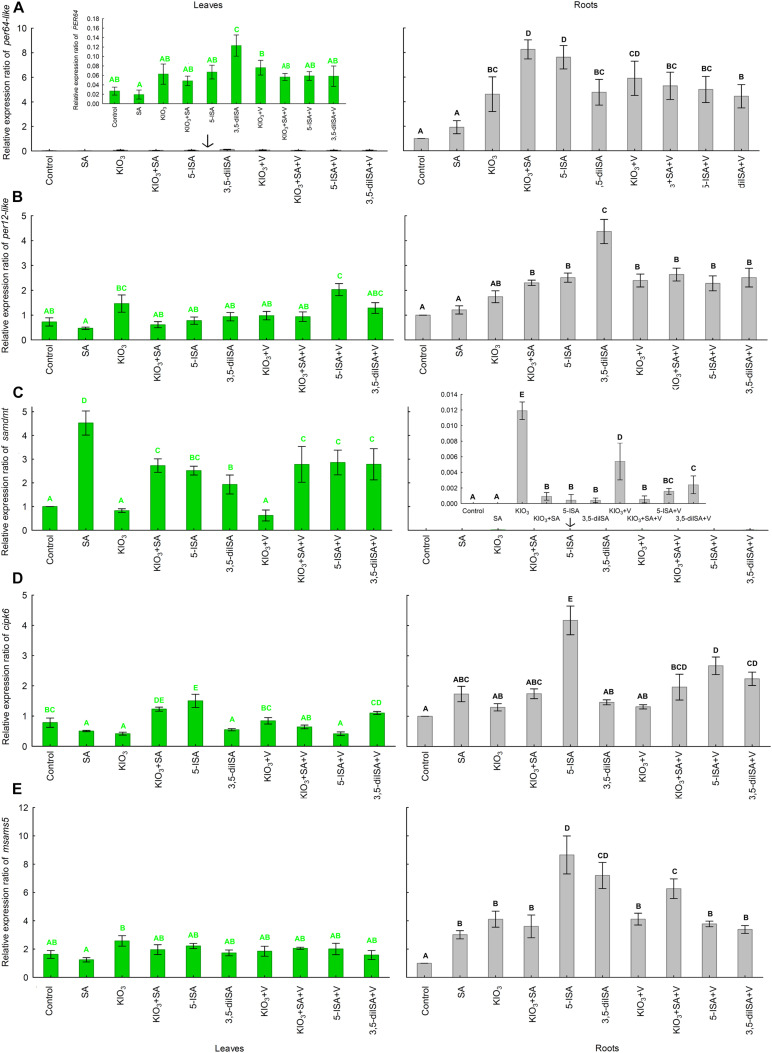
Relative expression of *per64-like* **(A)**, *per12-like* **(B)**, *samdmt* **(C)**, *cipk6* **(D)**, and *msams5* **(E)** genes in leaves and roots of lettuce plants cultivated in hydroponic NFT Experiment No. 1. Means followed by different letters for treatments differ significantly at *P* < 0.05. Bars indicate standard error (*n* = 4).

I compounds, V, and SA applied to the nutrient solution had a statistically significant impact on the expression of the following genes in lettuce roots and leaves: *per64-like*, *per12-like*, *samdmt*, *cipk6*, and *msams5* (Figures 1A–E). Basically, the expression of *per64-like* in leaves and *samdmt* in roots was relatively very low compared with roots and leaves, respectively. As for the remaining three genes (*per12-like*, *cipk6*, and *msams5*), their expression in roots was higher than in leaves.

Compared with the control, all combinations with the application of I, I+SA, and V, caused increased expression of all five genes, i.e., *per64-like*, *per12-like*, *samdmt*, *cipk6*, and *msams5*, in roots (Figures 1A–E). The highest expression level of *per64-like* in roots was observed after application of KIO_3_+SA and 5-ISA. Additionally, exogenous 5-ISA in roots caused the highest expression of *cipk6* and *msams5;* application of 3,5-diISA led to the most pronounced expression of *per12-like*, while plants fertilized with KIO_3_ had the highest expression of *samdmt.* Foliar activity of these five genes in lettuce was completely different that in roots compared with the control. The highest foliar expression of individual genes was as follows: *per64-like* following the application of exogenous 3,5-diISA, *per12-like* following the application of 5-ISA+V, *samdmt* following the application of SA, *cipk6* following the application of exogenous 5-ISA, and *msams5* following the application of KIO_3_.

### Iodine Accumulation and Uptake by Lettuce

**FIGURE 2:**
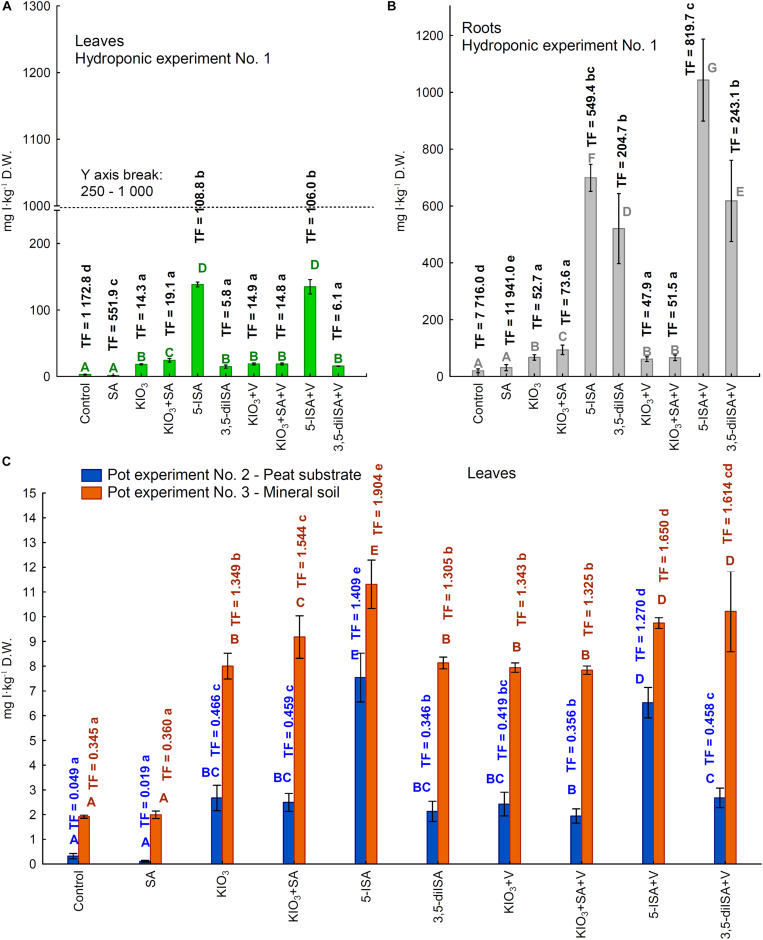
Concentrations of iodine in leaves /heads/ **(A)** and roots of lettuce **(B)** cultivated in hydroponic NFT Experiment No. 1 as well as in leaves of plants cultivated in pot Experiment No. 2 and 3 **(C)**. Transfer factor /TF/ of iodine to roots and leaves /heads/ in the all experiments. Means followed by different letters for treatments, separately for each experiments differ significantly at *P* < 0.05. Color-coded capital letters for iodine content (green and gray for leaves and roots in Experiment 1 and blue and orange for leaves in Experiments 2 and 3); lowercase letters in the same colors for the TF factor in these experiments. Bars indicate standard error (*n* = 8).

Inorganic (IO_3_*^–^*) and organic (5-ISA, 3,5-diISA) I accumulated in larger amounts in roots than in leaves (Figures 2A,B; hydroponic Experiment 1). Root accumulation of I upon the application of both iodosalicylates was higher than upon using KIO_3_ as a fertilizer. Vanadium added to the nutrient solution caused a significant reduction of I content in roots for the combination of KIO_3_+SA+V vs. KIO_3_+SA. Additionally, V caused a significant increase in I content in roots for the combination 5-ISA+V vs. 5-ISA, and for 3,5-diISA+V vs. 3,5-diISA (Figure 2B).

Compared with the control and SA, application of each I compound (without V, with V, and with KIO_3_+SA) caused a significant increase in foliar I content (Figures 2A,C), RDA-I%, and HQ-iodine, as well as I uptake by a single lettuce head (leaves from one plant) in all three Experiments (Supplementary Table 2).

The highest foliar I content, RDA-I%, and HQ-iodine, as well as I uptake by a single lettuce head, was found in plants with applied 5-ISA in all three experiments (Figures 2A,C and Supplementary Table 2). Adding V to the compound (5-ISA+V vs. 5-ISA) reduced foliar I content in both pot experiments (Experiments 2 and 3) but not in the hydroponic system (Experiment 1). Consequently, RDA-I%, HQ-iodine, and I uptake by a single lettuce head was significantly lowered for 5-ISA+V vs. 5-ISA in both pot experiments (Supplementary Tables 2, 3).

Combined fertilization with KIO_3_+V had no impact on leaf I content in any of the three experiments, compared with KIO_3_ without V. As for the application of 3,5-diISA+V (compared with 3,5-diISA without V), only Experiment 3 with a peat substrate showed a significant increase in leaf I content, RDA-I%, HQ-iodine, and I uptake by a single lettuce head (Supplementary Tables 2, 3).

In all three experiments, the calculated TFs for I uptake were significantly modified by application of the I compounds tested, as well as SA and V (Figures 2A–C). The TF values reflected I uptake by plants (I content in roots or leaves) depending on its availability in the rhizosphere, i.e., in the nutrient solution in the hydroponic system (Experiment 1) or mineral soil or peat substrate (Experiments 2 and 3).

### Vanadium Uptake and Accumulation by Lettuce

**FIGURE 3:**
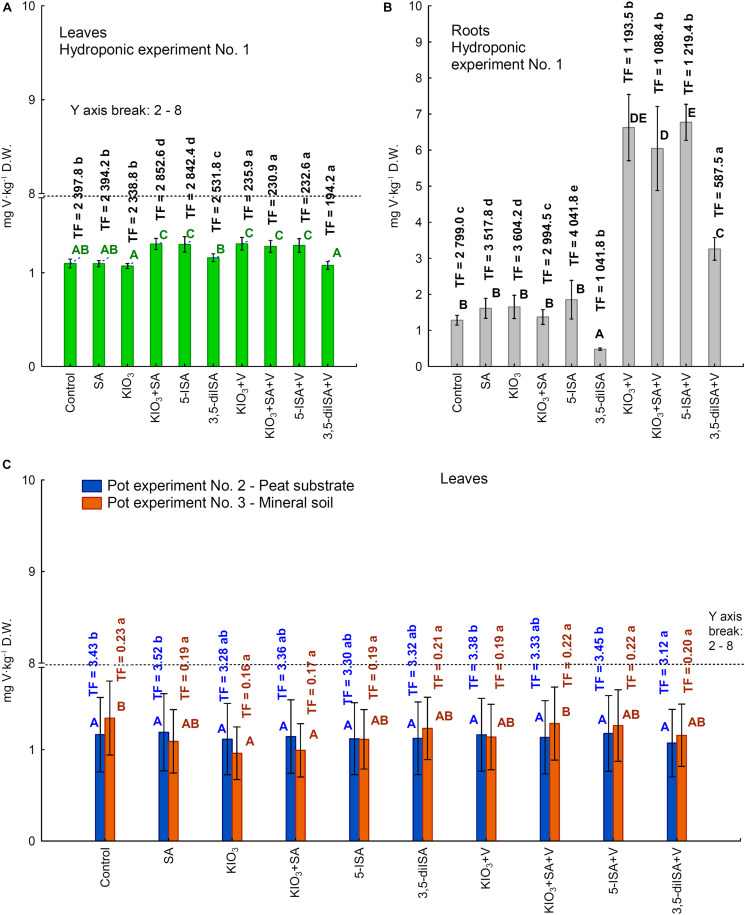
Concentrations of vanadium in leaves /heads/**(A)** and roots of lettuce **(B)** cultivated in hydroponic NFT Experiment No. 1 as well as in leaves of plants cultivated in pot Experiment No. 2 and 3 **(C)**. Transfer factor /TF/ of vanadium to roots and leaves /heads/ in the all experiments. Means followed by different letters for treatments, separately for each experiments differ significantly at *P* < 0.05. Color-coded capital letters for vanadium content (green and gray for leaves and roots in Experiment 1 and blue and orange for leaves in Experiments 2 and 3); lowercase letters in the same colors for the TF factor in these experiments. Bars indicate standard error (*n* = 8).

In hydroponic Experiment 1, V was accumulated in larger amounts in roots than in leaves (Figures 3A,B). Fertilization with V combined with KIO_3_, KIO_3_+SA, and 5-ISA caused an approximately 4-fold increase in V content in roots compared with the control (Figure 3B). Following fertilization with 3,5-diISA without applying ammonium metavanadate, the content of V in roots was lower than in the control (Figure 3B); V uptake by roots of a single plant and by whole plants (roots + head) was consequently lower (Supplementary Table 3). Application of 3,5-diISA+V resulted in a significant increase in V content in roots and V uptake by roots of a single plant and by whole plants (roots + head) compared with 3,5-diISA without V. However, the increase in root V content and in V uptake by roots and whole plants was less effective for fertilization with 3,5-diISA+V than for KIO_3_+V, KIO_3_+SA+V, and 5-ISA+V than for 3,5-diISA, KIO_3_, KIO_3_+SA, and 5-ISA, respectively. This was confirmed by TF values in roots for the respective combinations with and without V fertilization.

Following the application of KIO_3_+V, KIO_3_+SA+V, and 5-ISA+V, the foliar content of V for hydroponic Experiment 1 was significantly higher than that for the control (Figure 3A); however, it was still at the same level as following the application of KIO_3_+SA and 5-ISA without ammonium metavanadate. Additionally, in Experiment 1, the combination of KIO_3_+V was the only one for which V uptake by a single head in Experiment 1 was significantly higher than that for KIO_3_ without ammonium metavanadate (Supplementary Table 3).

In the two pot experiments with lettuce, foliar V content (Figure 3C) and V uptake by a single head (leaves from one plant) (Supplementary Table 3) were significantly lower than in the control but only for the KIO_3_ and KIO_3_+SA combination in Experiment 3. For the remaining combinations, the content of V and V uptake by a single head was the same as in the control in both pot experiments (Figure 3C and Supplementary Table 3).

In Experiment 3, the TF for foliar V was about 20-fold lower than in Experiment 2 (Figure 3C), which was due to lower V content in the peat substrate than in mineral soil (Table 2).

### vHPO Activity in Lettuce

**FIGURE 4:**
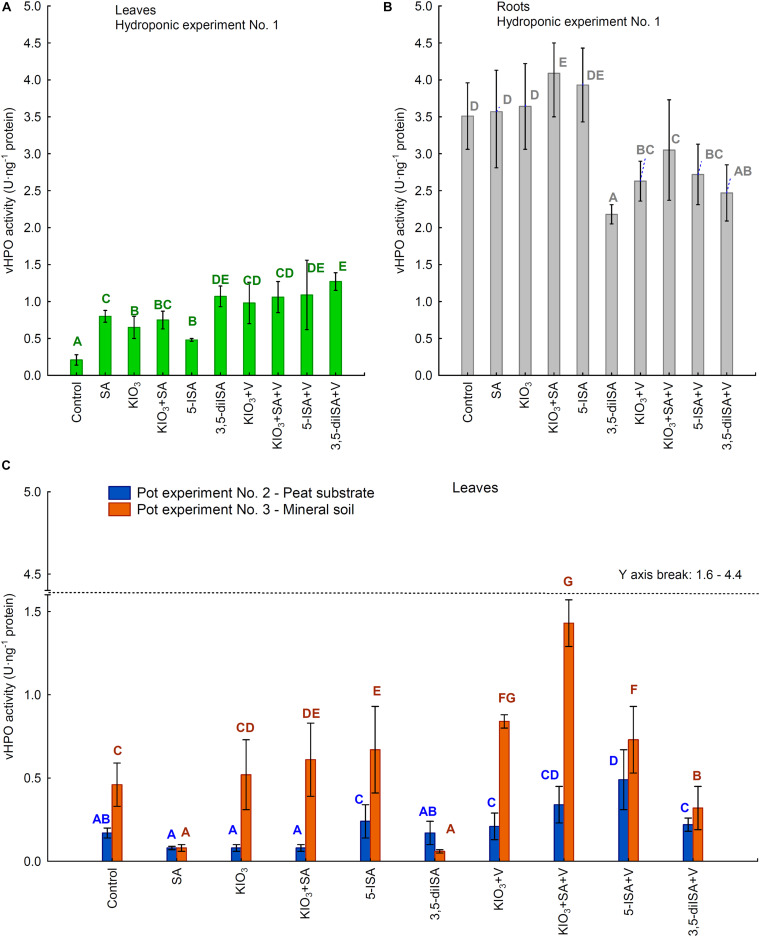
Activity of vanadium-dependent haloperoxidase (vHPO) in leaves /heads/**(A)** and roots **(B)** of lettuce cultivated in hydroponic NFT Experiment No. 1 as well as in leaves of plants cultivated in pot Experiment No. 2 and 3 **(C)**. Means followed by different letters for treatments, separately for each experiments differ significantly at *P* < 0.05. Color-coded capital letters for vHPO activity—green and gray for leaves and roots in Experiment 1 and blue and orange for leaves in Experiments 2 and 3. Bars indicate standard error (*n* = 8).

The activity of vHPO in roots (Experiment 1) and leaves of lettuce in all three experiments was significantly modified by the application of the compounds studied to the nutrient solution or substrate in the pots (Figures 4A–C). In hydroponic Experiment 1, the vHPO activity measured in roots was 2.5- to 4.0-fold higher than that in leaves (Figures 4A,B). Foliar activity of vHPO in all three experiments was in the range between 0.08 and 1.43 U⋅ng^–1^ protein.

The root activity of vHPO following the application of KIO_3_+SA and 5-ISA (Figure 4B) in the hydroponic system was significantly higher than that in the control. These two combinations also produced a significantly higher foliar activity of vHPO than that in the control (Figure 4A). For all four combinations with ammonium metavanadate fertilization and 3,5-diISA (without V), the root activity of vHPO was lower than in the control, while foliar activity of vHPO was significantly higher than that in the control (Figures 4A,B). Furthermore, 3,5-diISA (vs. 3,5-diISA+V) was the only combination where the activity of vHPO in both roots and leaves was the same. Additional fertilization with V together with KIO_3_, KIO_3_+SA, and 5-ISA caused a significant increase in vHPO in leaves and a decrease in the roots compared with the application of these compounds without V.

In each of the three experiments, the highest vHPO activity was measured in leaves of plants representing different combinations: 3,5-diISA+V in Experiment 1 (1.27 U⋅ng^–1^ protein), 5-ISA+V in Experiment 2 (0.49 U⋅ng^–1^ protein), and 5-ISA+V in Experiment 3 (1.43 U⋅ng^–1^ protein) (Figures 4A,C). The lowest foliar activity of vHPO was identified in the control in Experiment 1 (0.21 U⋅ng^–1^ protein) and for SA in Experiments 2 and 3 (0.08 U⋅ng^–1^ protein in each experiment).

### BeA, SA, and Iodine Metabolites in Secretions Collected as a Result of Root Pressure

The contents of I^–^, IO_3_*^–^*, BeA, SA, 5-ISA, 2-IBeA, 4-IBeA, 2,3,5-triIBeA, I-Tyr, T3-Na, T3, and T4 measured in root secretions collected as a result of root pressure (**RootSec**) from plants in Experiment 1 differed significantly between the combinations studied (Table 4).

**TABLE 4:** Results of the determination of iodides, iodates, organic acids, and iodine metabolites in secretions collected as a result of root pressure (RootSec)—this is in white secretion on the surface of the root neck after cutting the heads (lettuce leaves).

Treatments	(μg⋅dm^–^^3^)*						
	Iodides (I^–^)	Iodates (IO_3_*^–^*)		BeA		SA	
Control	16.1 ± 0.25a	1.08 ± 0.07ab		68 ± 9.0b		638.6 ± 10.0b	
SA	10.4 ± 0.37a	0.74 ± 0.15ab		98 ± 0.6cd		214.6 ± 18.3a	
KIO_3_	184.3 ± 3.69b	2.02 ± 0.05c		116 ± 9.9cde		2 163.0 ± 100.0c	
KIO_3_+SA	215.2 ± 19.76bc	2.94 ± 0.71d		88 ± 10.0bcd		118.2 ± 18.9a	
5-ISA	1 789.9 ± 18.29e	0.70 ± 0.11ab		2 ± 1.0a		740.6 ± 4.0b	
3,5-diISA	291.2 ± 23.36c	0.67 ± 0.02ab		128 ± 10.0de		258.4 ± 51.1a	
KIO_3_+V	203.5 ± 3.34b	0.28 ± 0.07a		10 ± 1.4a		106.6 ± 5.4a	
KIO_3_+SA+V	169.8 ± 15.74b	0.65 ± 0.30ab		74 ± 9.8bc		104.3 ± 1.3a	
5-ISA+V	389.6 ± 70.51d	1.54 ± 0.06bc		132 ± 10.0de		254.0 ± 82.9a	
3,5-diISA+V	171.2 ± 10.99b	2.13 ± 0.32cd		138 ± 10.0e		221.3 ± 49.3a	

	**5-ISA**	**3,5-diISA**		**2-IBeA**		**4-IBeA**	

Control	8.4 ± 0.82a	<LOQ**		0.05 ± 0.01abc		0.04 ± 0.01a	
SA	8.3 ± 0.57a	<LOQ		0.15 ± 0.01d		0.19 ± 0.01c	
KIO_3_	8.7 ± 0.73a	<LOQ		0.07 ± 0.01abc		0.10 ± 0.01abc	
KIO_3_+SA	7.2 ± 0.24a	<LOQ		0.26 ± 0.03e		0.08 ± 0.01ab	
5-ISA	386.1 ± 11.45d	<LOQ		0.02 ± 0.01ab		0.06 ± 0.01ab	
3,5-diISA	58.6 ± 0.67b	63.9 ± 6.07a		0.01 ± 0.00a		0.06 ± 0.01ab	
KIO_3_+V	7.4 ± 0.36a	<LOQ		0.08 ± 0.01bc		0.11 ± 0.03abc	
KIO_3_+SA+V	7.7 ± 1.10a	<LOQ		0.06 ± 0.01abc		0.16 ± 0.02bc	
5-ISA+V	215.7 ± 97.09c	<LOQ		0.09 ± 0.02cd		0.08 ± 0.01ab	
3,5-diISA+V	46.2 ± 1.48b	83.6 ± 3.64b		0.10 ± 0.01cd		0.36 ± 0.05d	

	**2,3,5-triIBeA**	**I-Tyr iodotyrosine**	**T3-Na**		**T3**		**T4**

Control	0.25 ± 0.01a	0.52 ± 0.08bc	484.1 ± 40.5ab		2.81 ± 0.10d		0.22 ± 0.01ab
SA	2.67 ± 0.46b	0.56 ± 0.02bc	422.4 ± 41.6ab		1.31 ± 0.01b		1.83 ± 0.10cd
KIO_3_	0.45 ± 0.10a	0.55 ± 0.06bc	746.7 ± 58.6c		2.84 ± 0.10d		0.01 ± 0.00a
KIO_3_+SA	0.17 ± 0.07a	0.71 ± 0.06c	608.9 ± 77.5bc		3.46 ± 0.10e		0.64 ± 0.09b
5-ISA	0.01 ± 0.002a	0.52 ± 0.24bc	342.0 ± 3.4ab		5.00 ± 0.06f		4.78 ± 0.10h
3,5-diISA	0.09 ± 0.01a	0.16 ± 0.01ab	285.2 ± 36.1a		2.10 ± 0.10c		4.04 ± 0.15g
KIO_3_+V	0.08 ± 0.02a	2.17 ± 0.10d	270.6 ± 60.5a		0.16 ± 0.01a		2.34 ± 0.21de
KIO_3_+SA+V	0.08 ± 0.01a	2.26 ± 0.01d	409.7 ± 1.6ab		1.34 ± 0.10b		0.15 ± 0.01ab
5-ISA+V	0.17 ± 0.02a	0.50 ± 0.08bc	225.3 ± 2.0a		3.83 ± 0.10e		1.63 ± 0.10c
3,5-diISA+V	0.13 ± 0.02a	0.01 ± 0.00a	591.3 ± 109.0bc		1.16 ± 0.01b		3.14 ± 0.10f

*The content of the following compounds was analyzed: iodides (I^–^), iodates (IO_3_^–^), benzoic acid (BeA), salicylic acid (SA), 5-iodosalicylic acid (5-ISA), 3,5-diiodosalicylic acid (3,5-diISA), 2-iodobenzoic acid (2-IBeA), 4-iodobenzoic acid (4-IBeA), 2,3,5-triiodobenzoic acid (2,3,5-triIBeA), iodotyrosine (I-Tyr), sodium salt triiodothyronine (T3-Na), triiodothyronine (T3), and thyroxine (T4).**Limit of quantification (LOQ) for 3,5-diISA was 2.748.

Compared with the control and SA application in all remaining combinations, I content is RootSec was markedly higher. The highest content of I^–^ was found after the application of 5-ISA (without V); it was 9.7-fold higher than when KIO_3_ was applied to the nutrient solution alone (Table 4). Compared with the control, a significant increase of IO_3_*^–^* was observed in RootSec following the application of KIO_3_, KIO_3_+SA, 5-ISA+V, and 3,5-diISA+V, with the highest RootSec content of IO_3_*^–^* being observed for fertilization with KIO_3_+SA.

3,5-diISA was detected in RootSec exclusively after the application of 3,5-diISA (with or without V). Additionally, the RootSec content of 5-ISA was on average 6.2-fold higher for these two combinations than for the control. However, the highest content of 5-ISA in RootSec (25- to 46-fold higher than in the control) was demonstrated following its application to the nutrient solution. Plants treated with exogenous 5-ISA were characterized by the highest content of T3 and T4 and the lowest content of BeA and 2,3,5-triIBeA in RootSec. The RootSec content of T4 was equally high for fertilization using 3,5-diISA.

For all combinations with fertilization using ammonium metavanadate, application of the compound caused a reduction in T3 content in RootSec; the comparison encompassed combinations with KIO_3_, KIO_3_+SA, 3,5-diISA, 5-ISA without and with V (Table 4). The application of 5-ISA+V vs. 5-ISA (without V) had a reduced content of SA, 5-ISA, T3, and T4 and increased 2-IBeA in RootSec. When it comes to fertilization using 3,5-diISA+V vs. 3,5-diISA (without V), it caused a significant increase in BeA, 2-IBeA, and 4-IBeA and a decrease in the content of 5-ISA, T3, and T4 in RootSec.

Application of exogenous SA to the nutrient solution caused a significant increase in the RootSec content of 2,3,5-triIBeA compared with all the remaining combinations (Table 4).

### Determination of Iodides (I*^–^*) and Iodates (IO_3_*^–^*) in Roots and Leaves of Lettuce in a Hydroponic System (Experiment 1)

Roots and leaves had a higher content of iodides (I^–^) than iodates (IO_3_*^–^*), from 12.5 times in roots for SA treatment to 53,750 times in leaves for 5-ISA+V treatment (Table 5). There were trace amounts of IO_3_*^–^* in leaves and roots in all combinations subjected to analysis. Even in roots and leaves of plants fertilized with KIO_3_, the content of IO_3_*^–^* was lower than or similar to the control.

**TABLE 5:** Concentrations of iodides (I^–^) and iodates (IO_3_*^–^*) in roots and leaves of lettuce in hydroponics NFT Experiment No. 1 (speciation of iodine analyzed HPLC-ICP-MS/MS) as well percentage of the iodides and iodates in relation to the total iodine.

Experiment No. 1. Hydroponic NFT/Part of plant	Treatments	(mg⋅kg^–^^1^ D.W.)			Percentage of the sum of iodides and iodates in relation to the total I content* (%)	Percentage of the sum of analyzed organic-I** in relation to the total I content* (%)	Theoretical percentage share of the content of other unanalyzed organic or inorganic I compounds in the total I content (%)
			
		Iodides (I^–^)	Iodates (IO_3_*^–^*)	The sum of two forms of I: I^–^ and IO_3_*^–^*			
Leaves	Control	2.34 ± 0.005b	0.0272 ± 0.0008d	2.365 ± 0.005b	57.7 ± 0.80c	3.26 ± 0.11d	39.0
	SA	0.97 ± 0.006a	0.0293 ± 0.0009d	0.998 ± 0.006a	56.3 ± 0.53c	2.39 ± 0.26c	41.4
	KIO_3_	15.06 ± 0.042f	0.0146 ± 0.0006b	15.078 ± 0.042f	73.4 ± 0.64f	0.71 ± 0.05b	25.9
	KIO_3_+SA	17.49 ± 0.140g	0.0139 ± 0.0001b	17.504 ± 0.140g	56.0 ± 0.33c	0.16 ± 0.02a	43.9
	5-ISA	97.06 ± 0.189i	0.0488 ± 0.0006f	97.113 ± 0.189i	67.6 ± 1.77e	0.25 ± 0.01ab	32.1
	3,5-diISA	8.63 ± 0.044d	0.0002 ± 0.0001a	8.625 ± 0.044d	5.3 ± 0.60a	0.75 ± 0.01b	93.9
	KIO_3_+V	14.12 ± 0.041e	0.0215 ± 0.0004c	14.143 ± 0.041e	63.1 ± 0.36d	0.30 ± 0.06ab	36.6
	KIO_3_+SA+V	18.92 ± 0.229h	0.0023 ± 0.0001a	18.918 ± 0.229h	84.1 ± 1.20h	0.66 ± 0.01ab	15.3
	5-ISA+V	118.25 ± 0.623j	0.0022 ± 0.0001a	118.252 ± 0.624j	72.4 ± 0.12f	0.31 ± 0.01ab	27.3
	3,5-diISA+V	3.46 ± 0.009c	0.0428 ± 0.0017e	3.507 ± 0.010c	23.6 ± 0.20b	7.01 ± 0.12f	69.4
Roots	Control	7.70 ± 0.0187b	0.8780 ± 0.0007e	8.577 ± 0.019b	22.7 ± 0.32g	2.41 ± 0.04	74.9
	SA	0.16 ± 0.0025a	0.0137 ± 0.0001a	0.169 ± 0.002a	0.3 ± 0.01a	0.70 ± 0.02	99.0
	KIO_3_	10.87 ± 0.0131c	0.9914 ± 0.0006ef	11.866 ± 0.013c	12.8 ± 0.12d	0.55 ± 0.05	86.7
	KIO_3_+SA	34.99 ± 0.4050e	0.2436 ± 0.0037d	35.235 ± 0.408e	25.6 ± 0.24h	0.40 ± 0.04	74.0
	5-ISA	72.58 ± 0.0764f	1.7800 ± 0.0428g	80.364 ± 0.119f	14.0 ± 0.23e	0.86 ± 0.03	85.1
	3,5-diISA	8.12 ± 0.0183b	0.9589 ± 0.0005f	9.080 ± 0.018b	1.1 ± 0.01ab	9.18 ± 0.10	89.8
	KIO_3_+V	13.53 ± 0.0426d	0.0531 ± 0.0004ab	13.583 ± 0.042d	16.4 ± 0.15f	1.32 ± 0.06	82.3
	KIO_3_+SA+V	11.27 ± 0.0564c	0.1136 ± 0.0009c	11.382 ± 0.057c	12.5 ± 0.23d	2.10 ± 0.06	85.4
	5-ISA+V	119.26 ± 1.6836g	0.9511 ± 0.0266ef	120.212 ± 1.710g	6.2 ± 0.08c	0.44 ± 0.00	93.4
	3,5-diISA+V	11.59 ± 0.0606c	0.0695 ± 0.0003bc	11.661 ± 0.060c	1.2 ± 0.01b	7.99 ± 0.14	90.8

*Total I content determinate after TMAH extraction (see Figure 1).**Organic-I—I in all organic I compounds analyzed in roots and leaves of lettuce from hydroponic Experiment No. 1 (see Supplementary Table 4, 5). Sum content of I in form: 5-ISA, 3,5-diISA, I-Tyr, T3-Na, T3, 2-IBeA, 4-IBeA, 2,3,5-triIBeA in relation to the total I content. Means in the column followed by different letters separately for each experiments differ significantly at P < 0.05.

The lowest root and leaf content of I^–^ (lower than in the control) was detected in plants treated with exogenous SA (Table 5). The highest content of I^–^ in roots and leaves was found in plants treated with 5-ISA+V; it was significantly higher than in combinations where 5-ISA was used alone.

A comparison of combinations with I applied to the nutrient solution shows the following quantitative root content of I^–^ (Table 5): 5-ISA+V > 5-ISA > KIO_3_+SA > KIO_3_+V > 3,5-diISA+V = KIO_3_+SA+V = KIO_3_ > 3,5-diISA. The quantitative content of I^–^ in leaves was as follows: 5-ISA+V > 5-ISA > KIO_3_+SA+V > KIO_3_+SA > KIO_3_ > KIO_3_+V > 3,5-diISA > 3,5-diISA+V.

An additional application of V together with KIO_3_, KIO_3_+SA, 5-ISA, and 3,5-diISA in each of these four combinations had a different impact on the I^–^ content in leaves and roots (Table 5) and resulted in an increase or decrease in I^–^ content in leaves and roots, compared with application of exogenous I compounds without V.

The percentage of the sum of iodides (I^–^) and iodates (IO_3_*^–^*) in relation to total I content was within the range from 0.3 for SA to 25.6 for KIO_3_+SA in roots, and from 5.3 for 3,5-diISA to 84.1 for KIO_3_+SA+V in leaves (Table 5).

In the supporting information files were included the results of content of BeA, SA, iodine metabolites in roots and leaves (Supplementary Tables 4, 5) as well as content of I and V in soil after lettuce cultivation (Supplementary Table 6).

## Discussion

The aim of research on I biofortification of plants is to establish biofortification regimens, define the threshold of I toxicity for plants, and optimize I biofortification of plants to make it safe and adequate to consumers’ needs (Lawson et al., 2016). Research on I biofortification must be connected with research targeted at expanding knowledge on biochemical, physiologic, and molecular aspects of the functions of trace elements in plants (White and Broadley, 2009).

### Plant Biomass and Iodine Biofortification Efficiency Depending on the Chemical Form of Iodine, Vanadium Application, and Type of Cultivation

The dose of I in the hydroponic system was 37.5-fold higher than that in both pot experiments. In consequence, the I compounds (particularly as 5-ISA and secondarily as 3,5-diISA) were applied at a concentration that was too high, negatively impacting plants (reduced yield) in hydroponic Experiment 1. Smoleń et al. (2017) showed that in the hydroponic system, 5-ISA was toxic to lettuce when applied at a dose of 40.0 μM I; the symptoms were not reported after application of 5-ISA at a dose of 1.6 and 8.0 μM I. The authors did not conduct research on 3,5-diISA. Based on the results of a study by Smoleń et al. (2017) and the reduced lettuce biomass shown in this study (Experiment 1), we presume that the threshold of transition from harmful to toxic I activity in lettuce is somewhere between the dose of 8.0 μM and 10.0 μM of I applied as 5-ISA. Because only one dose of 3,5-diISA was tested, it was impossible to establish an exact threshold of harmfulness/toxicity of exogenous 3,5-diISA on lettuce, as was done for 5-ISA.

Welch and Huffman (1973) did not report a significant impact of 1 μM V (as NH_4_VO_3_) on the yield of lettuce or tomato compared with the control without V fertilization, for doses ranging from 0.05 to 0.40 μM V. In the three experiments described in the publication, simultaneous fertilization with KIO_3_, 5-ISA, and 3,5-diISA plus V at a dose of 0.1 μM V (vs. no V) had no negative impact on the biomass of lettuce.

The highest efficacy of I biofortification on lettuce following the application of 5-ISA (in each of the three experiments) compared with 3,5-diISA and KIO_3_ was justified in research by Smoleń et al. (2017). The authors demonstrated that 5-ISA at a dose of 8.0 μM I was enough to achieve a similar effect of I biofortification in lettuce leaves, as in the case of using KIO_3_ at a dose of 40.0 μM I. In Experiment 1, the dose of 5-ISA (or, to a lesser extent, 3,5-diISA and KIO_3_) was too high in the context of a need to balance I content in the daily diet of consumers. This is indicated by the RDA for I (%) in a 100 g portion of fresh lettuce leaves > 480% and HQ > 0.66; the compound would be harmful to consumers if HQ exceeded 1.0. The two iodosalicylates mentioned above, as well as 2-IBeA, 4-IBeA, 2,3,5-triIBeA, and I-Tyr, and T3 were naturally synthesized in lettuce, which further confirms the results of previous studies on lettuce (Smoleń et al., 2020). Halka et al. (2019) showed that these organic I compounds were present in tomato fruits and willow bark. The problem with defining a “target range” for I biofortification of lettuce with non-organic I compounds (KI and KIO_3_), as well as the determination of human demand of I and estimations of lettuce consumption by the general population were addressed in a study by Lawson et al. (2016). Vegetables enriched with KI and KIO_3_ have been found to be safe both for humans (Tonacchera et al., 2013) and laboratory rats (Pia̧tkowska et al., 2016). In our studies, exogenous 5-ISA and 3,5-diISA caused a significantly higher accumulation of these compounds in leaves and roots. This information may provoke questions on consumer safety where iodosalicylates are used for I enrichment of plants. There is no direct data on the effect of exogenous iodosalicylates used in vegetable growth on animals or humans. We presume that exogenous iodosalicylates may increase health-promoting effects of domesticated plants. This is because the fertilization of plants using inorganic KI or KIO_3_ compounds increases synthesis and accumulation of organic I metabolites in plants, as confirmed in research on lettuce (Smoleń et al., 2020) and tomato (Halka et al., 2019). Even though organic I metabolites, including T3, are present in marine algae in much higher amounts than in lettuce (Fenical, 1975), marine algae are still consumed by a number of people worldwide (González et al., 2019).

In hydroponic Experiment 1 with lettuce, I enrichment of plants was higher than in both pot experiments. This was probably because I taken up directly from the nutrient solution was more readily available to roots and because the dose of I per plant was higher than in Experiments 2 and 3. The effectiveness of I biofortification in plants is much higher in hydroponic and soilless systems than when soil fertilization is used (Blasco et al., 2008). This is due to high I sorption by soil, a phenomenon that is absent in hydroponic nutrient solutions. Iodine sorption in soil is attributable to the mineral fractions and SOM (Kashparov et al., 2005), especially the humified aromatic ring of organic matter but not fresh organic matter (Schlegel et al., 2006). After SOM, the following compounds also participate in I sorption by mineral soils: hydroxides Fe/Al (Yoshida et al., 1992), Cu(I)-Fe (III)-sulfides and Cu(I)-sulfides (Lefèvre et al., 2003), as well as Cu/Cr and Cu/Al (Pless et al., 2007). Additionally, I desorption by soil is very slow, which inhibits I uptake by roots (Dai et al., 2004). The SOM also contains SA and its derivatives (Hue et al., 1986). Molecular I or its non-organic anions in the soil may react with aromatic rings of compounds included in SOM (Yamada et al., 1996). Endogenous iodosalicylates and iodobenzoates were identified in the soil prior to lettuce cultivation. The peat substrate was richer in BeA, SA, 5-ISA, and 2-IBeA than mineral soil. In both pot experiments, lettuce heads grown in the peat substrate accumulated less I than those grown in mineral soil. The sorption of I anions (IO_3_*^–^*) by organic soils was higher than in mineral soils (Yamaguchi et al., 2005). This has also been confirmed by the results of analyses of the peat substrate and mineral soil after lettuce cultivation. Post-cultivation I content in the peat substrate was on average 1.5-fold higher than in mineral soil. The highest efficacy of I enrichment of lettuce following the application of 5-ISA in the peat substrate and mineral soil may have resulted from the low degradation or conversion of low-molecular-weight organic aromatic I compounds in soil, which then may be taken up by roots.

### Relative Expression of Analyzed Genes vs. Iodine Uptake. Iodine Metabolism in Lettuce

The absence of impact or insignificant increase in V content in leaves following ammonium metavanadate fertilization reported in the three experiments was also confirmed in the literature. When V is applied through a soil fertilizer or nutrient solution, it accumulates in roots and its transport to aboveground parts of the plants is very limited. This was also reported for the cultivation of tomato, Chinese green mustard (Vachirapatama et al., 2011), soybean (Kaplan et al., 1990), rice (Chongkid et al., 2007), and lettuce (Gil et al., 1995). Increased V transfer to the aboveground parts of plants is possible if high concentrations of the element, which are potentially toxic to plants, are used in fertilization (Chongkid et al., 2007; Vachirapatama et al., 2011).

Peroxidases are linked to a number of physiological functions. These include the removal of H_2_O_2_, oxidation of toxic reductants, biosynthesis and degradation of lignin, and participation in many other biochemical processes (additional descriptions in Supplementary Data 1). The ion Ca^2+^ has been described as a cofactor for peroxidase (Pandey et al., 2017). Enzymes from the group of V-dependent haloperoxidases (vHPO) contain the bare metal oxide vanadate, as a prosthetic group (Wever and Hemrika, 2001) (see also Supplementary Data 1). In the presence of H_2_O_2_, they oxidize halides (I, Be, Cl) in the following reaction: H_2_O_2_ + X^–^ + H^+^ → H_2_O + HOX, where X represents Cl^–^, Br^–^, or I^–^ (Wever and Hemrika, 2001; Leblanc et al., 2006). The function of vHPO is well-described for marine algae (Leblanc et al., 2006; Verhaeghe et al., 2008). In marine algae, the vHPO enzyme plays a dual function. It can participate in the process of I uptake into cells and is involved in the process of I excretion from cells to the environment in the form of I_2_ (Leblanc et al., 2006).

An additional application of V with different I compounds and SA had no definitive impact on the expression of *per64-like*, *per12-like*, *samdmt*, *cipk6*, or *msams5* in either roots or leaves. *per64-like* was the only gene whose expression decreased in the roots of plants treated with KIO_3_+SA+V, 5-ISA+V, and 3,5-diISA+V (in nutrient solution) when compared to the application of the same compounds without V. For these very same combinations (with and without V), the root activity of vHPO was reported to decrease. Therefore, the level of expression of the *per64-like* gene was correlated with the activity of vHPO in roots. The decreased activity of vHPO was accompanied by a lower expression of *per64-like* in plants treated with KIO_3_+SA+V, 5-ISA+V, and 3,5-diISA+V. These results are sufficient to assign vHPO-like activity to an enzyme encoded by *per64-*like, rather than the one encoded by *per12-like*. Perhaps peroxidase encoded by *per64-like* may have vHPO-like function (may be a V-dependent enzyme). The results of pairwise alignment of protein sequences of *A. thaliana* PER12 and PER64 with vIPO1 *L. digitata* and vBPO *C. officinalis* and *A. nodosum* showed common regions between them (see also Supplementary Data 1). Further *in-silico* research is needed to this end or research directed at isolating PER64-like enzyme to be able to examine its structure and functionality depending on the application of V.

Colin et al. (2005) proved that the *in-vitro* activity of vHPO isolated from *Laminaria digitata* grew intensively within the range 0–10 mM KI and dropped suddenly when KI > 20 mM was used. In the three experiments conducted as part of our study, exogenous KIO_3_, 5-ISA, and 3,5-diISA (applied without V) had a different effect on root and foliar activity of vHPO. Before its uptake by roots or immediately thereafter, IO_3_*^–^* must be reduced to I^–^ (Kato et al., 2013). Surprisingly, there was no significant difference in vHPO activity in the roots of plants treated with KIO_3_ alone (Experiment 1) compared with the control, despite a simultaneous 2.6-fold increase in the expression of the *per64-like* gene. The results indirectly indicate that other molecular and biochemical mechanisms than vHPO must be involved in the process of root uptake and transport of I^–^ produced following IO_3_*^–^* reduction. These mechanisms are probably responsible for chloride transport. The transport of I^–^ within root cells and to the xylem is analogous to the translocation of Cl^–^ ions and takes the form of symport (H^+^/anion) or antiport (Na:K/Cl) or is effected through I channels that are permeable to Cl^–^/I^–^ (White and Broadley, 2001; Roberts, 2006; Colmenero-Flores et al., 2007).

The three experiments share the observation that none of the tested I compounds silenced foliar activity of vHPO. 3,5-diISA was the only compound that reduced the activity of vHPO in roots, an observation consistent with Smoleń et al. (2020). This may be because of the specific effect of 3,5-diISA on vHPO, which inhibited the activity of the enzyme (already at a dose of 10 μM; or 20 μM I) but did not suppress the expression of the *per64-like* gene, which has been linked with vHPO-like functions. Notably, lower expression of *per64-like* was reported in the roots of plants treated with 3,5-diISA but only compared with KIO_3_+SA and 5-ISA, with a simultaneous increase in gene expression in comparison with the control. 5-ISA increased the foliar activity of vHPO in all three experiments compared with the control, which translated into the highest I accumulation in lettuce leaves. The potential mechanism most likely to stimulate foliar activity of vHPO through 5-ISA and 3,5-diISA is not fully understood. It could be a result of catabolism in both iodosalicylates to I^–^, as only in this form could I be taken up intracellularly with the aid of vHPO in a mechanism resembling the one described for marine algae by Leblanc et al. (2006).

The activity of vHPO and expression of *per64-like* in leaves and roots were determined at the final stage of cultivation (shortly before lettuce harvesting), which means that the measurements were made after the plants have been exposed to exogenous V and I compounds for an extended time. While theoretically it may seem that V fertilization should increase the activity of vHPO in plants, due to the higher availability of the enzyme’s cofactor, lettuce fertilization with V caused decreased activity of vHPO and expression of the *per64-like* gene. Reduction in vHPO activity following test fertilization with I compounds + V is consistent with the previous findings of Smoleń et al. (2020). The authors showed that fertilization with V without the simultaneous use of I (plant cultivation with trace amounts of I in the nutrient solution) significantly enhanced the activity of vHPO. Given these results, the assignment of a vHPO-like function to the protein encoded by *per64-like* seems to be substantively justified.

Fertilization with V did not cause an increase in foliar V content in any of the experiments conducted as part of this research (except for KIO_3_+V vs. KIO_3_ in Experiment 1). However, a considerable increase in root V content following V fertilization was observed. The results were consistent with the literature. V fertilization of Chinese greens, at a dose of 0.39, 0.79, and 1.57 mM, caused a proportional increase in V content in the plants, with the following preserved concentration gradient: roots > stems > leaves system (Vachirapatama et al., 2011). A similar V concentration gradient was obtained by Akoumianaki-Ioannidou et al. (2016) for sweet basil fertilized with NH_4_VO_3_. The rate of transfer of V from roots to leaves can only be increased if it is used at very high doses, which may be harmful to plants. The negative effect of V on plants also depends on its chemical form. Findings for soybean showed that a harmful dose of VOSO_4_ was 1.2 mM V (Kaplan et al., 1990). A dose of 0.39 mM V was reported to be harmful for rice (Chongkid et al., 2007). The adverse effects of V may include root darkening, decreased number of secondary roots, decreased turgor pressure, loss of leaf firmness, and plastid degradation in plants (Gil et al., 1995). In Experiment 1, we did not observe any negative impact of V on the development of roots, which was demonstrated by the biomass obtained from the roots of one of the plants.

### Expression of *cipk6* Gene

**FIGURE 5:**
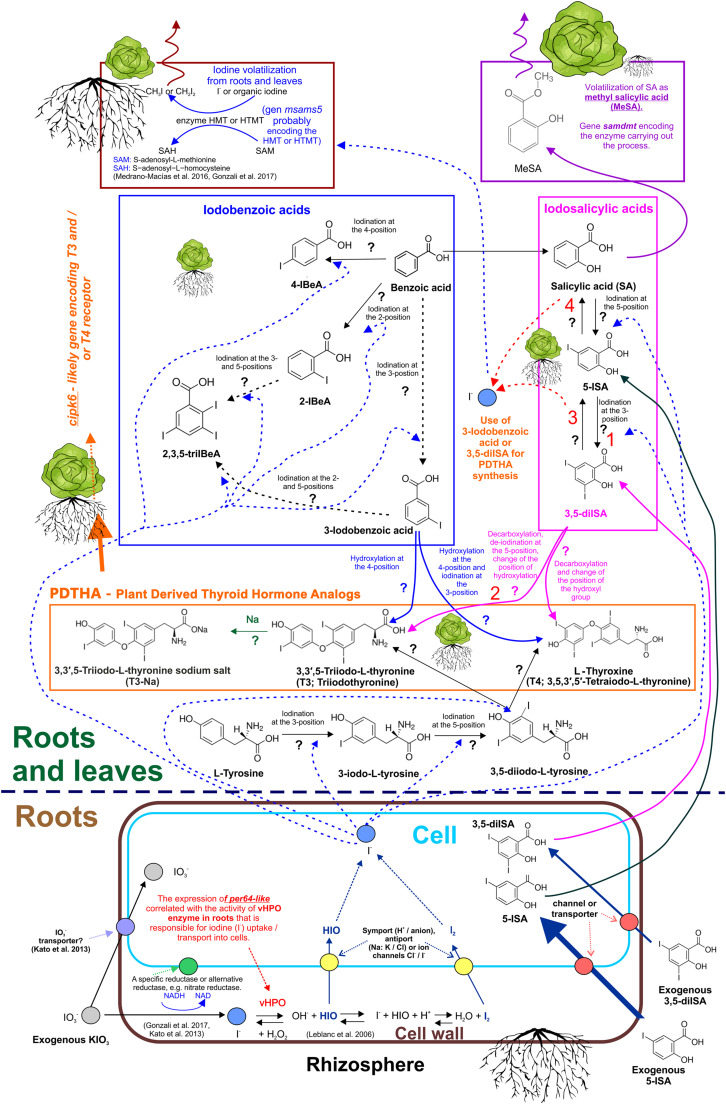
Mechanism of uptake of non-organic and organic iodine compounds. Theoretical metabolic pathway of iodosalicylates, iodobenzoates, and plant-derived thyroid hormone analogs (PDTHA) in lettuce—summary of the study and literature data. 1 and 2—Processes that mainly occur in roots. Foliar activity intensifies after application of 5-ISA and 3,5-diISA. 3—Deiodination of 3,5-diISA. Observed increased content of 5-ISA following exogenous application of 3,5-diISA. 4—Deiodination of 5-diSA. Observed increased content of SA following exogenous application of 5-diSA and 3,5-diISA. **?—**Undefined enzymatic/metabolic processes that carry out these reactions.

**FIGURE 6:**
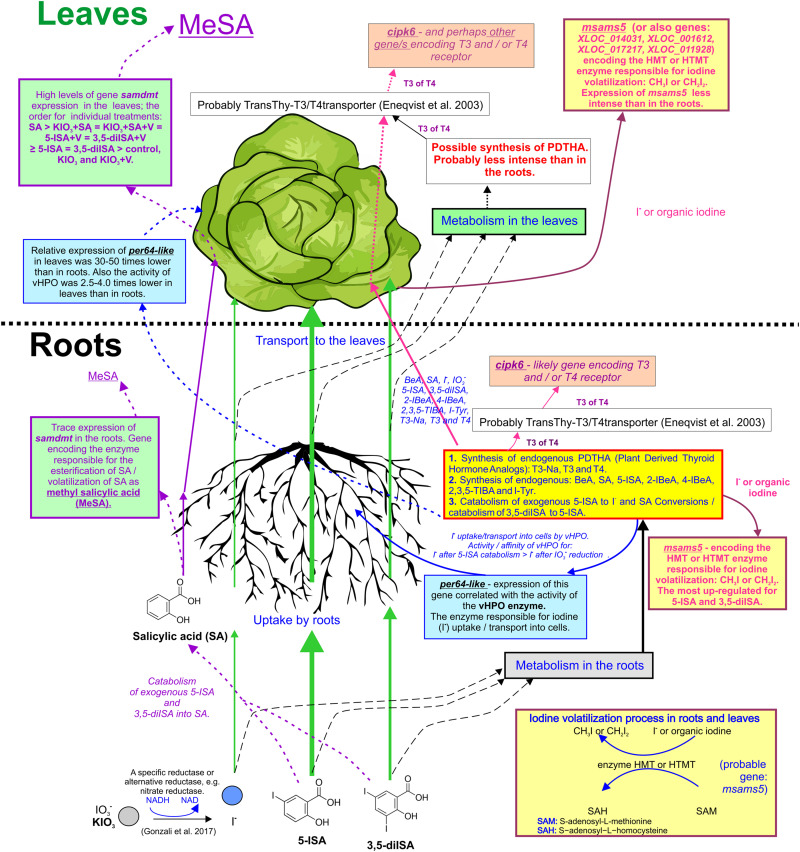
Graphic summary of study outcomes: uptake, transport, and metabolic pathways of iodine compounds and SA. Genes with described activity and function: *Peroxidase 64-like (per64-like)*: the gene’s activity has been linked to the activity/functionality of V-dependent haloperoxidase (vHPO) in roots and leaves of lettuce. *Peroxidase 12-like (per12-like*): no correlation has been found between the gene’s expression and vHPO activity in roots. *S-adenosyl-L-methionine-dependent methyltransferase (samdmt)*: gene that possibly encodes an enzyme that conducts the process of esterification/volatilization of methyl salicylic acid (MeSA). *CBL-interacting serine/threonine-protein kinase 6 (cipk6):* gene that likely encodes triiodothyronine (T3) and/or thyroxine (T4) receptors. *S-adenosylmethionine synthase 5 (msams5):* gene that possibly encodes an enzyme responsible for I volatilization in the process of methylation (synthesis of CH_3_I or CH_2_I_2_).

The available literature describes the likely biochemical mechanisms of PDTHA activity in plants by comparing their function to thyroid hormones (T3 or T4) in humans. Eneqvist et al. (2003) stated that higher plants can produce a protein homologous to human transthyretin, which is responsible for T3 and T4 transport (TransThy-T3/T4trans). Additionally, the TransThy-T3/T4trans protein from higher plants, including *A. thaliana*, tomato, and potato was more closely related in the phylogenetic tree of the transthyretin protein family to the protein found in *Homo sapiens* than to TransThy-T3/T4trans from bacteria or fungi. In model research, Pessoa et al. (2010) showed that exogenous T4 can be bound by transthyretin-like protein in *A. thaliana*. Furthermore, Power et al. (2000) showed that transthyretin in humans belongs to the group of proteins that includes thyroxine-binding globulin and albumin. The chemical bond is responsible for transporting thyroid hormones in blood. Davis et al. (2000) showed that in humans, T4 can activate signal transduction proteins, such as mitogen-activated protein kinase (MAPK). Additionally, T4 can enhance the activity of several nuclear transactivator proteins, through serine phosphorylation by MAPK. A similar complex mechanism may exist in lettuce. Findings show that the protein encoded by *cipk6* can likely perform the function of a T3 and/or T4 receptor (see the functional annotation of the *cipk6* gene in Supplementary Data 2). This assumption is justified by results of chemical analyses of roots and RootSec. The synthesis of T3 and T4 (and possibly other isomers of these PDTHAs) probably occurs, with the participation of iodobenzoates and/or iodosalicylates (5-ISA and 3,5-diISA) as the substrates of PDTHA (Figures 5, 6). The results showed that the process occurs primarily in roots. T3 and T4 are then transported to the aboveground parts of plants (Figures 5, 6). The observation of root synthesis of T3 are consistent with the outcome of our previous study (Smoleń et al., 2020). In the present study, we measured T4 directly in leaves and roots. The key problem with T3 and T4 measurements in plant tissues is that there are no analytical protocols dedicated to the analysis of PDTHA content in plants. However, the content of T4 was measured directly in RootSec. Therefore, it is important that we postulate the need to search for and elaborate on the optimum methods or analytical procedures that would enable measurements of the total T4/T3 and other PDTHAs in the tissue of plants. We believe that the results of our analyses of non-organic and organic I metabolites (particularly T3 and T4) in roots, RootSec, and leaves, provide grounds to assign the function of a T3 and/or T4 receptor in lettuce to a protein encoded by *cipk6*. For *cipk6*, a significant correlation coefficient was reported between its expression vs.: 1) the content of T3 (*r* = 0.30^∗^), 5-ISA (*r* = 0.72^∗^), and total content of I (*r* = 0.84^∗^) directly in roots and 2) the content of T3 (*r* = *0.60*^∗^), T4 (*r* = *0.58*^∗^), 5-ISA (*r* = 0.89^∗^), and total content of I (*r* = 0.89^∗^) in RootSec. A statistically significant correlation between the expression of *cipk6* and total I content (*r* = 0.26^∗^) was found in leaves; however, no correlation was reported between the expression of *cipk6* and the content of T3 or 5-ISA. This led us to the conclusion that the root activity of *cipk6* is closely related to the presence of PDTHA. The effect of PDTHA on *cipk6* gene expression in leaves was smaller. This may be proof that the function of the dominant PDTHA receptor in aboveground parts of plants may be performed by a protein encoded by another gene/group of genes. However, the results of research presented herein are insufficient to thoroughly describe the physiological function of PDTHA in lettuce.

The highest expression of *cipk6* in roots and leaves of plants treated with 5-ISA was unequivocally associated with I uptake and metabolism in these plants. The plants were found to demonstrate the highest I-uptake and total I content, and had increased 5-ISA, 2-IBeA, 3,5-diISA, I^–^, and IO_3_*^–^* levels in roots and leaves. In addition, RootSec from plants where 5-ISA had been applied were also found to have the highest content of 5-ISA and I metabolites, such as I^–^, T3, and T4. The results clearly showed that there is a close interdependence between a larger preference to take up 5-ISA (compared with IO_3_*^–^* and 3,5-diISA) and the metabolism of I compounds which, among others, induces the synthesis of PDTHA and increases the expression of *cipk6*, a gene of the T3 and/or T4 receptor.

### Expression of the *msams5* Gene

The functions of S-adenosyl-l-methionine (SAM)-dependent halide methyltransferase (HMT) or SAM-dependent halide/thiol methyltransferase (HTMT), enzymes responsible for I volatilization to CH_3_I or CH_2_I_2_, are subject to a complex control mechanism and are typical of a number of marine algae species (Keng et al., 2020) and terrestrial plants (Attieh et al., 2000; Nagatoshi and Nakamura, 2007; Itoh et al., 2009; Landini et al., 2012). These enzymes are not specific for I as a substrate but can also participate in the methylation of other group-17 elements (halogens) from the periodic table. A different, complicated affinity in the substrate-enzyme system is as follows: I^–^ > Br^–^ > Cl^–^ (Manley, 2002; Murphy, 2003). In *B. oleracea*, HTMT was also involved in the process of methylation of [SH]- and [SCN]- groups to CH_3_SH (Attieh et al., 2000).

The available literature does not name the specific gene(s) responsible for I methylation in lettuce. The results justify the assignment of the potential HMT or HTMT function to an enzyme, S-adenosylmethionine synthase 5, encoded by the *msams5* gene. Characteristics of the *L. sativa* MSAMS5 protein are shown in Supplementary Data 3. Significant overexpression (about 8.5-fold higher than the control) of the *msams5* gene in the roots of plants treated with exogenous 5-ISA was detected. These results and, indirectly, the total root content of I and iodine metabolites show that a protein of the *msams5-*encoded enzyme may be immediately associated with I methylation by lettuce roots. Iodine volatilization through roots (with the participation of *msams5-*encoded enzyme) was higher following the application of iodosalicylates than when using KIO_3_. This indicates that the enzyme protein encoded by *msams5* might have played a dominant role in I methylation in roots but not in leaves. Compared with the control, foliar expression of *msams5* was slightly, yet significantly, increased only following the application of KIO_3_. Therefore, foliar expression of the *msams5* gene did not reflect the reported accumulation of I or its organic or non-organic compounds. The process of methylation (volatilization) of gaseous I from leaves probably occurred with the participation of the enzyme(s) encoded by a gene(s) other than *msams5.* On the basis of the transcriptome analysis (Kȩska et al., 2019), we were able to identify other genes in lettuce leaves that may potentially be associated with synthesis of enzymes with HMT- or HTMT-like function, i.e., with the synthesis of CH_3_I or CH_2_I_2_. The list includes the following genes described in the genome of lettuce: *S-adenosylmethionine synthase (XLOC_014031)* (Lsat_1_v5_gn_6_117861.1), *lysine-specific demethylase REF6 methyltransferase (XLOC_001612)* (Lsat_1_v5_gn_1_28820.1), *probable methyltransferase At1g27930 (XLOC_017217)* (Lsat_1_v5_gn_8_148061.1), and *histone-lysine N-methyltransferase (XLOC_011928)* (Lsat_1_v5_gn_5_154240.1).

Notably, *in vitro* emission of CH_3_I, CH_3_Br, and CH_3_Cl by rice depended on the stadium of the plants’ development (Redeker et al., 2004). Secretion of CH_3_I by rice during the day was nearly twice that observed at night (Muramatsu and Yoshida, 1995; Muramatsu et al., 1995). Gonzali et al. (2017) suggested that because the structure of HMT or HTMT and other methyltransferases is homologic, they are probably involved in plant salinity tolerance or play a role in the protection of plants against diseases. The interpretation assumed in the literature is that the process of I methylation (synthesis of CH_3_I or CH_2_I_2_) serves to detoxify plants from excess I content in tissues (Landini et al., 2012; Medrano-Macías et al., 2016; Gonzali et al., 2017). The results justify the presumption that the process of volatilization of gaseous I compounds, i.e., CH_3_I or CH_2_I_2_ (associated with HMT- or HTMT-like enzymatic activity) may also perform a different physiological function that is not yet described in the literature.

The activity of S-adenosylmethionine synthases, including those encoded by *msams5*, requires divalent cations, such as Mg^2+^, Mn^2+^, or Ca^2+^, and monovalent cations, such as K^+^ or Na^+^ (Supplementary Data 3). The V cation (VO^2+^) may replace divalent cations at the active site of this type of enzyme (Chasteen, 1995). Morrell et al. (1986) showed that biotransformation (oxidation) of V from vanadate (VO_3_*^–^*) to vanadyl (VO_2_^+^) during its uptake by plants is possible. In the three experiments conducted as part of the research, V was applied as VO_3_*^–^*. The research results obtained imply that VO_3_*^–^* transformation to VO_2_^+^ was weaker in the presence of exogenous 5-ISA and 3,5-diISA. This is indicated by reduced activity of *msams5*, which encodes an enzyme dependent on VO_2_^+^ and not on VO_3_*^–^*, and decreased V uptake by roots and leaves, in particular for the 3,5-diISA+V combination (in Experiment 1).

### Expression of *samdmt* Gene

The process of methylation (volatilization of methyl salicylic acid ester [MeSA]) occurs with the participation of an enzyme called salicylic acid carboxyl methyltransferase (SAMT) (Tieman et al., 2010). The synthesis of MeSA is one of the many processes in the production of SA derivatives in plants. MeSA participates in processes responsible for SAR. MeSA is volatilized from roots and overground parts of plants and can be transported by the phloem (Gao et al., 2014). The process of biosynthesis of MeSA is catalyzed by SA methyltransferases (SAMT/BSMT); the reconversion of MeSA back to SA by methyl esterase (MES, SABP2) is also possible (Park et al., 2009; Gao et al., 2014).

In our study the expression of the *S-adenosyl-L-methionine-dependent methyltransferase* gene (*samdmt)* (Supplementary Data 4) in lettuce leaves was clearly associated with application of exogenous SA, SA + KIO_3_ (KIO_3_+SA, KIO_3_+SA+V), and both iodosalicylates, 5-ISA and 3,5-diISA, with or without V. We reported a significant correlation between the foliar activity of *samdmt* and (1) the **RootSec** content of SA transported from roots to leaves (*r* = 0.80^∗^) and (2) the foliar content of SA and 5-ISA (*r* = 0.72^∗^ for SA and *r* = 0.77^∗^ for 5-ISA). Additionally, the results of measurements of all organic I metabolites and SA in roots and leaves showed that exogenous 5-ISA and 3,5-diISA underwent at least a 2-way transformation (Figures 5, 6). Conversely, 5-ISA and 3,5-diISA served as substrates in PDTHA synthesis, while they underwent a catabolic/decomposition reaction: (1) to I^–^ ions that could be used as substrates in CH_3_I or CH_2_I_2_ synthesis with the participation of the *msams5-*encoded enzyme or (2) to the SA molecule. This is indicated by the elevated SA content in roots and leaves and, consequently, by an increased foliar expression of *samdmt* in plants treated with exogenous 5-ISA or 3,5-diISA. Therefore, the results justify the assignment of a SAMTase-like function (esterification of SA; volatilization of MeSA) to the *samdmt* gene. The process was probably far more intense in leaves than in roots. This was exemplified by the relatively high expression of *samdmt* in leaves and trace functionality of the gene in the roots. For this very reason, no significant correlation was found between the activity of *samdmt* and root content of SA, 5-ISA, and 3,5- diISA.

## Conclusion

The direction of metabolic conversion of KIO_3_, 5-ISA, and 3,5-diISA in plants was documented. Both iodosalicylates were applied exogenously and underwent degradation inside the plants to I ions or served as precursors of synthesis of T3 and T4, classified as PDTHAs. The assignment of the role of encoding protein receptor T3 or T4, mainly in lettuce roots, to *cipk6* was proposed.

There are reasons to believe that the *per64-like*, rather than the *per12-like* gene, may act as a V-dependent haloperoxidase (vHPO), an enzyme that participates in I uptake (expression of *per64-like* gene in roots > leaves). The expression of *msams5* was sufficiently specific to link the gene to the functions of HMT/HTMT enzymes. This gene was overexpressed in roots in systems where exogenous iodosalicylates were applied. The expression of *samdmt*, in turn, makes it naturally shortlisted for the role of a gene encoding the enzyme responsible for esterification/volatilization of ethyl salicylic acid (activity: leaves > roots).

V added to the nutrient solution caused a significant reduction and growth of I content in roots, but not in leaves, for the combination of: KIO_3_+SA+V vs. KIO_3_+SA and 3,5-diISA+V vs. 3,5-diISA, respectively. V was mostly accumulated in roots, with its transfer to leaves being limited. The level of *per64-like* expression was correlated with root activity of vHPO.

Plant enrichment with I through 5-ISA and 3,5-diISA was more effective than that through KIO_3_. The results of pot experiments indicated that the I compounds tested, including 5-ISA and 3,5-diISA, in particular, may be used in I enrichment of plants through fertigation without the fear of harming the plants. The level of plant enrichment in I was safe for consumers. This is implied by the fact that the highest HQ-I in pot studies was 0.071. Consumers’ safety would be at risk if the HQ exceeded 1.0. In Experiment 1 (hydroponic system), the efficacy of I uptake from the nutrient solution was higher than in the mineral soil or peat substrate. However, in the context of balancing the reference daily allowance of I for humans, the achieved level of I accumulation (especially following application of 5-ISA) was too high [as shown by RDA-I (%) > 480%, HQ > 0.66]. This means that doses < 10 μM of I compounds can be recommended for hydroponic systems, especially where both iodosalicylates are used.

I-enriched lettuce strongly reduces the *in vitro* development of cancerous cells in colon cancer (Koronowicz et al., 2016). It seems appropriate to study the use of lettuce enriched with 5-ISA and 3,5-diISA in nutrigenomics.

## Data Availability Statement

The datasets presented in this study can be found in the online repositories. The names of the repository/repositories and accession number(s) can be found in the article/Supplementary Material.

## Author Contributions

SS, MC, IK, and JP: methodology. SS, MC, and KK: formal analysis. SS: funding acquisition. SS, IK, MH, MG, ŁS, JP, and AK: investigation of lettuce cultivation and chemical analyses of plant, nutrient solution, and soil samples. MC, KK, and DG: investigation of molecular analyses of lettuce. SS, MC, IK, and KK: resources. SS and MC: supervision. SS, MC, and KK: writing—original draft. SS, MC, IK, KK, MH, MG, DG, ŁS, and PK: writing—review and editing. All authors contributed to the article and approved the submitted version.

## Conflict of Interest

The authors declare that the research was conducted in the absence of any commercial or financial relationships that could be construed as a potential conflict of interest.

## Footnotes

1 https://primer3plus.com/cgi-bin/dev/primer3plus.cgi

2 https://lgr.genomecenter.ucdavis.edu

3 https://eu.idtdna.com/calc/analyzer

4 https://www.tibco.com/products/data-science

## Supplementary Material

Supplementary Data 1 The functional annotation of *per12-like i per64-like* genes.

Supplementary Data 2 The functional annotation of *cipk6* gene.

Supplementary Data 3 The functional annotation of *msams5* gene.

Supplementary Data 4 The functional annotation of *samdmt* gene.

## Glossary

2,3,5-triIBeA = 2,3,5-triiodobenzoic acid

2-IBeA = 2-iodobenzoic acid

3,5-diISA = 3,5-diiodosalicylic acid

4-IBeA = 4-iodobenzoic acid

5-ISA = 5-iodosalicylic acid

BeA = benzoic acid

HMT = halide methyltransferase

HPLC-ICP-MS)/MS = high-performance liquid chromatography with inductively coupled plasma mass spectrometry

HPOs = haloperoxidases

HTMT = SAM-dependent halide/thiol methyltransferase

ICP-OES = inductively coupled plasma optical emission spectrometry

I-Tyr = iodotyrosine

LC-MS/MS = liquid chromatography-mass spectrometry

MAPK = mitogen-activated protein kinase

MeSA = volatile ester of methyl salicylic acid

NFT = nutrient film technique

PDTHA = plant-derived thyroid hormone analogs

RDA = recommended dietary allowance

RootSec = secretion produced as a result of root pressure

SA = salicylic acid

SAM = S-adenosyl-l-methionine

SAMT = salicylic acid carboxyl methyltransferase

SAR = Systemic Acquired Resistance

SOM = soil organic matter

T3 = triiodothyronine

T3-Na = sodium salt triiodothyronine

T4 = thyroxine

TF = transfer factor

TMAH = tetramethylammonium hydroxide

TransThy-T3/T4trans = transthyretin, responsible for T3 and T4 transport

vHPO = vanadium-dependent haloperoxidases

per64-like = Peroxidase 64-like

per12-like = Peroxidase 12-like.

samdmt = S-adenosyl-L-methionine-dependent methyltransferase

cipk6 = CBL-interacting serine/threonine-protein kinase 6

msams5 = S-adenosylmethionine synthase 5
