# Supplementary material for: New Aspects of Uptake and Metabolism of Non-organic and Organic Iodine Compounds—The Role of Vanadium and Plant-Derived Thyroid Hormone Analogs in Lettuce
Source: Front Plant Sci. 2021 Apr 16;12:653168. doi: 10.3389/fpls.2021.653168 (PMC8086602; doi:10.3389/fpls.2021.653168)

New aspects of uptake and metabolism of non-organic and organic iodine compounds – the role of vanadium and plant-derived thyroid hormone analogs in lettuce

Sylwester Smoleń[1], Małgorzata Czernicka[1], Iwona Kowalska[1], Kinga Kęska[1], Mariya Halka[1], Marlena Grzanka[1], Dariusz Grzebelus[1], Łukasz Skoczylas[2], Joanna Pitala[3], Aneta Koronowicz[4], Peter Kováčik[5]

[1]Department of Plant Biology and Biotechnology, Faculty of Biotechnology and Horticulture, University of Agriculture in Krakow, Kraków, Poland

[2]Department of Plant Product Technology and Nutrition Hygiene, Faculty of Food Technology, University of Agriculture in Krakow, Balicka 122, 30-149 Krakow, Poland

[3]Laboratory of Mass Spectrometry, Faculty of Biotechnology and Horticulture, University of Agriculture in Krakow, Al. 29 Listopada 54, 31-425 Krakow, Poland

[4]Department of Human Nutrition and Dietetics, Faculty of Food Technology, University of Agriculture in Krakow, Balicka 122, 30-149 Krakow, Poland

[5]Department of Agrochemistry and Plant Nutrition, Slovak University of Agriculture in Nitra, Tr. A. Hlinku 2, 949 01 Nitra, Slovakia

Complement to "Materials and Methods"

Methods of analyzing chemical properties of soil

To characterize the chemical properties of soil (see section "Plant material and treatments"), the following chemical analyses were carried out in soil samples taken before lettuce planting: soil texture, hydrolytic exchange acidity, cation exchange capacity, total soil sorption capacity, acidification ($pH_{(H2O)}$ and $pH_{(KCl)}$), salinity (EC) ($mS \cdot cm^{-1}$), soil oxyreduction potential (Eh mV), organic matter content, and mineral composition (Nowosielski 1988). The content of macroelements: $N\text{-}NH_4$, $N\text{-}NO_3$, $N\text{-}NH_4$+ $N\text{-}NO_3$, P, K, Mg, Ca, and S was analyzed using the methods and technologies presented by Smoleń et al. (2016).

Soil content of hydroxides, Al, Fe, and Mn was analyzed according to methods by Kostka and Luther (1994), and Anschutz et al. (1998). Three-tenths of a gram of dry soil was weighed into 30 mL falcon tubes. Then 25 mL of extraction solution was added. The extraction solution was composed of 0.22 M trisodium citrate, 0.11 M sodium bicarbonate, and 0.1 M sodium dithionite. The samples were shaken at 45°C for 22 hours. Then they were centrifuged for 20 minutes at 3000 rpm and filtered using medium qualitative filters. Al, Fe, and Mn were analyzed using ICP-OES.

The content of BeA, SA, 5-ISA, 3,5-diISA, 2-IBeA, 4-IBeA, and 2,3,5-triIBeA in soil samples was analyzed using the same procedure as for samples of plant material (see section: "Determination of salicylic acid, benzoic acid, iodosalicylates, iodobenzoates, and plant-derived thyroid hormone analogs").

The content of I and V in peat substrate and soil samples collected before lettuce cultivation (average mixed sample) and after cultivation (separately for each combination) was determined using spectrometry. Iodine content was determined after soil extraction by TMAH. The methodology used for this purpose was the same as for plant samples (see section "Analysis of total iodine and vanadium in dry samples of roots and leaves"). Vanadium content in soil was measured using the Rinkis method, after extraction with 1 M HCl

solution (the soil-to-extractor ratio was 1:10). The extraction lasted 1 hour. A 10 g soil sample was shaken in 100 mL 1 M HCl at 30 rpm in 500 mL polypropylene bottles. Next, the extract was filtered through a medium qualitative filter to falcon tubes and analyzed with ICP-OES (Prodigy Teledyne Leeman Labs USA spectrometer ICP-OES), as described by Smoleń et al. (2016).

References to methods of analyzing chemical properties of soil:

Anschutz, P., Zhon, S.J., Sundby, B., Mucci, A., Gobeil, C. (1998), Burial efficiency of phosphorus and the geochemistry of iron in continental margin sediments. *Limnol. Oceanogr.* 43, 53–64. doi: 10.4319/lo.1998.43.1.0053

Kostka, J.E., Luther, G.W. (1994). Partitioning and speciation of solid-phase iron in salt-marsh sediments. *Geochim. Cosmochim. Acta* 58, 1701–1710. doi: 10.1016/0016-7037(94)90531-2

Nowosielski O (1988) The rules in development of fertilizing strategies in horticulture. PWRiL Publisher, Warsaw (In Polish)

Smoleń, S., Skoczylas, Ł., Ledwożyw-Smoleń, I., Rakoczy, R., Kopeć, A., Piątkowska, E., et al. (2016). Iodine and selenium biofortification of lettuce (*Lactuca sativa* L.) by soil fertilization with various compounds of these elements. *Acta Scient. Polon. Hort. Cult.* 15(5), 69–91.

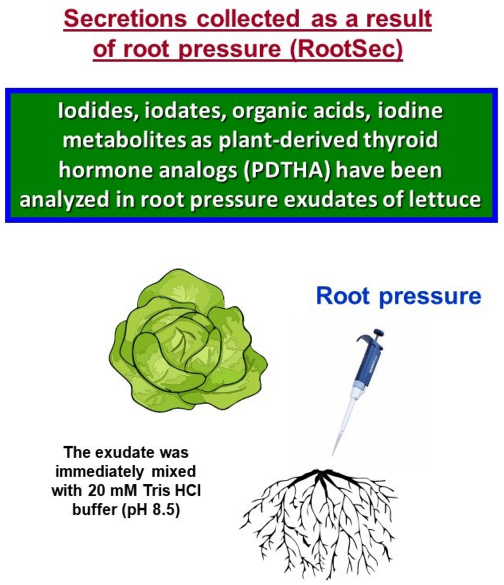

Figure S1. Method collection of secretions collected as a result of root pressure (RootSec)

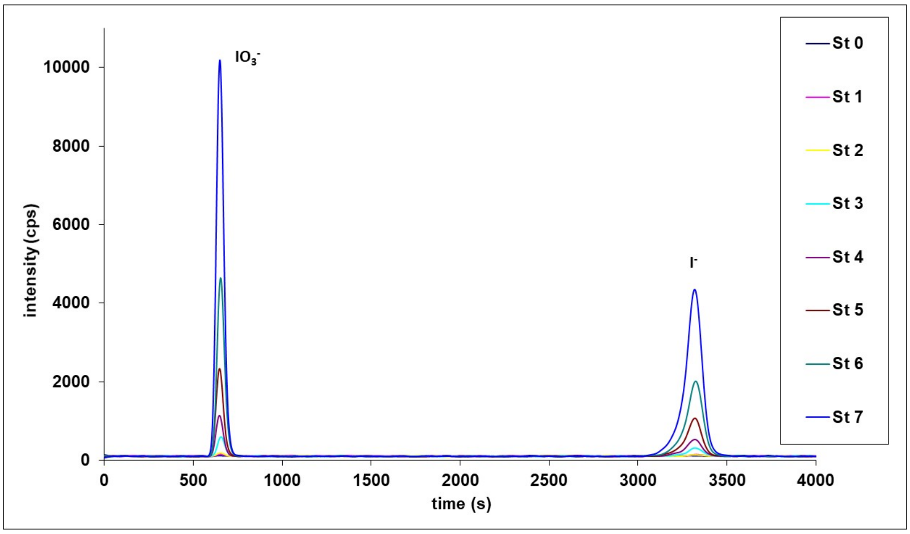

Figure S2. Determination of I^- and IO_3^- by HPLC-ICP-MS. Chromatograms of I^- and IO_3^- standards. Concentration of I^- and IO_3^- in standards were: St0 – 0 ppb, St1 – 0.1 ppb, St2 – 0.5 ppb, St3 – 1 ppb, St4 – 5 ppb, St5 – 10 ppb, St6 – 25 ppb, St7 – 50 ppb, St3 – 100 ppb (all concentration calculation on iodie weight).

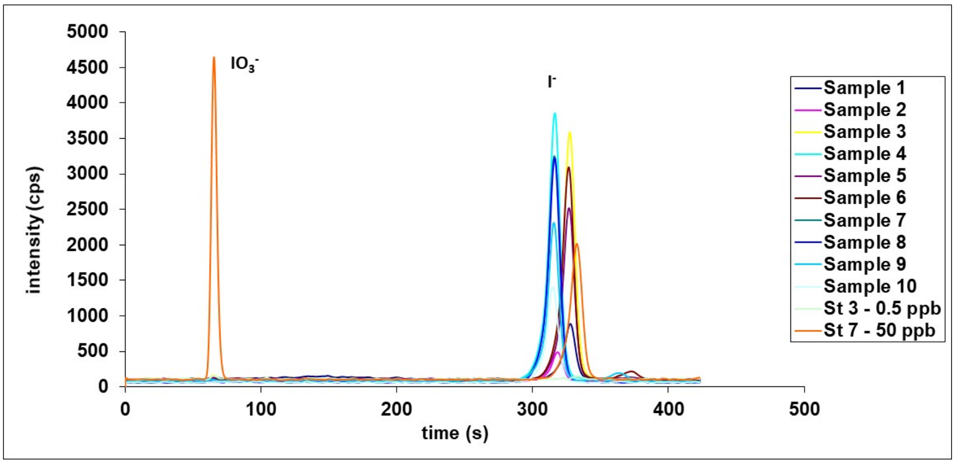

Figure S3. Determination of I^- and IO_3^- by HPLC-ICP-MS. Chromatograms of 10 different samples and standard 0.5 ppb and 50 ppb of I^- and IO_3^- (calculation on iodie weight).

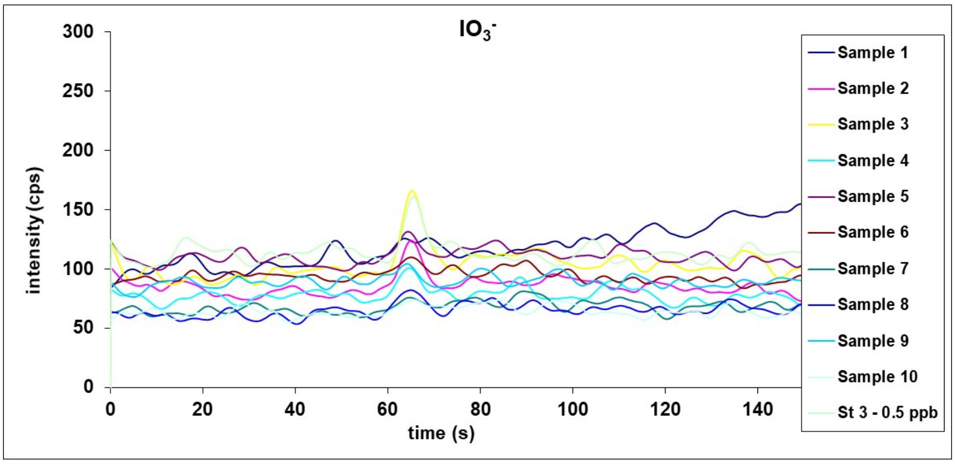

Figure S4. Determination of I^- and IO_3^- by HPLC-ICP-MS. Chromatograms of 10 different samples and standard 0.5 ppb, showing only peak for IO_3^- ion.

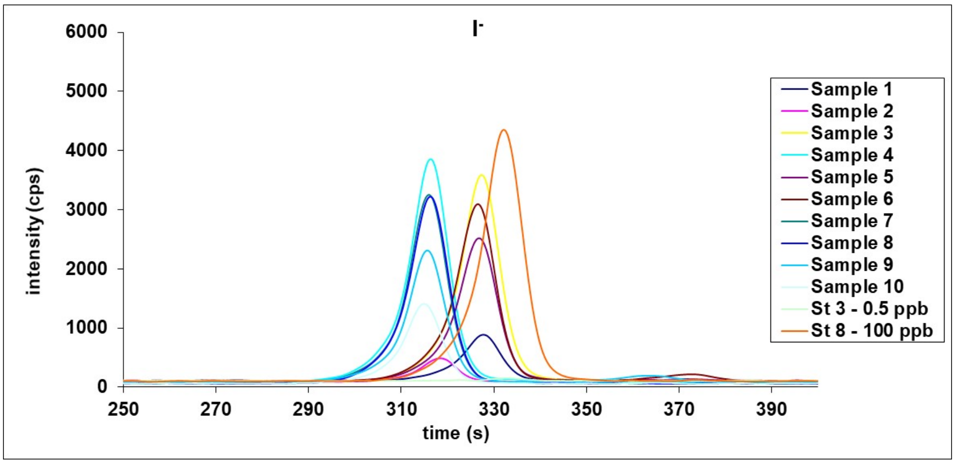

Figure S5. Determination of I^- and IO_3^- by HPLC-ICP-MS. Chromatograms of 10 different samples and standard 0.5 ppb and 100 ppb, showing only peak for I^- ion.

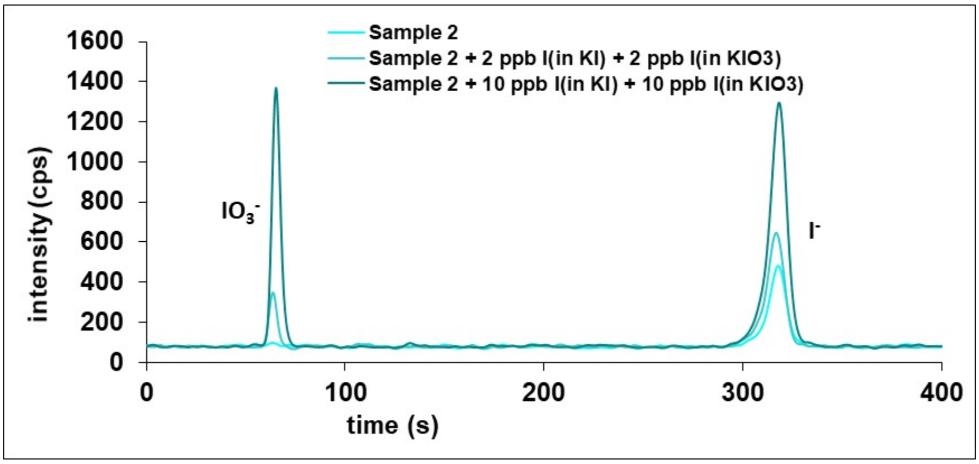

Figure S6. Determination of I^- and IO_3^- by HPLC-ICP-MS. Chromatograms of sample No. 2 and sample No. 2 with a standard addition.

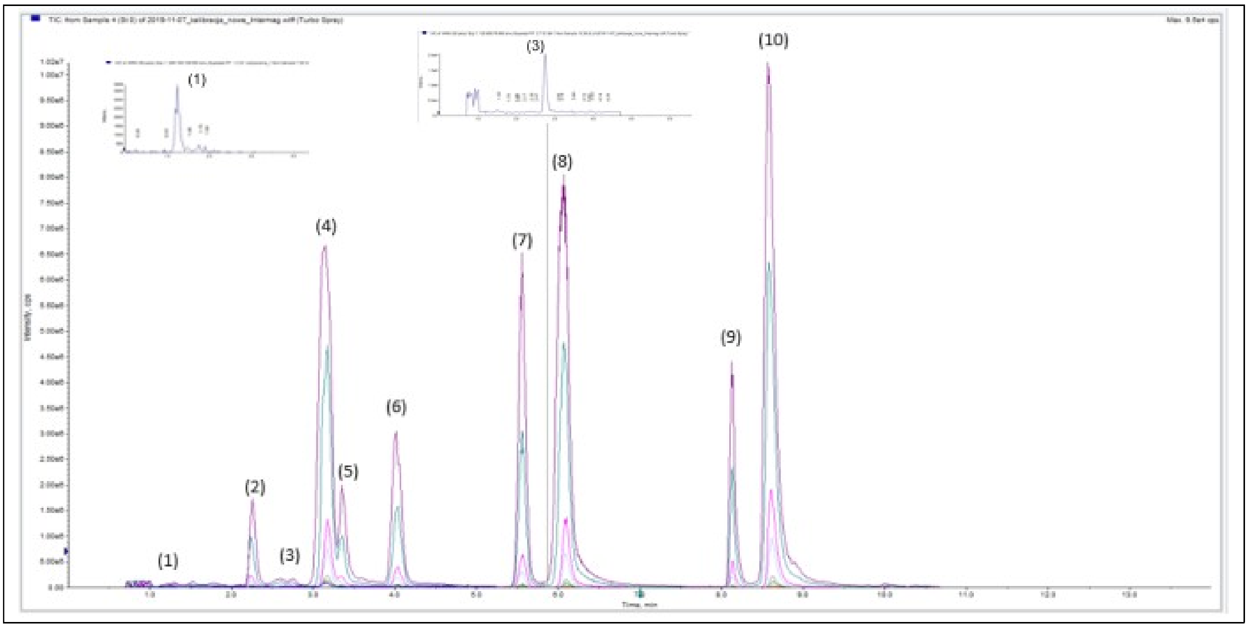

Figure S7. Determination of iodine compounds by HPLC-MS/MS. Chromatograms (TIC – total ion count) of standards of listed compounds: (1) I-Tyr, (2) T3-sodium salt and T3, (3) BeA, (4) SA and SA-d4, (5) T4, (6) 2-IBeA, (7) 4-IBeA, (8) 5-ISA, (9) 2,3,5-triBeA, (10) 3,5-diISA.

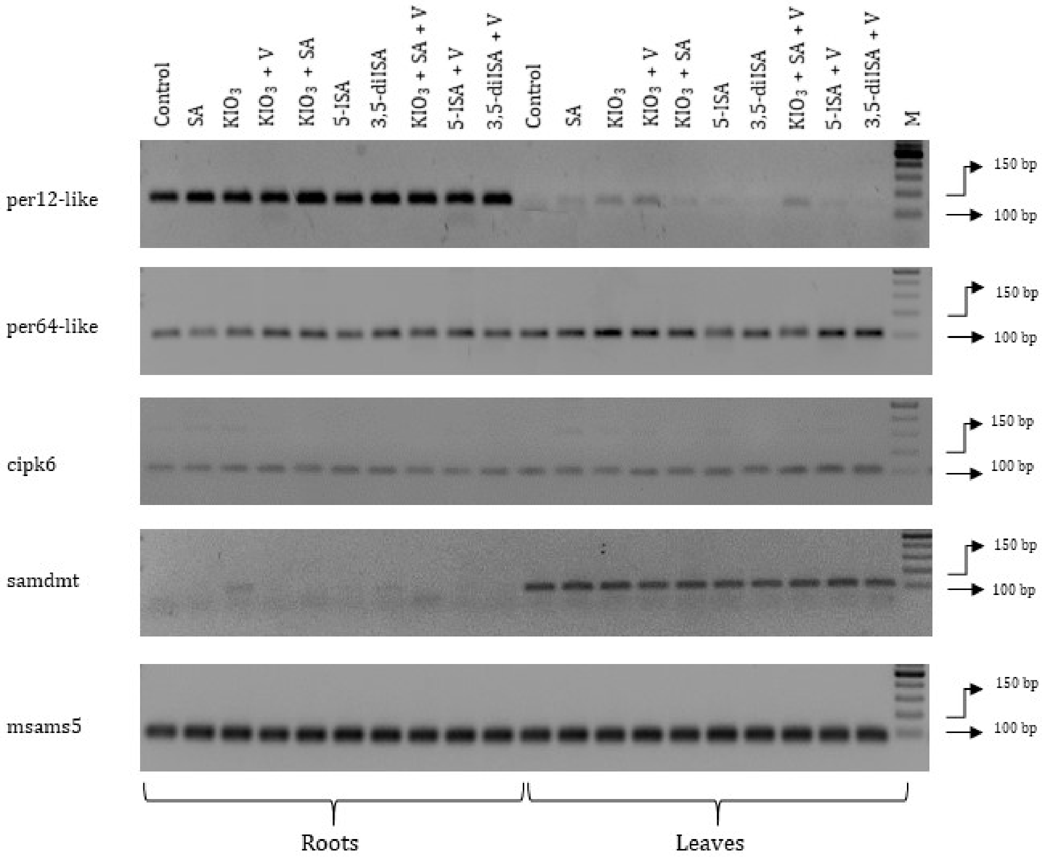

Figure S8. Confirmation of the primers specificity and amplicon sizes for five investigated genes based on electrophoresis of RT-PCR products. M: 100 bp ladder. See Table S1 for product sizes.
Comment: The order of the gel bands assigned to each treatment is different than the order in all others tables and figures in this publication.

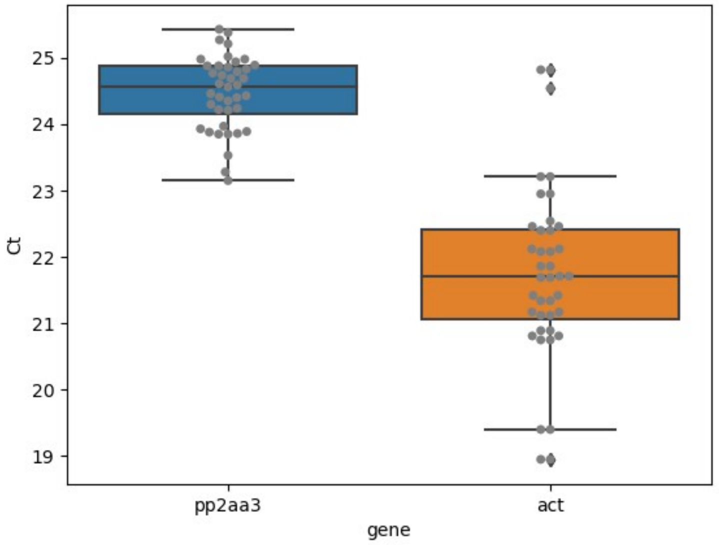

Figure S9. Box-plot graph for the Ct values of two tested reference genes, i.e. protein phosphatase 2A regulatory subunit A3 (*pp2aa3*) and actin (*act*) collected for all samples in qRT-PCR.

Table S1. Sequences of genes and primers used in the study

Gene	Symbol	Chromosome	NCBI Accession Number	Gene Model in Lettuce Genome Resource**	Sequence (5'--->3')	Product size (bp)
Peroxidase 12-like	*per12-like*	6	MT649253*	Lsat_1_v5_gn_6_101900.1	F:GGATCTTCGTAGTCGGGTTA<br>R:GTCCACCAGTCAAGACAATG	104
Peroxidase 64-like	*per64-like*	4	MT649254*	Lsat_1_v5_gn_4_144120.1	F:ACTCACTAACCCAACGACTAA<br>R:CTCTACAATTCAATCTAACCTCCTG	140
S-adenosyl-L-methionine-dependent methyltransferase	*samdmt*	4	MT663550*	Lsat_1_v5_gn_4_119301.1	F:TGCTGTTCGGACTTTATGG<br>R:TTGTGTCCATTCCTGCAC	101
CBL-interacting serine/threonine-protein kinase 6	*cipk6*	8	MT663549*	Lsat_1_v5_gn_8_61661.1	F:ACTTTCAGCAACTCATCTCC<br>R:CCTTCGCCGTCTAACAATAA	97
S-adenosylmethionine synthase 5	*msams5*	6	MT663551*	Lsat_1_v5_gn_6_117861.1	F:TCCAGTTCCATAAGTGTCAACAAAC<br>R:CATTGTGGCTTGTGGGTTGG	103
Actin	*act*	8	AB359898.1	Lsat_1_v5_gn_8_116260.1	F:AGGTGTCATGGTTGGCATGGGA<br>R:TGTTCTTCAGGGGCGACACG	180
Protein phosphatase 2A regulatory subunit A3	*pp2aa3*	8	XM_023912511.1	Lsat_1_v5_gn_8_38881.1	F:CATGCAATGGTTACAAGACAAGGTAT<br>R:CAAACTCCTCCGCAAGTCTCTTC	80

* Transcript sequences of genes deposited in GenBank assembled from RNAseq data of the *Lactuca sativa* var. *capitata* 'Melodion' of control plants and supplemented with SA, KIO_3 and KIO_3+V in this experiment
**The Lettuce Genome Resource (LGR), https://lgr.genomecenter.ucdavis.edu

Table S2. Percentage of Recommended Daily Allowance (RDA) for iodine (RDA-I) in 100 g portion of fresh lettuce leaves (150 µg – the daily iodine requirement of adults) as well as Hazard Quotient (HQ) for intake of I through the consumption of 100 g of fresh lettuce leaves by adults (70 kg body weight) – average for Experiment No. 1, 2 and 3.

Treatments	Hydroponics NFT Experiment No. 1		Peat substrate Experiment No. 2		Mineral soil Experiment No. 3	
	RDA-I (%)	HQ-iodine	RDA-I (%)	HQ-iodine	RDA-I (%)	HQ-iodine
Control	9.4±1.4a	0.013±0.002a	1.5±0.5a	0.002±0.0007a	9.7±0.5a	0.013±0.0007a
SA	4.1±0.5a	0.006±0.001a	0.6±0.2a	0.001±0.0002a	9.3±0.5a	0.013±0.0006a
KIO_3	53.3±3.5b	0.073±0.005b	13.0±2.4c	0.018±0.0033c	39.3±3.8c	0.054±0.0052c
KIO_3+SA	73.3±9.2b	0.100±0.013b	13.1±2.1c	0.018±0.0029c	45.4±6.2d	0.062±0.0085d
5-ISA	483.9±25.1c	0.661±0.034c	36.1±4.7e	0.049±0.0065e	52.0±5.2f	0.071±0.0072f
3,5-diISA	69.5±15.5b	0.095±0.021b	10.8±2.2bc	0.015±0.0030bc	37.7±0.5bc	0.051±0.0006bc
KIO_3+V	52.4±3.9b	0.071±0.005b	11.5±2.2bc	0.016±0.0030c	37.8±1.9bc	0.052±0.0027bc
KIO_3+SA+V	52.8±4.4b	0.072±0.006b	9.3±1.3b	0.013±0.0018b	35.9±1.2b	0.049±0.0016b
5-ISA+V	499.5±61.8c	0.682±0.084c	31.0±3.3d	0.042±0.0045d	49.6±2.3e	0.068±0.0031e
3,5-diISA+V	69.9±6.1b	0.095±0.008b	13.2±2.0c	0.018±0.0027c	49.9±8.7e	0.068±0.0119e

Means in the column followed by different letters separately for each experiments differ significantly at $P < 0.05$ (n=8). The hazard to consumer exists when the value of HQ exceeds 1.0.

Table S3. Iodine and vanadium uptake by single heads /leaves/, roots and by whole plants /roots+ head/ from one plants in hydroponic NFT Experiment No. 1 as well as by single head /leaves/ of lettuce plants in pot Experiment No. 2 and 3.

Treatments	Hydroponics NFT Experiment No. 1			Peat substrate Experiment No. 2	Mineral soil Experiment No. 3
	Iodine uptake:				
	(μg I·leaves·plant^{-1})	(μg I·roots·plant^{-1})	(μg I· whole plants^{-1})	(μg I·leaves·plant^{-1})	(μg I·leaves·plant^{-1})
Control	36.5±2.8ab	16.1±5.1a	52.5±7.5a	3.3±1.1a	26.2±2.3a
SA	16.6±0.7a	29.5±10.1a	46.1±10.2a	1.1±0.3a	24.3±3.3a
KIO_3	225.2±14.0d	74.8±6.5a	300.0±10.9b	30.6±4.5b	97.5±4.5bc
KIO_3+SA	265.0±13.3d	96.5±15.1a	361.5±16.9b	29.4±3.8b	106.1±4.5bc
5-ISA	1 165.1±205.9f	1 124.4±202.4d	2 289.5±407.6d	86.5±7.9d	142.2±4.4e
3,5-diISA	93.2±5.6bc	557.8±87.1b	651.0±82.8c	25.0±3.7b	104.5±10.8bc
KIO_3+V	227.5±9.5d	73.7±8.8a	301.1±11.4b	26.5±3.7b	101.8±5.9bc
KIO_3+SA+V	229.6±5.6d	77.5±5.2a	307.1±7.9b	21.1±1.6b	89.8±8.9b
5-ISA+V	974.9±120.3e	1 421.5±449.6e	2 396.4±340.8d	77.7±5.2c	124.1±8.9d
3,5-diISA+V	133.9±29.3c	710.2±92.0b	844.2±68.1c	31.3±3.1b	115.7±8.5cd
	Vanadium uptake:				
	(μg V·leaves·plant^{-1})	(μg V·roots·plant^{-1})	(μg V· whole plants^{-1})	(μg V·leaves·plant^{-1})	(μg V·leaves·plant^{-1})
Control	14.07±1.02cd	1.37±0.10b	15.44±1.10bc	15.59±5.58a	21.57±7.02b
SA	13.17±0.86c	1.70±0.20b	14.86±0.72bc	15.76±5.74a	14.70±4.90 ab
KIO_3	13.49±1.13c	1.79±0.25b	15.28±0.90bc	16.10±5.78a	14.69±4.90a
KIO_3+SA	15.15±1.52de	1.44±0.19b	16.59±1.40c	15.71±5.74a	14.27±4.65a
5-ISA	10.28±1.43b	2.13±0.48b	12.41±0.96ab	15.19±5.46a	16.99±5.47 ab
3,5-diISA	8.91±1.57ab	0.66±0.11a	9.57±1.67a	16.37±6.03a	17.83±5.59 ab
KIO_3+V	16.03±1.04e	7.99±0.94e	24.02±1.13e	16.44±5.84a	17.12±5.74 ab
KIO_3+SA+V	15.86±0.65e	6.90±0.77d	22.76±0.57de	16.33±5.99a	18.11±6.25 ab
5-ISA+V	9.67±1.39ab	10.45±0.40f	20.12±1.37d	15.66±5.59a	19.27±6.43 ab
3,5-diISA+V	8.58±1.55a	5.27±1.17c	13.86±2.69bc	15.18±5.39a	17.67±5.83 ab

Means in the column followed by different letters separately for each experiments differ significantly at $P < 0.05$ (n=8).

Content of BeA, SA, and iodine metabolites in roots and leaves

The I, V, and SA compounds analyzed had a diversified impact on the content of SA, 5-ISA, 3,5-diISA, I-Tyr, T3-Na, T3 (Table S4), BeA, 2-IBeA, 4-IBeA, and 2,3,5-triIBeA (Table S5) in roots in Experiment 1, and in leaves in all 3 experiments. It should be emphasized that all the organic iodine compounds subjected to analysis (Tables S4 and S5) were present in control plants in each of the 3 experiments. This shows that the compounds are naturally synthesized in lettuce. Additionally, T3-Na content in leaves and roots was at all times higher than the content of T3. The samples of lettuce leaves and roots were not found to contain T4; or the level of T4 was below the limit of quantification (LOQ for T4 = 1913.304 $ng \cdot mL^{-1}$).

In each of the experiments, exogenous application of 5-ISA and 3,5-diISA resulted in the highest contents of these iodosalicylates (for 5-ISA and 3,5-diISA treatments, respectively) in leaves and roots in Experiment 1. It needs to be added that application of 3,5-diISA resulted in the greatest accumulation of the compound in both roots and leaves. Additionally, the root content of 5-ISA following fertilization with 3,5-diISA was higher than in control (Table S4), but was still several times lower than after application of exogenous 5-ISA, when the accumulation of 5-ISA in roots and leaves was the highest. Finally, exogenous 3,5-diISA led to a significant increase in T3-Na content in roots and leaves and to a significant growth of root 2,3,5-triIBeA concentration in hydroponic plants (Experiment 1 – Table S4). The results obtained also show that exogenous 3,5-diISA may be conversed to: A) 5-ISA, and then to SA, BeA, and 2,3,5-triIBeA (Tables S4 and S5). This conclusion has been drawn based on root content of these compounds determined in Experiment 1.

For plants treated with exogenous 5-ISA, conversion of 5-ISA to 3,5-diISA and 2-IBeA was observed in lettuce roots (Experiment 1; Tables S4 and S5), but not in the leaves.

Experiment 3 was the only experiment where the content of I-Tyr and 3,5-diISA increased significantly following the application of KIO_3 (without V or SA) (Table S4).

A comparison of 5-ISA+V versus 5-ISA showed that additional vanadium fertilization significantly increased 5-ISA, T3, and BeA content in leaves (only in Experiment 1), and caused a significant increase in 5-ISA, T3, BeA, 2-IBeA as well as decrease in 3,5-diISA levels in roots in Experiment 1 (Tables S4 and S5).

A comparison of 3,5-diISA+V versus 3,5-diISA after additional vanadium fertilization in turn showed a significant statistical decrease in 3,5-diISA content in leaves in each of the 3 experiments (Table S4). Finally, a comparison of KIO_3 *versus* KIO_3+V and KIO_3+SA *versus* KIO_3+SA+V did not reveal any distinct, specific changes in the metabolism/synthesis of SA, BeA, iodosalicylates, or iodobenzoates like the ones reported for fertilization with ammonium metavanadate combined with 5-ISA or 3,5-diISA; a comparison with control was also made (Tables S4 and S5).

It must be stated that the percentage of the sum of all analyzed organic iodine to total iodine content was as follows: A) within the range from 0.40% for KIO_3+SA to 9.18% for 3,5-diISA for roots; B) within the range from 0.16% for KIO_3+SA to 7.01% for 3,5-diISA+V for leaves (Table 5 - see manuscript). In addition, roots were characterized by a much higher theoretical percentage share of the content of other unanalyzed organic or inorganic iodine compounds in total iodine content than leaves. In roots, the rate ranged from 74.0% for KIO_3+SA to 99.0% for SA. In leaves, it was between 15.3% for KIO_3+SA+V and 93.9% for 3,5-diISA. The results presented in Table 5 indicate that in each of the combinations analyzed the metabolism of organic and mineral iodine compounds varied in terms of the pace and directions of conversion of iodine compounds.

Table S4 Concentrations of salicylic acid (SA), 5-iodosalicylic acid (5-ISA), 3,5-diiodosalicylic acid (3,5-diISA), iodotyrosine (I-Tyr), sodium salt triiodothyronine (T3-Na) and triiodothyronine (T3) in leaves /head/ and roots of lettuce cultivated in hydroponics NFT Experiment No. 1 as well as in leaves of plants cultivated in pot Experiment No. 2 and 3

Experiment No. / Part of plant	Treatments	(mg·kg^{-1} D.W.)					
		SA	5-ISA	3,5-diISA	I-Tyr	T3-Na	T3
1. Hydroponic NFT / Leaves	Control	0.52±0.05d	0.046±0.009a	0.044±0.008a	0.268±0.033d	0.354±0.040bc	0.008±0.001bc
	SA	0.32±0.02a	0.006±0.001a	0.032±0.009a	0.028±0.005a	0.172±0.066a	0.001±0.000a
	KIO_3	0.45±0.03cd	0.021±0.003a	0.195±0.069b	0.561±0.141e	0.128±0.074a	0.015±0.002de
	KIO_3+SA	0.41±0.04bc	0.008±0.001a	0.023±0.007a	0.061±0.014ab	0.256±0.057ab	0.002±0.000ab
	5-ISA	1.10±0.08g	1.557±0.085c	0.014±0.003a	0.053±0.011ab	0.257±0.129ab	0.009±0.001cd
	3,5-diISA	1.04±0.12fg	0.385±0.018b	12.679±2.946d	0.034±0.005a	0.643±0.170cd	0.018±0.003e
	KIO_3+V	0.63±0.05e	0.007±0.001a	0.028±0.007a	0.138±0.021bc	0.302±0.190ab	0.011±0.001cd
	KIO_3+SA+V	0.33±0.02ab	0.009±0.001a	0.029±0.010a	0.220±0.038cd	0.938±0.055d	0.001±0.001a
	5-ISA+V	0.97±0.10f	1.728±0.073d	0.065±0.024ab	0.108±0.042ab	0.234±0.146ab	0.020±0.001e
	3,5-diISA+V	1.02±0.04fg	0.357±0.017b	11.307±2.675c	0.040±0.003ab	0.520±0.130bc	0.019±0.001e
1. Hydroponic NFT / Roots	Control	1.45±0.32a	0.031±0.0034a	0.745±0.239a	0.045±0.008ab	5.493±0.295a	0.046±0.007a
	SA	2.05±0.64a	0.029±0.0009a	0.469±0.106a	0.050±0.003abc	3.112±0.130a	0.096±0.033a
	KIO_3	1.99±0.51a	0.027±0.0039a	0.321±0.050a	0.086±0.005bcd	3.340±0.690a	0.049±0.020a
	KIO_3+SA	3.03±0.99b	0.030±0.0060a	0.068±0.006a	0.093±0.012cd	4.522±0.664a	0.054±0.007a
	5-ISA	10.83±3.89b	10.957±2.7669c	11.983±4.317b	0.090±0.016cd	4.929±0.913a	0.084±0.023a
	3,5-diISA	14.20±4.21e	2.531±0.6405b	595.896±99.78d	0.027±0.007a	9.150±0.655b	0.048±0.012a
	KIO_3+V	1.92±0.58a	0.060±0.0115a	4.683±0.844b	0.114±0.011d	2.802±0.350a	0.079±0.004a
	KIO_3+SA+V	1.82±0.56a	0.075±0.0246a	3.777±1.127b	0.121±0.005d	3.802±0.306a	0.052±0.012a
	5-ISA+V	12.04±4.25b	19.209±5.2848d	2.867±0.410b	0.121±0.010d	3.541±0.674a	0.282±0.081b
	3,5-diISA+V	15.38±4.75f	2.416±0.5330b	504.840±52.72c	0.045±0.004ab	8.876±0.523b	0.077±0.013a
2. Peat substrate / Leaves	Control	0.42±0.03a	0.020±0.006ab	0.033±0.012b	0.021±0.004b	0.634±0.124abc	0.044±0.034c
	SA	0.28±0.01a	0.012±0.002a	0.004±0.001a	0.019±0.004ab	0.102±0.083a	0.017±0.004ab
	KIO_3	0.37±0.01a	0.017±0.002ab	0.022±0.003ab	0.020±0.003ab	1.115±0.416cd	0.013±0.004ab
	KIO_3+SA	0.32±0.02a	0.015±0.003a	0.009±0.002ab	0.013±0.004ab	0.801±0.367ab	0.007±0.004a
	5-ISA	0.33±0.01a	0.224±0.035d	0.010±0.001ab	0.018±0.004ab	1.491±0.401d	0.012±0.007ab
	3,5-diISA	0.37±0.02a	0.017±0.006ab	0.352±0.066d	0.019±0.007ab	0.776±0.264abc	0.023±0.009bc
	KIO_3+V	0.31±0.02a	0.013±0.004a	0.009±0.003ab	0.017±0.006ab	0.351±0.032ab	0.035±0.016c
	KIO_3+SA+V	0.27±0.01a	0.010±0.002a	0.010±0.002ab	0.018±0.002ab	0.382±0.073ab	0.009±0.002ab
	5-ISA+V	0.31±0.01a	0.204±0.017c	0.013±0.003ab	0.011±0.001a	0.543±0.162abc	0.017±0.007ab
	3,5-diISA+V	0.26±0.01a	0.015±0.004a	0.206±0.025c	0.009±0.002a	0.432±0.170abc	0.022±0.003bc
3. Mineral soil / Leaves	Control	0.27±0.02a	0.040±0.009bc	0.003±0.001a	0.004±0.001a	0.463±0.255a	0.015±0.008a
	SA	0.26±0.00a	0.026±0.003abc	0.008±0.002ab	0.012±0.002abc	0.894±0.348b	0.023±0.010a
	KIO_3	0.37±0.01a	0.026±0.005abc	0.012±0.003ab	0.026±0.006d	0.630±0.308a	0.010±0.004a
	KIO_3+SA	0.24±0.01a	0.028±0.008abc	0.005±0.002a	0.021±0.005cd	0.904±0.272b	0.013±0.002a
	5-ISA	0.41±0.13a	0.169±0.027e	0.012±0.004ab	0.004±0.003a	0.530±0.073a	0.011±0.007a
	3,5-diISA	0.28±0.02a	0.016±0.003ab	0.586±0.170d	0.016±0.007bcd	0.736±0.306a	0.024±0.008a
	KIO_3+V	0.25±0.02a	0.043±0.012c	0.005±0.001a	0.013±0.003abc	1.012±0.079b	0.026±0.004a
	KIO_3+SA+V	0.17±0.01a	0.012±0.001a	0.011±0.003ab	0.009±0.004ab	0.777±0.151ab	0.003±0.001a
	5-ISA+V	0.19±0.01a	0.122±0.005d	0.009±0.002ab	0.013±0.003abc	0.655±0.126a	0.010±0.004a
	3,5-diISA+V	0.19±0.01a	0.017±0.003ab	0.210±0.013c	0.011±0.001abc	0.790±0.220ab	0.015±0.006a

Means in the column followed by different letters separately for each experiments differ significantly at $P < 0.05$ (n=8).

Table S5 Concentrations of benzoic acid (BeA), 2-iodobenzoic acid (2-IBeA), 4-iodobenzoic acid (4-IBeA) and 2,3,5-triiodobenzoic acid (2,3,5-triIBeA) in leaves /head/ and roots of lettuce cultivated in hydroponics NFT Experiment No. 1 as well as in leaves of plants cultivated in pot Experiment No. 2 and 3

Experiment No. / Part of plant	Treatments	(mg·kg^{-1} D.W.)			
		BeA	2-IBeA	4-IBeA	2,3,5-triIBeA
1. Hydroponic NFT / Leaves	Control	2.54±0.19ab	0.081±0.009bc	0.024±0.009ab	0.005±0.001a
	SA	1.69±0.44ab	0.055±0.002abc	0.025±0.009ab	0.005±0.001a
	KIO_3	3.81±1.48bc	0.084±0.009c	0.017±0.007a	0.009±0.002a
	KIO_3+SA	3.60±0.96bc	0.023±0.002abc	0.025±0.008ab	0.009±0.001a
	5-ISA	2.20±0.38ab	0.022±0.002abc	0.026±0.009ab	0.007±0.001a
	3,5-diISA	3.82±0.84bc	0.044±0.003abc	0.029±0.009b	0.008±0.002a
	KIO_3+V	3.29±1.45abc	0.014±0.001a	0.022±0.007ab	0.005±0.001a
	KIO_3+SA+V	3.41±0.65abc	0.018±0.002ab	0.020±0.007ab	0.008±0.001a
	5-ISA+V	5.67±1.24c	0.031±0.002abc	0.022±0.008ab	0.008±0.002a
	3,5-diISA+V	0.94±0.16a	0.024±0.001abc	0.021±0.007ab	0.006±0.001a
1. Hydroponic NFT / Roots	Control	3.53±0.60a	0.039±0.001a	0.035±0.006b	0.007±0.001a
	SA	3.33±0.56a	0.029±0.002a	0.020±0.002ab	0.010±0.003a
	KIO_3	3.87±1.27a	0.039±0.002a	0.086±0.006d	0.006±0.002a
	KIO_3+SA	4.42±0.66a	0.034±0.003a	0.040±0.003bc	0.008±0.001a
	5-ISA	3.44±0.47a	0.093±0.007b	0.039±0.004bc	0.008±0.001a
	3,5-diISA	6.87±2.03ab	0.039±0.009a	0.021±0.004ab	0.209±0.046c
	KIO_3+V	12.45±7.75bc	0.032±0.003a	0.032±0.006b	0.002±0.001a
	KIO_3+SA+V	5.85±1.48ab	0.038±0.005a	0.031±0.006b	0.006±0.002a
	5-ISA+V	14.23±3.64c	0.151±0.044c	0.058±0.004c	0.003±0.001a
	3,5-diISA+V	6.48±1.04ab	0.038±0.003a	0.001±0.000a	0.189±0.034b
2. Peat substrate / Leaves	Control	3.56±0.39a	0.017±0.004a	0.0010±0.0003a	0.004±0.001a
	SA	2.22±0.36a	0.012±0.002a	0.0021±0.0009ab	0.004±0.000a
	KIO_3	2.47±0.54a	0.013±0.001a	0.0021±0.0004ab	0.005±0.001ab
	KIO_3+SA	1.80±0.46a	0.027±0.004a	0.0029±0.0007ab	0.007±0.002ab
	5-ISA	3.09±0.41a	0.011±0.002a	0.0028±0.0008ab	0.005±0.001ab
	3,5-diISA	2.12±0.42a	0.022±0.004a	0.0011±0.0004a	0.007±0.001ab
	KIO_3+V	2.94±0.74a	0.020±0.006a	0.0015±0.0006a	0.014±0.006b
	KIO_3+SA+V	2.51±0.54a	0.012±0.002a	0.0068±0.0024c	0.004±0.001a
	5-ISA+V	3.03±0.79a	0.025±0.008a	0.0019±0.0005a	0.006±0.001ab
	3,5-diISA+V	2.51±0.43a	0.022±0.004a	0.0031±0.0005bc	0.005±0.001ab
3. Mineral soil / Leaves	Control	5.03±1.46ab	0.151±0.046e	0.0040±0.0009ab	0.019±0.006bc
	SA	4.18±1.17ab	0.098±0.030d	0.0038±0.0008ab	0.021±0.006c
	KIO_3	9.52±2.53c	0.162±0.052e	0.0040±0.0013ab	0.014±0.004bc
	KIO_3+SA	3.46±1.69ab	0.082±0.026cd	0.0019±0.0007a	0.005±0.002a
	5-ISA	4.61±1.36a	0.041±0.009ab	0.0025±0.0006ab	0.010±0.002ab
	3,5-diISA	5.97±1.11b	0.048±0.010abc	0.0305±0.0099e	0.067±0.026d
	KIO_3+V	2.82±0.48a	0.033±0.009ab	0.0144±0.0027c	0.005±0.001a
	KIO_3+SA+V	2.90±0.64ab	0.017±0.004a	0.0200±0.0024d	0.006±0.001a
	5-ISA+V	2.30±0.73a	0.018±0.004a	0.0110±0.0020c	0.006±0.001a
	3,5-diISA+V	2.58±0.47a	0.067±0.013bcd	0.0044±0.0013ab	0.005±0.001a

Means in the column followed by different letters separately for each experiments differ significantly at $P < 0.05$ (n=8).

Content of I and V in soil after lettuce cultivation in both pot experiments

Both in control and in the group with SA application, the content of iodine in peat substrate and mineral soil was lower after lettuce cultivation than before the cultivation (Table S6). On the other hand, in the remaining combinations that used iodine fertilization of plants, iodine content in the substrate after cultivation was higher than before the cultivation. This indicates that plants did not take up all of the iodine applied. In both pot experiments with lettuce, the highest iodine content in substrate after harvesting was reported for 3,5-diISA and 3,5-diISA+V. In the combinations with KIO_3+V, KIO_3+SA+V, and 5-ISA+V in peat substrate (Experiment 2), the content of iodine in the substrate following lettuce harvesting was significantly lower than when these compounds were used without added vanadium.

The substrate content of vanadium was similar in all combinations analyzed in both experiments (Table S6).

Tabele S6. Iodine and vanadium content in soil prior to the experiment (average values) as well as after lettuce cultivation in pot Experiments No. 2 and 3 separately for each treatment.

Treatments	Peat substrate Experiment No. 2		Mineral soil Experiment No. 3	
	Iodine (mg I·kg^{-1})	Vanadium (mg V·kg^{-1})	Iodine (mg I·kg^{-1})	Vanadium (mg V·kg^{-1})
Prior to the lettuce cultivation	6.25±0.36	0.84±0.27	5.56±0.41	6.57±0.07
Control	5.3±0.88a	0.77±0.13a	4.8±0.15a	6.01±0.32a
SA	5.1±0.98a	0.70±0.14a	4.9±0.05a	5.95±0.16a
KIO_3	8.1±1.32c	0.70±0.14a	5.0±0.20ab	6.03±0.23a
KIO_3+SA	8.8±1.69c	0.75±0.12a	5.4±0.07bc	6.17±0.26a
5-ISA	8.3±1.73c	0.69±0.13a	5.5±0.13c	6.06±0.20a
3,5-diISA	13.5±3.18d	0.77±0.11a	6.6±0.27d	6.13±0.21a
KIO_3+V	7.4±1.34b	0.65±0.14a	5.3±0.15bc	6.03±0.24a
KIO_3+SA+V	7.8±1.40b	0.70±0.09a	5.3±0.11bc	6.04±0.25a
5-ISA+V	7.8±1.44b	0.67±0.12a	5.5±0.11c	6.10±0.21a
3,5-diISA+V	12.4±1.70d	0.60±0.10a	6.2±0.41d	6.03±0.20a

Means in the column followed by different letters separately for each experiments differ significantly at $P < 0.05$ (n=8).

Supplementary data S1. The functional annotation of *per12-like i per64-like* genes

The BLASTx search on NCBI (http://www.ncbi.nlm.nih.gov) against the non-redundant (nr) protein database, default patrameters showed that *per12-like* gene of *Lactuca sativa* L. var. *capitata* 'Melodion' c.v. (Acc. No. MT649253) has 100% identity to *L. sativa* peroxidase 12-like (Acc. No. XP_023743266.1), 85% identity to *C. cardunculus*_var._*scolymus* peroxidase 12-like (Acc. No. XP_024976944.1), 69% to *H. syriacus* peroxidase 12 (Acc. No. KAE8668076.1) and 62% to *A. thaliana* peroxidase 12 (Acc. No. NP_177313.1, UniProtKB database: Q96520).

The BLASTx search showed that *per64-like* gene of *Lactuca sativa* L. var. *capitata* 'Melodion' c.v. (Acc. No. MT649254) has 100% identity to *L. sativa* peroxidase 64-like (Acc. No. XP_023755436.1), 85% identity to *C. cardunculus*_var._*scolymus* peroxidase 64-like (Acc. No. XP_024970840.1), 80% to *V. vinifera* peroxidase 64 (Acc. no. RVX16101.1) and 72% to *A. thaliana p*eroxidase 64 (Acc. no. NP_199033.1, UniProtKB database: Q43872).

Comparative analysis of *A. thaliana* PER-12 and PER-64 proteins well annotated in the UniProtKB/Swiss-Prot (reviewed) are presented below. PER-12 and PER-64 share the same domains, only the PS00436 motif was identified in PER-12.

	***Arabidopsis thaliana* PER-12**	***Arabidopsis thaliana* PER-64**
UniProtKB	Q96520 (PER12_ARATH)	Q43872 (PER64_ARATH)
Protein name	Peroxidase 12 (EC:1.11.1.7)<br>Alternative name(s): ATP4a, PRXR6	Peroxidase 64 (EC:1.11.1.7)<br>Alternative name(s): ATP17a, PRXR4
Sequence length (aa)<br>Mass (kDa)	358<br>39.559	317<br>34.706
Catalytic activity	2 a phenolic donor + H_2O_2 = 2 a phenolic radical donor + 2 H_2O<br>(EC:1.11.1.7)	2 a phenolic donor + H_2O_2 = 2 a phenolic radical donor + 2 H_2O<br>(EC:1.11.1.7)
Function	Removal of H_2O_2, oxidation of toxic reductants, biosynthesis and degradation of lignin, suberization, auxin catabolism, response to environmental stresses such as wounding, pathogen attack and oxidative stress. These functions might be dependent on each isozyme/isoform in each plant tissue. Exhibits a Ca^{2+}-pectate binding affinity which could be interpreted *in vivo* as a specificity to interact with the pectic structure of the cell wall.	Removal of H_2O_2, oxidation of toxic reductants, biosynthesis and degradation of lignin, suberization, auxin catabolism, response to environmental stresses such as wounding, pathogen attack and oxidative stress. These functions might be dependent on each isozyme/isoform in each plant tissue.
Enzyme and pathway databases	BioCyc: ARA:AT1G71695-MONOMER	BioCyc: ARA:AT5G42180-MONOMER
Subcellular location	Vacuole<br>Extracellular region or secreted	Extracellular region or secreted
Cofactor	heme *b*<br>Binds 1 heme b (iron(II)-protoporphyrin IX) group per subunit.<br>Ca^{2+}<br>Binds 2 calcium ions per subunit.	heme *b*<br>Binds 1 heme b (iron(II)-protoporphyrin IX) group per subunit.<br>Ca^{2+}<br>Binds 2 calcium ions per subunit.
Sites	Transition state stabilizer: 80 (R)<br>Active site: Proton akceptor: 84 (H)<br>Metal binding:<br>Calcium 1: 85 (D), 92 (E), 94 (S)<br>Calcium 1 via carbonyl oxygen: 88 (V), 90 (G)<br>Calcium 2: 214 (T), 259 (D), 262 (S), 267 (D)	Transition state stabilizer: 59 (R)<br>Active site: Proton akceptor: 63 (H)<br>Metal binding:<br>Calcium 1: 64 (D), 71 (D),73 (S)<br>Calcium 1 via carbonyl oxygen: 67(V), 69 (G)<br>Calcium 2: 189 (T), 241 (D), 243 (T), 248 (D)

	Iron (heme axial ligand): 213 (H)<br>Binding site:<br>Substrate; via carbonyl oxygen: 183 (P)	Iron (heme axial ligand): 188 (H)<br>Binding site:<br>Substrate; via carbonyl oxygen: 158 (P)
Molecule processing	Signal peptide: 1-31<br>Peroxidase 12: 32-358	Signal peptide: 1-22<br>Peroxidase 64: 23-317
Amino acid modifications	Disulfide bond: 53↔134, 86↔91, 140↔335, 220↔247<br>Glycosylation: N-linked (GlcNAc...) asparagine: 188, 202, 251, 334	Disulfide bond: 32↔111, 65↔70, 117↔313, 195↔227<br>Glycosylation: N-linked (GlcNAc...) asparagine: 163
Expression	Tissue specificity:<br>Expressed in roots and leaves.<br>Developmental stage<br>Expressed in the first stage of developing seeds.<br>Induction<br>Induced either by incompatible fungal pathogen attack, or by methyl jasmonate, a plant defense-related signaling molecule.	Tissue specificity:<br>Expressed in the whole plant, but preferentially in roots.<br><br>Induction<br>Pathogen and elicitor-induced. Up-regulated transiently by a cold treatment.
Protein-protein interaction databases	STRING: 3702.AT1G71695.1	STRING: 3702.AT5G42180.1
3D structure databases	SMR: Q96520<br>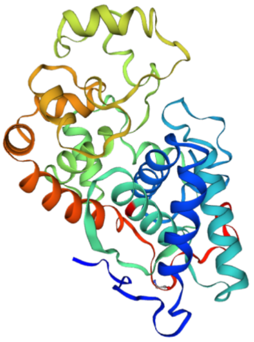	SMR: Q43872<br>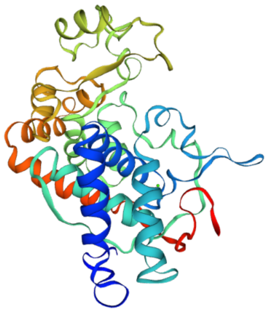
Family & Domains	Belongs to the peroxidase family. Classical plant (class III) peroxidase subfamily.<br><br>**InterPro**:<br>IPR002016 Haem_peroxidase (43-339 aa)<br>IPR010255 Haem_peroxidase_sf (39-339 aa)<br>IPR000823 Peroxidase_pln (53-72, 77-97, 118-131, 137-147, 156-171, 204-216, 257-290, 313-326 aa)<br>IPR019794 Peroxidases_AS (75-86 aa) (AAAILRIHFHDC)<br>IPR019793 Peroxidases_heam-ligand_BS (205-215 aa) (DLVALSGGHTI)<br>IPR033905 Secretory_peroxidase (43-337 aa)<br>**Pfam**:<br>PF00141 peroxidase, 1 hit (60-303 aa)<br>**Prints:**<br>PR00458 PEROXIDASE<br>PR00461 PLPEROXIDASE<br>**Prosite:**<br>PS00435 PEROXIDASE_1, 1 hit (205-215 aa) (DLVALSGGHTI)<br>PS00436 PEROXIDASE_2, 1 hit (75-86 aa) (AAaiLRIhFHDC), Active site: 84 (H)<br>PS50873 PEROXIDASE_4, 1 hit (43-339 aa)	**CDD:**<br>cd00693 Secretory_peroxidase, 1 hit (23-316 aa)<br>**InterPro**:<br>IPR002016 Haem_peroxidase (22-317 aa)<br>IPR010255 Haem_peroxidase_sf (23-316 aa)<br>IPR000823 Peroxidase_pln (32-51, 56-76, 95-108, 114-124, 133-148, 179-191, 238-271, 294-307 aa)<br><br>IPR019793 Peroxidases_heam-ligand_BS (180-190 aa) (DLVALSGGHTL)<br>IPR033905 Secretory_peroxidase (23-316 aa)<br>**Pfam**:<br>PF00141 peroxidase, 1 hit (39-284 aa)<br>**Prints:**<br>PR00458 PEROXIDASE<br>PR00461 PLPEROXIDASE<br>**Prosite:**<br>PS00435 PEROXIDASE_1, 1 hit (180-190 aa) (DLVALSGGHTL)<br><br>PS50873 PEROXIDASE_4, 1 hit (22-317 aa)<br>Supfam:<br>SSF48113 SSF48113, 1 hit

The full-length of *L. sativa* peroxidase 12-like (XP_023743266.1) and peroxidase 64-like (XP_023755436.1) and also *A. thaliana* peroxidase 12 (Q96520) and peroxidase 64 (Q43872) were aligned using Clustal Omega ver. 1.2.4 (https://www.ebi.ac.uk/Tools/msa/clustalo). Localization of haem peroxidase domain (IPR002016) and conserved sites and regions were highlighted using different colours on the alignignment enclosed below i.e. active site and substrate bindig site, transition state stabilizer, Ca and Fe banding site.

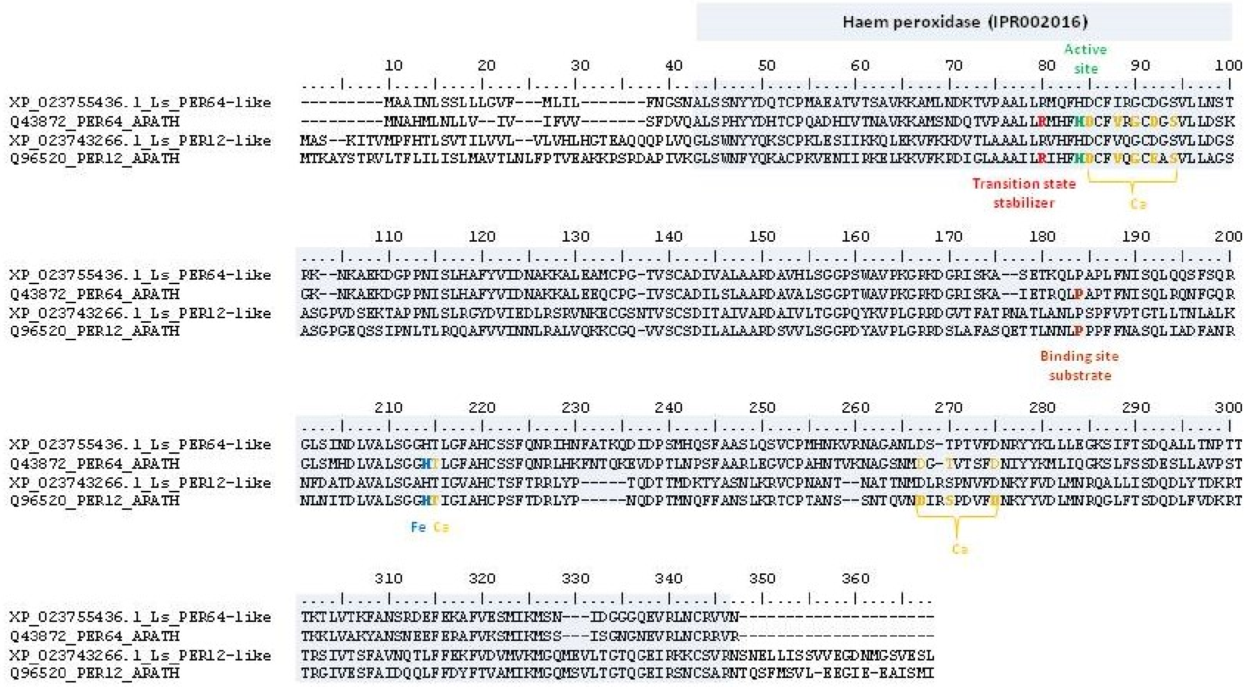

Comperative analysis of seaweed vanadium-dependent bromoperoxidase (v-BPO) at *C. officinalis* (Q8LLW7) and *A. nodosum* (P81701) and vanadium-dependent iodoperoxidase 1 (vIPO1) at *L. digitata* (Q4LDE6) are presented below.

	Corallina officinalis (Coral seaweed)	***Ascophyllum nodosum (Brown seaweed)***	***Laminaria digitata***
UniProtKB	Q8LLW7 (PRXV_COROI)	P81701 (PRXV_ASCNO)	Q4LDE6 (Q4LDE6_9PHAE)
Protein name	Vanadium-dependent bromoperoxidase V-BPO (EC:1.11.1.18) Alternative name(s): Vanadium haloperoxidase	Vanadium-dependent bromoperoxidase V-BPO (EC:1.11.1.18) Alternative name(s): Vanadium haloperoxidase	Vanadium-dependent iodoperoxidase 1 vIPO1
Sequence length (aa)<br>Mass (kDa)	598<br>65.459	557<br>60.344	624<br>66.326
Catalytic activity	RH + HBr + H_2O_2 = RBr + 2 H_2O (EC:1.11.1.18)	RH + HBr + H_2O_2 = RBr + 2 H_2O (EC:1.11.1.18)	-
Function	Catalyzes the halogenation of organic substrates in the presence of hydrogen peroxide. <u>GO - Molecular function:</u> peroxidase activity	Catalyzes the halogenation of organic substrates in the presence of hydrogen peroxide <u>GO - Molecular function:</u> peroxidase activity	<u>GO - Molecular function:</u> peroxidase activity oxidoreductase peroxidase
Enzyme & pathway	BRENDA: 1.11.1.18 1611	BRENDA: 1.11.1.18 8891	-

databases			
Cofactor	Vanade<br>Binds 1 vanadate ion per subunit.<br>Ca^{2+}<br>Binds 1 Ca^{2+} ion per subunit.<br>The binding is important for enzyme stability.	Vanade<br>Binds 1 vanadate ion per subunit.	-
Sites & regions	Binding site:<br>Vanade: 400 (K), 408 (R), 547 (R), 553 (H)<br>Active site:<br>480 (H), 487 (H)	Binding site:<br>Vanade: 341 (K), 349 (R), 480 (R), 486 (H)<br>Active site:<br>411 (H), 418 (H)<br>Region:<br>Vanadate binding: 416-418 aa (SGH)	Active site:<br>403 (K), 411(R), 481 (A), 482 (G), 549 (R), 555 (H), 559 (D)
Family & Domains	Belongs to the bacterial non-heme bromo- and chloro-peroxidases family.<br>**Gene3D:**<br>1.10.606.10, Acid phosphatase/Vanadium-dependent haloperoxidase superfamily, 1 hit<br>(1-598 aa)<br>**InterPro:**<br>IPR016119<br>Br/Cl_peroxidase_C<br>(1-598 aa)<br>IPR036938<br>P_Acid_Pase_2/haloperoxi_sf<br>(3-597 aa)<br>IPR000326<br>Phosphatidic acid phosphatase type 2/haloperoxidase<br>(472-571 aa)<br>**Pfam:**<br>PF01569 PAP2, 1 hit<br>(472-571 aa)<br>**Supfam:**<br>SSF48317 SSF48317, Acid phosphatase/Vanadium-dependent haloperoxidase superfamily, 1 hit<br>(3-597 aa)	Belongs to the bacterial non-heme bromo- and chloro-peroxidases family<br>**Gene3D:**<br>1.10.606.10, Acid phosphatase/Vanadium-dependent haloperoxidase superfamily, 1 hit<br>(1-556 aa)<br>**InterPro:**<br>IPR016119<br>Br/Cl_peroxidase_C<br>(1-556 aa)<br>IPR036938<br>P_Acid_Pase_2/haloperoxi_sf<br>(3-553 aa)<br>**Supfam:**<br>SSF48317 SSF48317, Acid phosphatase/Vanadium-dependent haloperoxidase superfamily, 1 hit<br>(3-553 aa)<br>**CDD:**<br>cd03398, PAP2, haloperoxidase_like subfamily<br>(257-507 aa)	**Gene3D:**<br>1.10.606.10, Acid phosphatase/Vanadium-dependent haloperoxidase superfamily, 1 hit<br>(61-624 aa)<br>**InterPro:**<br>IPR016119<br>Br/Cl_peroxidase_C<br>(61-624 aa)<br>IPR036938<br>P_Acid_Pase_2/haloperoxi_sf (74-599 aa)<br>**Supfam:**<br>SSF48317 SSF48317, Acid phosphatase/Vanadium-dependent haloperoxidase superfamily, 1 hit<br>**CDD:**<br>cd03398, PAP2, haloperoxidase_like subfamily (305-576 aa)
Molecule processing	Chain: Vanadium-dependent bromoperoxidase: 1-598 aa	Chain: Vanadium-dependent bromoperoxidase: 1-557 aa	Signal peptide: 1-25 aa<br>Chain: 26-624 aa
Amino acid modifications	Modified residue:<br>1 ↔ Pyrrolidone carboxylic acid<br>Disulfide bond:<br>3↔interchain C41, 41↔interchain C3, 77↔86, 441↔462, 544↔555	-	-

Protein-protein interaction databases	Homododecamer	Homodimer; disulfide-linked	-
3D structure databases	PDB: 1QHB<br>SMR: Q8LLW7<br>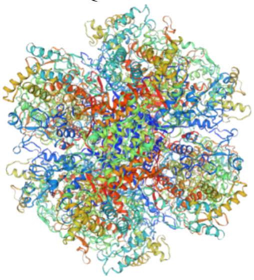	PDB: 1QI9<br>SMR: P81701<br>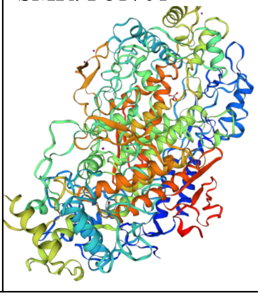	-

Results of pairwise alignment of protein sequences of *A. thaliana* PER12 and PER64 versus vIPO1 *L. digitata* and vBPO *C. officinalis* and *A. nodosum* are presented below.

	PER12_ARATH	PER64_ARATH
Laminaria digitata<br>Vanadium-dependent iodoperoxidase 1 (vIPO1)<br>Q4LDE6_9PHAE	Range:<br>PER12_ARATH: 1-358<br>Q4LDE6_9PHAE: 251-583<br><br>**Region: cd03398, PAP2, haloperoxidase_like subfamily (305-576 aa)**<br>**Region containing active site (403 – 559 aa)**<br>Identity: 11.2%, Similarity: 17.5%	Range:<br>PER64_ARATH: 67-317<br>Q4LDE6_9PHAE: 1-269<br><br>Identity: 8.3%, Similarity: 14.0%
Corallina officinalis<br>Vanadium-dependent bromoperoxidase (vBPO)<br>AAM46061.1 (Q8LLW7)	Range:<br>PER12_ARATH: 72-358<br>AAM46061.1: 1-349<br>Identity: 8.9%, Similarity:16.3%	Range:<br>PER64_ARATH: 31-317<br>AAM46061.1: 1-285<br>Identity: 9.8%, Similarity: 16.0%
Ascophyllum nodosum<br>Vanadium-dependent bromoperoxidase (vBPO)<br>PRXV_ASCNO	Range:<br>PER12_ARATH: 1-337<br>PRXV_ASCNO: 194-557<br><br>**Binding site: Vanade: 341 (K), 349 (R), 480 (R), 486 (H)**<br>**Active site: 411 (H),418 (H)**<br>**Region: cd03398, PAP2, haloperoxidase_like subfamily (257-507 aa)**<br>Identity:13.1%, Similarity:20.9%	Range:<br>PER64_ARATH: 1-119<br>PRXV_ASCNO: 469-557<br><br>**Binding site: Vanade: 480 (R), 486 (H)**<br><br>**Region: cd03398, PAP2, haloperoxidase_like subfamily (257-507 aa)**<br>Identity: 3.4%, Similarity:5.4%

Pairwise alignment using program Emboss Needle (https://www.ebi.ac.uk/Tools/psa/emboss_needle) with parameters: matrix BLOSUM62, gap open penalty 10.0, gap extend penalty 0.5, end open penalty 10.0, end extend penalty 0.5.

Supplementary data S2. The functional annotation of *cipk6* gene

The BLASTx search on NCBI (http://www.ncbi.nlm.nih.gov) againt the non-redundant (nr) protein database, default patrameters showed that *cipk6* gene of *Lactuca sativa* L. var. *capitata* 'Melodion' c.v. (Acc. No. MT663549) has 100% identity to *L. sativa* CBL-interacting serine/threonine-protein kinase 6-like (Acc. No. XP_023745436.1, UniProtKB database: A0A2J6JQA9_LACSA), 86% identity to *H. annuus* CBL-interacting serine/threonine-protein kinase 6-like (Acc. No. XP_022038504, UniProtKB database: A0A251UQA7_HELAN and 72% identity to *A. thaliana* CBL-interacting serine/threonine-protein kinase 6 (Acc. No. NP_194825.1, UniProtKB database: O65554). Comparative analysis of CIPK6 proteins of *L. sativa*, *H.annus* and *A. thaliana* are presented below.

	Lactuca sativa	***Helianthus annuus***	***Arabidopsis thaliana***
UniProtKB	A0A2J6JQA9<br>(A0A2J6JQA9_LACSA)	A0A251UQA7<br>(A0A251UQA7_HELAN)	O65554<br>(CIPK6_ARATH)
Protein name	Non-specific serine/threonine protein kinase<br>(EC:2.7.11.1)	Non-specific serine/threonine protein kinase<br>(EC:2.7.11.1)<br>CIPK6	CBL-interacting serine /threonine-protein kinase 6<br>(EC:2.7.11.1)<br>CIPK6<br>Alternative name(s):<br>SNF1-related kinase 3.14<br>SOS2-like protein kinase PKS4<br>SOS3-interacting protein 3
Sequence length (aa)<br>Mass (kDa)	428<br><br>47.731	426<br><br>47.548	441<br><br>49.357
Catalytic activity	ATP + L-seryl-[protein] = ADP + H^+ + *O*-phospho-L-seryl-[protein]<br>(EC:2.7.11.1)<br>ATP + L-threonyl-[protein] = ADP + H^+ + *O*-phospho-L-threonyl-[protein]<br>(EC:2.7.11.1)	ATP + L-seryl-[protein] = ADP + H^+ + *O*-phospho-L-seryl-[protein]<br>(EC:2.7.11.1)<br>ATP + L-threonyl-[protein] = ADP + H^+ + *O*-phospho-L-threonyl-[protein]<br>(EC:2.7.11.1)	ATP + L-seryl-[protein] = ADP + H^+ + *O*-phospho-L-seryl-[protein]<br>(EC:2.7.11.1)<br>ATP + L-threonyl-[protein] = ADP + H^+ + *O*-phospho-L-threonyl-[protein]<br>(EC:2.7.11.1)
Function	GO - Molecular function:<br>ATP binding<br>protein serine/threonine kinase activity<br>GO - Biological process:<br>signal transduction	GO - Molecular function:<br>ATP binding<br>protein serine/threonine kinase activity<br>GO - Biological process:<br>signal transduction	GO - Molecular function:<br>ATP binding<br>protein serine/threonine kinase activity<br>GO - Biological process:<br>basipetal auxin transport<br>hyperosmotic salinity response<br>multicellular organism development<br>response to salt stress<br>response to water deprivation<br>signal transduction<br>GO - Cellular component:<br>endoplasmic reticulum<br>Targeted to the cell membrane when interacting with CBL4 and ATK2.<br>CIPK serine-threonine protein kinases interact with CBL proteins. Binding of a CBL

			protein to the regulatory NAF domain of CIPK protein lead to the activation of the kinase in a calcium-dependent manner. Downstream of CBL1, CBL2, CBL3 and CBL9, regulates by phosphorylation the K+ conductance and uptake of AKT1. Binds to CBL4 to modulate AKT2 activity by promoting a kinase interaction-dependent but phosphorylation-independent translocation of the channel to the plasma membrane.
Cofactor	-	-	Mn^{2+}
Sites			Binding site:<br>ATP: 53 (K)<br>Active site:<br>Proton akceptor: 146 (D)<br>Regions:<br>Nucleotide binding: ATP: 30-38 aa (LGHGTFAKV)
PTM / Processing	-	-	Amino acid modifications:<br>168 (Phosphoserine)<br>182 (Phosphothreonine)
Expression	-	-	Expressed in roots and shoots.
Interaction	-	-	Part of a K^{+}-channel calcium-sensing kinase/phosphatase complex composed by a calcium sensor CBL (CBL1, CBL2, CBL3 or CBL9), a kinase CIPK (CIPK6, CIPK16 or CIPK23), a phosphatase PP2C (AIP1) and a K^{+}-channel (AKT1).<br>Interacts with AKT1, AKT2,CBL1, CBL2, CBL3, CBL4/SOS3 and CBL9.
Family & Domains	Belongs to the protein kinase superfamily.<br><br>**InterPro**:<br>IPR011009 Kinase-like_dom_sf (11-276 aa)<br>IPR018451 NAF/FISL_domain (291-315 aa)<br>IPR004041 NAF_dom (294-353 aa)	Belongs to the protein kinase superfamily.<br><br>**InterPro**:<br>IPR011009 Kinase-like_dom_sf (11-276 aa)<br>IPR018451 NAF/FISL_domain (289-313 aa)<br>IPR004041 NAF_dom (292-351 aa)	The activation loop within the kinase domain is the target of phosphorylation/activation by upstream protein kinases. The PPI motif mediates the interaction with the ABI (abscisic acid-insensitive) phosphatases.<br>**Regions:**<br>Activation loop: (164 -193 aa)<br>PPI: (341- 371 aa)<br>**InterPro**:<br>IPR011009 Kinase-like_dom_sf (20-290 aa)<br>IPR018451 NAF/FISL_domain (310-334 aa)<br>IPR004041 NAF_dom (313-373 aa)

	IPR000719 Prot_kinase_dom (15-269 aa)<br>IPR017441 Protein_kinase_ATP_Binding Site (21-48 aa) (LGHGTFAKVYHARNLQT GKSVAMKMVAK)<br>IPR008271 Ser/Thr_kinase_Active Site (133-145 aa) (VYHRDLKPENLLL)<br>**Pfam**:<br>PF03822 NAF (293-353 aa)<br>PF00069 Pkinase (15-269 aa)<br>**Supfam:**<br>SSF56112 SSF56112<br>**Prosite:**<br>PS50011 PROTEIN_KINASE_DOM (15-269 aa)<br>ATP Binding: 21-29 aa (LGHGTFAKV) and 44 aa (K)<br>Active site: proton acceptor: 137 aa (D)<br>PS50816 NAF: 291-315 aa (KEGESLNAFHIISLSEGFD LSPLFE)<br>PS00107 PROTEIN_KINASE_ATP-binding region signature: 21-48 aa (LGHGTFAKVYhArnlqtgksv amk......MVAK)<br>PS00108 PROTEIN_KINASE_ctive-site signature: 133-145 aa (VyHrDLKpeNLLL), active site: 137 aa (D)	IPR000719 Prot_kinase_dom (15-269 aa)<br>IPR017441 Protein_kinase_ATP_Binding Site (21-48 aa) (LGHGTFAKVYHARNLKT GKSVAMKMVGK)<br>IPR008271 Ser/Thr_kinase_Active Site (133-145 aa) (VYHRDLKPENLLL)<br>**Pfam**:<br>PF03822 NAF (291-351 aa)<br>PF00069 Pkinase (15-269 aa)<br>**Supfam:**<br>SSF56112 SSF56112<br>**Prosite:**<br>PS50011 PROTEIN_KINASE_DOM (15-269 aa)<br>ATP Binding: 21-29 aa (LGHGTFAKV) and 44 aa (K)<br>Active site: proton acceptor: 137 aa (D)<br>PS50816 NAF: 289-313 aa (TGKECLNAFHIISLSEGFD LSPLFE)<br>PS00107 PROTEIN_KINASE_ATP-binding region signature: 21-48 aa (LGHGTFAKVYhArnlktgksv amk......MVGK)<br>PS00108 PROTEIN_KINASE_ctive-site signature (133-145 aa) (VyHrDLKpeNLLL) active site: 137 aa (D)	IPR000719 Prot_kinase_dom (24-278 aa)<br>IPR017441 Protein_kinase_ATP_Binding Site (30-57 aa) (LGHGTFAKVYHARNIQT GKSVAMKVVGK)<br>IPR008271 Ser/Thr_kinase_Active Site (142-154 aa) (VYHRDLKPENLLL)<br>**Pfam**:<br>PF03822 NAF (312-373 aa)<br>PF00069 Pkinase (24-278 aa)<br>**Supfam:**<br>SSF56112 SSF56112<br>**Prosite:**<br>PS50011 PROTEIN_KINASE_DOM (24-278 aa)<br>ATP Binding: 30-38 aa (LGHGTFAKV) and 53 aa (K)<br>Active site: proton acceptor: 146 aa (D)<br>PS50816 NAF: 310-334 aa (EETETLNAFHIIALSEGFD LSPLFE)<br>PS00107 PROTEIN_KINASE_ATP-binding region signature: 30-57 aa (LGHGTFAKVYhArniqtgksv amk......VVGK)<br>PS00108 PROTEIN_KINASE_active-site signature (142-154 aa) (VyHrDLKpeNLLL)
3D structure databases	-	-	SMR:<br>O65554<br>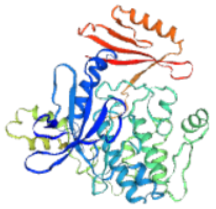

The full-length CBL-interacting serine/threonine-protein kinase 6-like of *L.sativa* (A0A2J6JQA9), *H. annuus* (A0A251UQA7) and *A. thaliana* (O65554) were aligned using Clustal Omega ver. 1.2.4 (https://www.ebi.ac.uk/Tools/msa/clustalo). Localization of protein kinase domain (IPR000719) and NAF domain (IPR004041) was indicated on the alignignment enclosed below.

```
                          Protein kinase domain (IPR000719)
                         10        20        30        40        50        60        70        80        90       100
O65554|CIPK6_ARATH   MVGAKPVENGSDGGSSTGLLHGRYELGRLLGHGTFAKVYHARNIQTGKSVAMKVVGKEKVVKVGMVDQIKREISVMRMVKHPNIVELHEVMASKSKIYFA
A0A2J6JQA9_LACSA     ---------MAMEDRNNGVLHGKYELGKQLGHGTFAKVYHARNLQTGKSVAMKMVAKDKVIKVGMTDHIQREISVMKMVRHPNIVELHEVMASKSKIYFA
A0A251UQA7_HELAN     ---------MATEERNNGVLLGKYELGRQLGHGTFAKVYHARNLKTGKSVAMKMVGKEKVIKVGMTDQIQREIAVMKMVKHPNIVELHEVMASKSNIYFA

                        110       120       130       140       150       160       170       180       190       200
O65554|CIPK6_ARATH   MELVRGGELFAKVAKGRLREDVARVYFQQLISAVDFCHSRGVYHRDLKPENLLLDEEGNLKVTDFGLSAFTEHLKQDGLLHTTCGTPAYVAPEVILKKGY
A0A2J6JQA9_LACSA     MELVRGGELFSKVEKGRLREDVARSYFQQLISAVDFCHSRGVYHRDLKPENLLLDGEGNLKVTDFGLSALSDHLRQDGLLHTTCGTPAYVAPEVIGKKGY
A0A251UQA7_HELAN     MELVRGGELFSKIAKGRLPEDVARSYFQQLISAVDFCHSRGVYHRDLKPENLLLDEEGNLKVTDFGLSAFSDHIRQDGLLHTTCGTPAYVAPEVIGKKGY

                        210       220       230       240       250       260       270       280       290       300
O65554|CIPK6_ARATH   DGAKADLWSCGVILFVLLAGYLPFQDDNLVNMYRKIYRGDFKCPGWLSSDARRLVTKLLDPNPNTRITIEKVMDSPWFKKQATRSRNEPVAATITTTEED
A0A2J6JQA9_LACSA     SGAQADIWSCGVILYVLLAGFLPFQDENIVAMYRKIYKGDFKCPPWFSSDARRLIVKLLDPNPKSRITISKIMQSTWFKKATPKKPNFSGE---------
A0A251UQA7_HELAN     DGAKADIWSCGVILYVLLAGFLPFQDDNLIAMYKKIYRGDFKCPPWFSTDARRLITKMLDTKPSTRITIAQVMDSPWFKKAAPKKPNFSFD---------

                                   NAF domain (IPR004041)
                        310       320       330       340       350       360       370       380       390       400
O65554|CIPK6_ARATH   VDFLVHKSKEETETLNAFHIIALSEGFDLSPLFEEKKKEEKREMRFATSRPASSVISSLEEAARVGNKFDVRKS--ESRVRIEGKQNGRKGKLAVEAEIF
A0A2J6JQA9_LACSA     -DEGMHLKGKEGESLNAFHIISLSEGFDLSPLFEEKKREEKQEIRFATMKSAGAVVSKLEEVAKA-VKFSVKKSGDESSLRLQGLENGRKGKLGIAADIF
A0A251UQA7_HELAN     -DESANL--TGKECLNAFHIISLSEGFDLSPLFEEKKREEKQEIRFATMKPVTAVVSKLEAVAKA-VKFSVKKSGDESSLRLQGHENGRKGKLGISADIF

                        410       420       430       440
O65554|CIPK6_ARATH   AVAPSFVVVEVKKDHGDTLEYNNFCSTALRPALKDIFWTSTPA-----
60A0A2J6JQA9_LACSA   AVTPSFLVVEVTKSSGDTLEYNQFCSKELRPALKDIVWTSPANHSTPA
51A0A251UQA7_HELAN   AVTSSFLVVEVKKSSGDTLEYDQFCSKELRPALKDIVWTSPADNSTQA
```

The protein sequence logo (http://weblogo.berkeley.edu) (presented below) representing the alignment of *L.sativa* (A0A2J6JQA9), *H. annuus* (A0A251UQA7) and *A. thaliana* (O65554) confirmed that active and binding sites and nucleotide binding ATP region are conserved but less identical aminoacids were found within the NAF and PPI motifs.

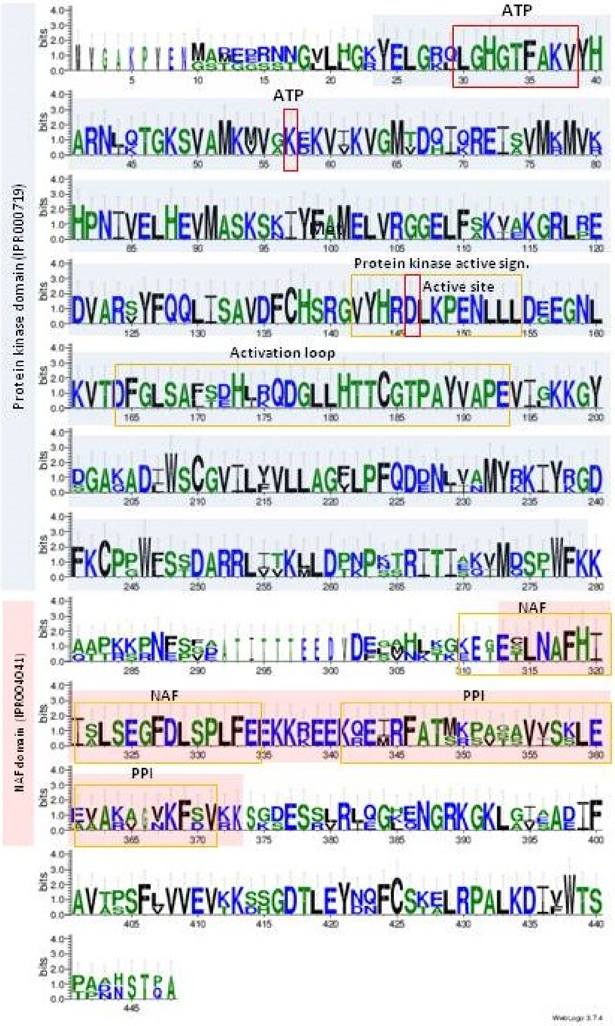

Supplementary data S3. The functional annotation of *msams5* gene

The BLASTx search on NCBI (http://www.ncbi.nlm.nih.gov) against the non-redundant (nr) protein database, default patrameters showed that *msams5* gene of *Lactuca sativa* L. var. *capitata* 'Melodion' c.v. (Acc. No. MT663551) has 100% identity to *L. sativa* S-adenosylmethionine synthase 5 (Acc. No. XP_023765742.1, UniProtKB database: A0A2J6KI88_LACSA), 98% identity to *H. annuus* S-adenosylmethionine synthase 5 (Acc. No. XP_022038483.1, UniProtKB database: G3LUV1_HELAN) and 93% identity to *B. juncea* S-adenosylmethionine synthetase 5 (Acc. No. METK5_BRAJU, UniProtKB database: Q94FA4). Comparative analysis of S-adenosylmethionine synthase protein sequences of *L. sativa*, *H.annus* and *B. juncea* are presented below.

	Lactuca sativa	***Helianthus annuus***	***Brassica junce***
UniProtKB	A0A2J6KI88<br>(A0A2J6KI88_LACSA)	G3LUV1<br>(G3LUV1_HELAN)	Q94FA4<br>(METK5_BRAJU)
Protein name	S-adenosylmethionine synthase<br>(EC:2.5.1.6)	S-adenosylmethionine synthase<br>(EC:2.5.1.6)	S-adenosylmethionine synthase 5<br>(EC:2.5.1.6)<br>MSAMS5<br>Alternative name(s):<br>Methionine adenosyltransferase 5<br>MAT 5
Sequence length (aa)<br>Mass (kDa)	393<br><br>42.978	390<br><br>42.640	393<br><br>43.184
Catalytic activity	ATP + H_2O + L-methionine = diphosphate + phosphate + *S*-adenosyl-L-methionine<br>(EC:2.5.1.6)	ATP + H_2O + L-methionine = diphosphate + phosphate + *S*-adenosyl-L-methionine<br>(EC:2.5.1.6)	ATP + H_2O + L-methionine = diphosphate + phosphate + *S*-adenosyl-L-methionine<br>(EC:2.5.1.6)
Function	Catalyzes the formation of S-adenosylmethionine from methionine and ATP.<br>**Pathway:**<br>**S-adenosyl-L-methionine biosynthesis**<br>This protein is involved in step **1** of the subpathway that synthesizes S-adenosyl-L-methionine from L-methionine.<br>Proteins known to be involved in this subpathway in this organism are:<br>Step 1: S-adenosylmethionine synthase **(**LSAT_2X107260), S-adenosylmethionine synthase (LSAT_6X71400), **S**-adenosylmethionine synthase (LSAT_6X24520), S-adenosylmethionine synthase (LSAT_6X117861), S-adenosylmethionine synthase (LSAT_1X51641). This subpathway is part of the pathway S-adenosyl-L-	Catalyzes the formation of S-adenosylmethionine from methionine and ATP.<br>**Pathway:**<br>**S-adenosyl-L-methionine biosynthesis**<br>This protein is involved in step **1** of the subpathway that synthesizes S-adenosyl-L-methionine from L-methionine.<br>Proteins known to be involved in this subpathway in this organism are:<br>Step 1: S-adenosylmethionine synthase (HannXRQ_Chr14g0454811), S-adenosylmethionine synthase (METK3), S-adenosylmethionine synthase, S-adenosylmethionine synthase (HannXRQ_Chr13g0400841), S-adenosylmethionine synthase (METK2), S-adenosylmethionine synthase (METK3), S-adenosylmethionine synthase (SAMS1), S-adenosylmethionine synthase	Catalyzes the formation of S-adenosylmethionine from methionine and ATP. The reaction comprises two steps that are both catalyzed by the same enzyme:<br>• formation of S-adenosylmethionine (AdoMet) and triphosphate,<br>• subsequent hydrolysis of the triphosphate.<br>**Pathway:**<br>**S-adenosyl-L-methionine biosynthesis**<br>This protein is involved in step 1 of the subpathway that synthesizes S-adenosyl-L-methionine from L-methionine.<br>Proteins known to be involved in this subpathway in this organism are: S-adenosylmethionine synthase 5 (MSAMS5), S-adenosylmethionine synthase 4 (MSAMS4), S-adenosylmethionine

	methionine biosynthesis, which is itself part of Amino-acid biosynthesis.<br><br>GO - Molecular function:<br>ATP binding<br>metal ion binding<br>methionine adenosyltransferase activity<br>GO - Biological process:<br>one-carbon metabolic process<br>S-adenosylmethionine biosynthetic process<br><br>GO - Cellular component:<br>cytoplasm	(METK2), Methionine adenosyltransferase (HannXRQ_Chr01g0027791)<br>This subpathway is part of the pathway S-adenosyl-L-methionine biosynthesis, which is itself part of Amino-acid biosynthesis.<br>View all proteins of this organism that are known to be involved in the subpathway that synthesizes S-adenosyl-L-methionine from L-methionine, the pathway S-adenosyl-L-methionine biosynthesis and in Amino-acid biosynthesis.<br><br>GO - Molecular function:<br>ATP binding<br>metal ion binding<br>methionine adenosyltransferase activity<br>GO - Biological process:<br>one-carbon metabolic process<br>S-adenosylmethionine biosynthetic process<br><br>GO - Cellular component:<br>cytoplasm	synthase 1 (SAMS1), S-adenosylmethionine synthase 2 (MSAMS2), S-adenosylmethionine synthase 3 (MSAMS3)<br>This subpathway is part of the pathway S-adenosyl-L-methionine biosynthesis, which is itself part of Amino-acid biosynthesis.<br>View all proteins of this organism that are known to be involved in the subpathway that synthesizes S-adenosyl-L-methionine from L-methionine, the pathway S-adenosyl-L-methionine biosynthesis and in Amino-acid biosynthesis.<br>GO - Molecular function:<br>ATP binding<br>metal ion binding<br>methionine adenosyltransferase activity<br>GO - Biological process:<br>one-carbon metabolic process<br>S-adenosylmethionine biosynthetic process<br>GO - Cellular component:<br>cytoplasm

Cofactor	Protein has several cofactor binding sites:<br>• Mg^{2+}<br>• Co^{2+}	Protein has several cofactor binding sites:<br>• Mg^{2+}<br>• Co^{2+}	Protein has several cofactor binding sites:<br>• Mn^{2+}<br>• Mg^{2+}<br>• Co^{2+}<br>Binds 2 divalent ions per subunit. The metal ions interact primarily with the substrate. Can utilize magnesium, manganese or cobalt (in vitro)<br>• K^{+}<br>Binds 1 potassium ion per subunit. The potassium ion interacts primarily with the substrate
Sites	-	-	Binding site:<br>Mg: 9 (E)<br>K: 43 (E)<br>ATP: 15 (H), 246 (D), 269 (A), 273 (K), 277 (K)<br>Met: 56 (E), 99 (Q), 246 (D), 277 (K)<br>Regions:<br>Nucleotide binding: ATP: 167-169 aa (DGK), 235-238 aa (SGRF), 252-253 aa (RK)
Expression	-	-	Mostly expressed in flowers, seedpods and roots, and, to a lower extent, in stems and leaves.
Interaction	Homotetramer	Homotetramer	Homotetramer

Family & Domains	Belongs to the AdoMet synthase family.<br>**InterPro**:<br>IPR022631 ADOMET_SYNTHASE_Conserved Sites (119-129 aa) (GAGDQGHMFGY), 266-274 (GGGAFSGKD)<br><br>IPR022630 S-AdoMet_synt_C (240-381 aa)<br>IPR022629 S-AdoMet_synt_central (117 – 238 aa)<br>IPR022628 S-AdoMet_synt_N (4-101 aa)<br>IPR002133 S-AdoMet_synthetase 1-393 aa)<br>IPR022636 S-AdoMet_synthetase_sfam (3-389 aa)<br>**Panther**:<br>PTHR11964 PTHR11964, 1 hit<br>S-adenosylmethionine synthetase (1-393 aa)<br>**Pfam**:<br>PF02773 S-AdoMet_synt_C, 1 hit (240-382 aa)<br>PF02772 S-AdoMet_synt_M, 1 hit (117-238 aa)<br>PF00438 S-AdoMet_synt_N, 1 hit (3-101 aa)<br>**Supfam:** SSF55973 SSF55973, 3 hits<br>**Prosite:**<br>PS00376 ADOMET_SYNTHASE_1, 1 hit (119-129 aa) (GAGDQGhmfGY)<br>PS00377 ADOMET_SYNTHASE_2, 1 hit (266-274 aa) (GGGAFSgKD)	Belongs to the AdoMet synthase family.<br>**InterPro**:<br>IPR022631 ADOMET_SYNTHASE_Conserved Sites (119-129 aa) (GAGDQGHMFGY), 266-274 (GGGAFSGKD)<br><br>IPR022630 S-AdoMet_synt_C (240-381 aa)<br>IPR022629 S-AdoMet_synt_central (117 – 238 aa)<br>IPR022628 S-AdoMet_synt_N (4-101 aa)<br>IPR002133 S-AdoMet_synthetase (1-393 aa)<br>IPR022636 S-AdoMet_synthetase_sfam (3-388 aa)<br>**Panther**:<br>PTHR11964 PTHR11964, 1 hit<br>S-adenosylmethionine synthetase (1-393 aa)<br>**Pfam**:<br>PF02773 S-AdoMet_synt_C, 1 hit (240-382 aa)<br>PF02772 S-AdoMet_synt_M, 1 hit (117-238 aa)<br>PF00438 S-AdoMet_synt_N, 1 hit (3-101 aa)<br>**Supfam:** SSF55973 SSF55973, 3 hits<br>**Prosite:**<br>PS00376 ADOMET_SYNTHASE_1, 1 hit (119-129 aa) (GAGDQGhmfGY)<br>PS00377 ADOMET_SYNTHASE_2, 1 hit (266-274 aa) (GGGAFSgKD)	Belongs to the AdoMet synthase family.<br>**InterPro**:<br>IPR022631 ADOMET_SYNTHASE_Conserved Sites (119-129 aa) (GAGDQGHMFGY), 266-274 (GGGAFSGKD), 240-281 aa (IGGPHGDAGLTGRKIIIDTYGGWGAHGGGAFSGKDPTKVDRS)<br>IPR022630 S-AdoMet_synt_C (240-381 aa)<br>IPR022629 S-AdoMet_synt_central (118 – 238 aa)<br>IPR022628 S-AdoMet_synt_N (4-101 aa)<br>IPR002133 S-AdoMet_synthetase 1-393 aa)<br>IPR022636 S-AdoMet_synthetase_sfam (3-388 aa)<br>**Panther**:<br>PTHR11964 PTHR11964, 1 hit<br>S-adenosylmethionine synthetase (1-393 aa)<br>**Pfam**:<br>PF02773 S-AdoMet_synt_C, 1 hit (240-381 aa)<br>PF02772 S-AdoMet_synt_M, 1 hit (118-238 aa)<br>PF00438 S-AdoMet_synt_N, 1 hit (4-101 aa)<br>**Supfam:** SSF55973 SSF55973, 3 hits<br>**Prosite:**<br>PS00376 ADOMET_SYNTHASE_1, 1 hit (119-129 aa) (GAGDQGhmfGY)<br>PS00377 ADOMET_SYNTHASE_2, 1 hit (266-274 aa) (GGGAFSgKD)
3D structure databases	-	-	SMR: Q94FA4<br>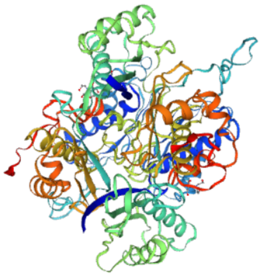

The full-length S-adenosylmethionine synthase protein sequences of *L.sativa* (A02J6KI88), *H. annuus* (G3LUV1) and *B. juncea* (Q94FA4) were aligned using Clustal Omega ver. 1.2.4 (https://www.ebi.ac.uk/Tools/msa/clustalo). Three domains of S-adenosylmethionine synthetase were indicated i.e N-terminal (IPR022628), central (IPR022629) and C-terminal (IPR022630).

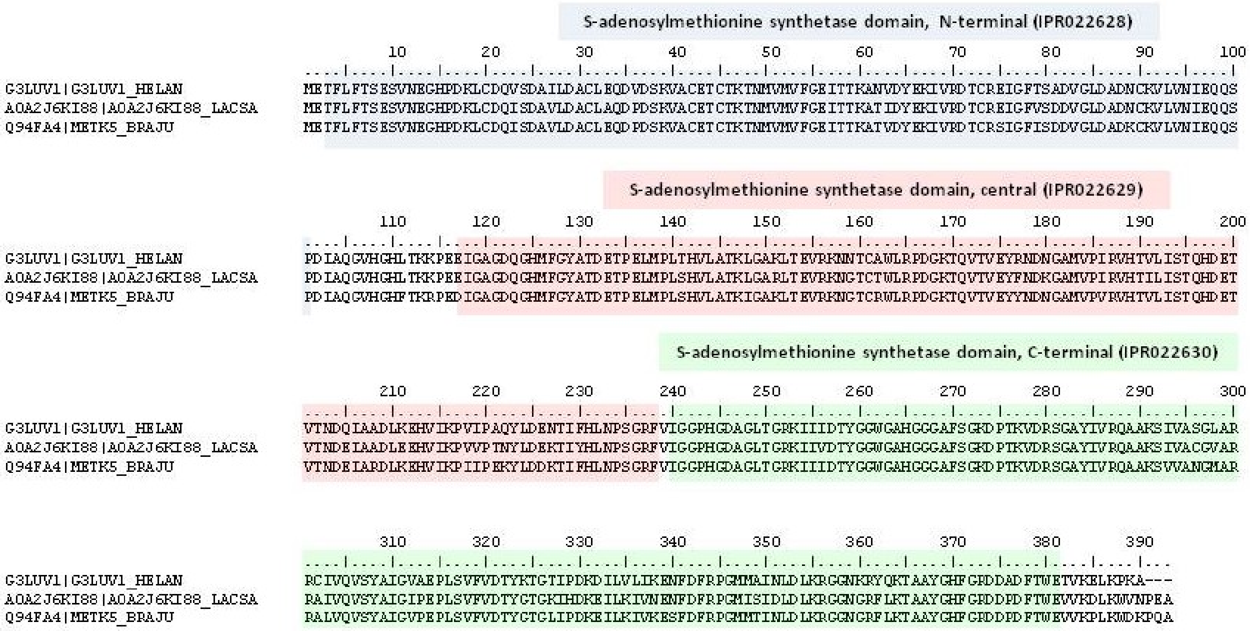

The protein sequence logo (http://weblogo.berkeley.edu) (presented below) representing the alignment of *L.sativa* (A02J6KI88), *H. annuus* (G3LUV1) and *B. juncea* (Q94FA4) confirmed that binding sites and regions of Mg, K, ATP and Met are conserved, increasing similarity in the S-AdoMet synthetase domains.

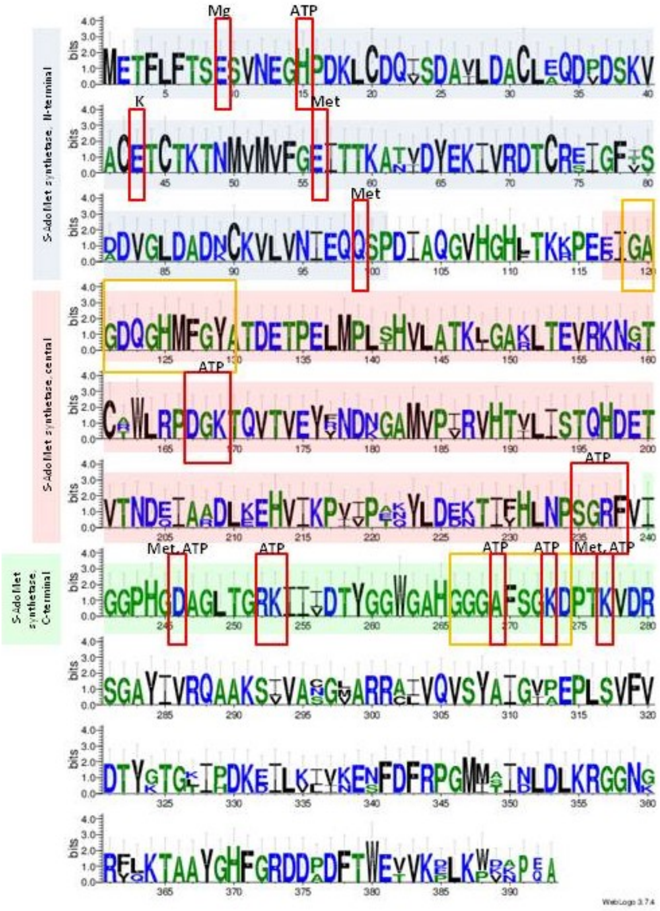

Supplementary data S4. The functional annotation of *samdmt* gene

The BLASTx search on NCBI (http://www.ncbi.nlm.nih.gov) againt the non-redundant (nr) protein database, default patrameters showed that *MAB_3787* (*SAMTase*) gene of *Lactuca sativa* L. var. *capitata* 'Melodion' c.v. (Acc. No. MT663550) has 100% identity to *L. sativa* uncharacterized protein LOC111890312 (Acc. No. XP_023742223.1, UniProtKB database A0A2J6JX40_LACSA), 72% identity to *A. chinensis var. chinensis* S-adenosyl-L-methionine-dependent methyltransferase (Acc. No. PSR99679.1, UniProtKB database: A0A2R6PZV1_ACTCC) and 70% identity to *V. vinifera* putative S-adenosyl-L-methionine-dependent methyltransferase (Acc. No. RVX14946.1, UniProtKB database: A0A438K157_VITVI). Comparative analysis of S-adenosyl-L-methionine-dependent methyltransferase proteins of *L. sativa*, *A. chinensis var. chinensis* and *V. vinifera* are presented below.

	Lactuca sativa	***Actinidia chinensis var. chinensis***	***Vitis vinifera***
UniProtKB	A0A2J6JX40<br>(A0A2J6JX40_LACSA)	A0A2R6PZV1<br>(A0A2R6PZV1_ACTCC)	A0A438K157<br>(A0A438K157_VITVI)
Protein name	Uncharacterized protein	S-adenosyl-L-methionine-dependent methyltransferase	Putative S-adenosyl-L-methionine-dependent methyltransferase
Sequence length (aa)<br>Mass (kDa)	329<br>37.014	359<br>40.470	352<br>39.647
Function	GO - Molecular function:<br>methyltransferase activity<br>GO - Biological process:<br>methylation	GO - Molecular function:<br>methyltransferase activity<br>GO - Biological process:<br>methylation	GO - Molecular function:<br>methyltransferase activity<br>GO - Biological process:<br>methylation
Molecule processing	-	Signal peptide: 1-20 aa<br>Mature peptide: 21-359 aa	Signal peptide: 1-21 aa<br>Mature peptide: 22-352 aa
Family & Domains	Belongs to the methyltransferase Ppm1/Ppm2/Tcmp family.<br>**InterPro**:<br>IPR011610 CHP00027_methylltransferase (54-240 aa)<br>IPR007213 Ppm1/Ppm2/Tcmp (54-240 aa)<br>IPR029063 SAM-dependent_MTases (54-240 aa)<br>**Pfam**:<br>PF04072 Leucine carboxyl methyltransferase (LCM) (44-223 aa)<br>Active site (predicted):<br>98 (R), 209 (Y)<br>**Supfam:**<br>SSF53335 SSF53335, 1 hit<br>**TIGRFAMs:**<br>TIGR00027 mthyl_TIGR00027, 1 hit	Belongs to the methyltransferase Ppm1/Ppm2/Tcmp family.<br>**InterPro**:<br>IPR011610 CHP00027_methylltransferase (80-271 aa)<br>IPR007213 Ppm1/Ppm2/Tcmp (69-253 aa)<br>IPR029063 SAM-dependent_MTases (67-279 aa)<br>**Pfam**:<br>PF04072 Leucine carboxyl methyltransferase (LCM) (69-254 aa)<br>Active site (predicted):<br>123 (R), 240 (Y)<br>**Supfam:**<br>SSF53335 SSF53335, 1 hit<br>**TIGRFAMs:**<br>TIGR00027 mthyl_TIGR00027, 1 hit	Belongs to the methyltransferase Ppm1/Ppm2/Tcmp family.<br>**InterPro**:<br>IPR011610 CHP00027_methylltransferase (80-271 aa)<br>IPR007213 Ppm1/Ppm2/Tcmp (66-251 aa)<br>IPR029063 SAM-dependent_MTases (70-267 aa)<br>**Pfam**:<br>PF04072 Leucine carboxyl methyltransferase (LCM) (66-251 aa)<br>**Supfam:**<br>SSF53335 SSF53335, 1 hit<br>**TIGRFAMs:**<br>TIGR00027 mthyl_TIGR00027, 1 hit

The full-length S-adenosyl-L-methionine-dependent methyltransferase of *L.sativa* (A0A2J6JQA9), *A. chinensis* var. *chinensis* (A0A2R6PZV1) and *V.vinifera* (A0A438K157) were aligned using Clustal Omega ver. 1.2.4 (https://www.ebi.ac.uk/Tools/msa/clustalo). Localization of SAM-dependent methylotransferase domain (IPR029063) was indicated on the alignignment enclosed below.

```
                                                                    SAM-dependent_Mtases (IPR029063)
                         10        20        30        40        50        60        70        80        90       100
                 ....|....|....|....|....|....|....|....|....|....|....|....|....|....|....|....|....|....|....|....|
A0A2J6JX40_LACSA ----------------------MAYEQNGRLPDHQNWP---RPELLYSEALRELHATIESDWDSVKRSACQTAAGRALWKHVVNDQLAELFAGETYLTN
A0A2R6PZV1_ACTCC MHALSLNFFSCISLSRPVLKLRIMSEEQNG-KPEEWAWPELKLPDLLLTDTVRKLHEAIESEWDSLRQSACQTAAGRALWKHVTHDQLAELLAGETYLRS
A0A438K157_VITVI ---MHYLFHFLLLILRNFLQLGDMSDEENG-PPEGSAWAELKLPDLLFTDTIRELHAAIGKEWDSLQQSACQTAAGRAMWKHVVHDPVAGLLAGETYLKS
                        110       120       130       140       150       160       170       180       190       200
                 ....|....|....|....|....|....|....|....|....|....|....|....|....|....|....|....|....|....|....|....|
A0A2J6JX40_LACSA LYEKIKNDRMNNAREVSGVILAVRTLWFESKLEAALESFG-GGAQVVLLGAGMDTRAYRLSCLKESDVYEIDFPEVLEMKETLLQTAINNS-----INPQ
A0A2R6PZV1_ACTCC LYEKIKKDRCRNAREVSGVMLAVRTLWFDSKIEAALSSFDGGGAQVVLLGAGMDARAYRLSCLKETDVFEVDFPEVLQMKTTTLQAATDSINDNQHVRVI
A0A438K157_VITVI FYDKINKDRLNNAREISGVILAVRTLWFDSKLEAALSSFNGGEAQVVLLGAGMDARAYRLSCLKESDVFEVDFPEVLQIKTSLLQAAMEPTNEYQHLRMT

                        210       220       230       240       250       260       270       280       290       300
                 ....|....|....|....|....|....|....|....|....|....|....|....|....|....|....|....|....|....|....|....|
A0A2J6JX40_LACSA SKSLTRIAADMREEDWFKKLQSSGFIPEKNTVWILEGIIYYLPHSQAMGVLKTIADNCSLTKTVLLADFMNKQSTTLSSSNSFHFYSDWPDHLLPTLGFS
A0A2R6PZV1_ACTCC AKSLNRVAADIRDNDWLQKLQISGFVPEKNTVWVLEGILYYLSHSHAIEVLKIIADNRALTRTVLLADFMNKSSTTLSS-SMFHFHSDWPDCLLPSLGFS
A0A438K157_VITVI AKSLTRVAADIREHDWVEKLQKSGFLQEKNTVWVLEGILYYLSHSQAMQVLKMIADNCGVTHTVLLADFMNKSSTMLST-STFHFYSDWPDHLLPTLGFS

                        310       320       330       340       350       360
                 ....|....|....|....|....|....|....|....|....|....|....|....|.
A0A2J6JX40_LACSA EVNLSQIGDPDADYGLLHDPLNLFNKLRGVPRSFQNHPDDGTPCCRLYLVQASGSPKTISS
A0A2R6PZV1_ACTCC EVKLSQIGDPDAHFGLLHDPLNLFNKLRSLPRSVQTHPDDGTPCRRLYFVQASSSPHQTTP
A0A438K157_VITVI DVKLSQIGDPDANFGLMHDPLNMFNKLRSLPRSVQTHPDDGTPCRRLYLVQASGSPR----
```

The protein sequence logo (http://weblogo.berkeley.edu) (presented below) representing the alignment of *L. sativa* (A0A2J6JQA9), *A. chinensis* var. *chinensis* (A0A2R6PZV1) and *V.vinifera* (A0A438K157) confirmed that active sites are conserved i.e. arginine (R) and tyrosine (Y).

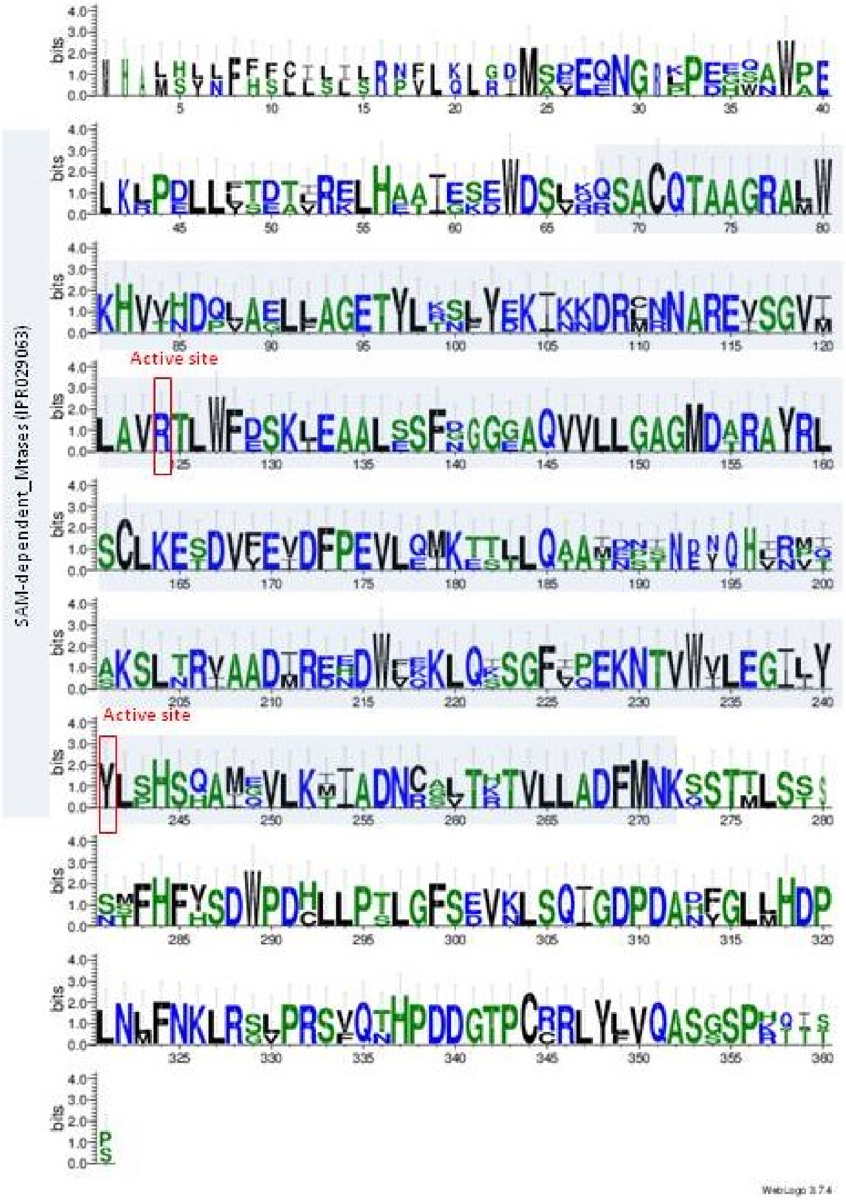

Supplement: Supplementary Data 1 — The functional annotation of per12-like i per64-like genes. [file Data_Sheet_1.pdf]
